# Activity Against ESKAPE Bacterial Pathogens of Pyrazole-Indol-Ruthenium(II) Complexes

**DOI:** 10.3390/antibiotics15070675

**Published:** 2026-07-09

**Authors:** Yahaira Cuenú-Ibargüen, Andrés Restrepo-Acevedo, Juan Felipe Zambrano-Bedoya, Isabel Murillo-Rodriguez, Carlos Felipe Mejía, Sandra Fabiola Alzate-Walteros, Gladymar Guadalupe Valenzuela-Ramirez, Gilmar López-Armenta, Federico del Rio-Portilla, Jesús Ricardo Parra-Unda, Fernando Cuenú-Cabezas, Ronan Le Lagadec

**Affiliations:** 1 Instituto de Química, Universidad Nacional Autónoma de México, Circuito Exterior s/n, Ciudad Universitaria, Ciudad de México 04510, Mexico; yahaira.cuenui@uqvirtual.edu.co (Y.C.-I.); acrestrepoa@gmail.com (A.R.-A.); jufezabe@comunidad.unam.mx (J.F.Z.-B.); murillo.isabel5@gmail.com (I.M.-R.); cfmejiag@comunidad.unam.mx (C.F.M.); federico.delrio@iquimica.unam.mx (F.d.R.-P.); 2 Programa de Química, Facultad de Ciencias Básicas y Tecnologías, Universidad del Quindío, Carrera 15 Calle 12 Norte, Armenia 630004, Quindío, Colombia; sfalzate@uniquindio.edu.co; 3 Unidad de Investigaciones en Salud Pública “Dra Kaethe Willms”, Facultad de Ciencias Químico Biológicas, Universidad Autónoma de Sinaloa, Ciudad Universitaria, Culiacán 80013, Sinaloa, Mexico; gladymarvzla.fcqb@uas.edu.mx (G.G.V.-R.); gilmar.fcqb@uas.edu.mx (G.L.-A.)

**Keywords:** Schiff bases, Pyrazole-indole ligands, Ruthenium, ESKAPE pathogens, Molecular docking calculations, DFT

## Abstract

This paper presents the synthesis and characterization of Schiff base ligands (L) derived from NH-pyrazole-indole and their ruthenium(II) complexes of the general formula [Ru(p-cymene)(L)Cl_2_]. The structural characterization of the ligands and ruthenium complexes was performed using a range of analytical methods, including infrared spectroscopy (IR), Raman spectroscopy, nuclear magnetic resonance (NMR) spectroscopy, ultraviolet-visible (UV-Vis) spectroscopy, elemental analysis, mass spectrometry(ESI-MS), density functional theory (DFT) calculations, and single-crystal X-ray diffraction crystallography for two of the four synthesized compounds. Schiff base ligands (L) derived from NH-pyrazole-indole and their ruthenium(II) complexes were evaluated against antibiotic-resistant bacteria. The effect of substituting the ligand with methoxy groups on biological activity was assessed. The antibacterial activity, Minimal Inhibitory Concentration (MIC), and Minimal Bactericidal Concentration (MBC) of the free ligands and the ruthenium compounds were measured against six bacterial isolates (*Staphylococcus aureus*, *Klebsiella pneumoniae*, *Acinetobacter baumannii*, *Pseudomonas aeruginosa*, *Escherichia coli*, *Shigella dysenteriae*) and two control strains (*Enterococcus faecalis* ATCC 29212 and *Enterobacter cloacae* ATCC 13047) from the ESKAPE group of highly antibiotic-resistant pathogens. The free ligands were inactive, whereas all four ruthenium complexes displayed notable antibacterial activity. In particular, compounds **Ru3** and **Ru4** exhibited higher activity against *Staphylococcus aureus* than the Trimethoprim control, with MIC values of 15.60 µg/mL, compared with an MIC > 64 µg/mL for Trimethoprim. The activity of those two complexes was similar to that of Gentamicin against this strain. Molecular docking calculations suggested a possible mechanism of action through binding of the complexes to the active site of *S. aureus* PBP2a.

## 1. Introduction

Antibiotic resistance has become a major public health problem with high rates of morbidity and mortality [1]. The World Health Organization (WHO) estimates that, by 2050, infections associated with antimicrobial resistance (AMR) will cause 10 million deaths annually [2]. In 2024, the WHO published an updated list of antibiotic-resistant microorganisms for which the development of new antimicrobial treatments is considered urgent. This list includes microorganisms belonging to the ESKAPE group: *Enterococcus faecium*, *Staphylococcus aureus*, *Acinetobacter baumannii*, *Pseudomonas aeruginosa* and *Enterobacter* spp. [3]. These pathogens are particularly relevant because of their intrinsic and extensive antibiotic resistance, as well as their ability to acquire multiple genes conferring multidrug resistance [4]. Given this problem, there is an urgent need to evaluate new compounds with antibacterial activity; metal complexes containing ruthenium and iron have emerged as promising candidates for drug development [5]. Mechanisms of action such as reactive oxygen species (ROS) generation, redox activation, DNA interactions, and depletion of substrates involved in vital cellular processes have been associated with metal-based compounds [6]. Additionally, various natural compounds have been shown to exhibit elevated antimicrobial effects [7,8,9].

The pyrazole-derived compounds examined in this study possess a highly versatile, stable structure that can be readily modified, thereby enabling multiple mechanisms of action against resistant bacteria. Pyrazole derivatives have been reported to exhibit potent inhibition of metallo-β-lactamases (MBLs) at low concentrations [10]. In synergistic studies, these compounds eliminate bacteria and resensitize a resistant strain of *K. pneumoniae* (K5) to the antibiotics meropenem and cephalexin, thereby restoring the effectiveness of common antibiotics [11]. This attribute renders them effective against resistant bacteria, such as *Staphylococcus aureus* and *Pseudomonas aeruginosa*.

The pyrazole-derived compounds under scrutiny in this study possess a highly versatile, stable structure that can be readily modified, thereby enabling multiple mechanisms of action against resistant bacteria. It has been established that one mechanism of action of pyrazole is to inactivate beta-lactamases. This process has two outcomes: first, it eliminates bacteria; second, it resensitizes bacteria, thereby ensuring that common antibiotics become effective again. Another proposed mechanism of action is that pyrazole facilitates crossing of the cell membrane. Finally, pyrazoles have been shown to be highly stable, capable of retaining their efficacy over extended periods. This attribute renders them effective against resistant bacteria, such as Staphylococcus aureus and Pseudomonas aeruginosa. Certain pyrazole derivatives have been shown to induce mitotic arrest, leading to apoptosis in rapidly dividing tumor cells, thereby making them valuable candidates for developing antimitotic agents [10,12,13,14]. Ruthenium complexes bound to pyrazole have gained a foothold in medicinal chemistry because pyrazoles coordinated to ruthenium are not merely spectator ligands; they modulate the metal center’s electronic density, enabling it to interact more effectively with biological molecules. The use of pyrazole-type ligands has been shown to stabilize the oxidation states of the complexes. Furthermore, pyrazole derivatives with a conjugated π system possess important photophysical properties that can increase the fluorescence of the complexes and facilitate π-π interactions, forming strong bonds with biological molecules and enhancing interactions with the membrane [15,16]. Schiff bases derived from NH-pyrazole are excellent ligands that coordinate well with transition metals, particularly ruthenium(II). Coordination occurs via the pyrazole nitrogen, which has an available lone pair and acts as a σ-donor. The pyrazole ring also has *p*π* orbitals that can accept electron density through backdonation from the ruthenium *d*π orbitals, thereby strengthening the ruthenium-pyrazole bond. In this work, we include Schiff bases derived from NH-pyrazole-indoles because we believe they promote electron flow through the π-cloud, enhancing electron acceptance by the pyrazole nitrogen [17,18,19,20].

Four imine-pyrazole ligands derived from indole have been prepared and coordinated to the [Ru^II^(η^6^-*p*-cymene)Cl_2_] moiety for evaluation against resistant bacteria, including six bacterial isolates (*Staphylococcus aureus*, *Klebsiella pneumoniae*, *Acinetobacter baumannii*, *Pseudomonas aeruginosa*, *Escherichia coli*, *Shigella dysenteriae*) and two control strains: *Enterococcus faecalis* ATCC 29212 and *Enterobacter cloacae* ATCC 13047. *Enterococcus faecalis* and *Staphylococcus aureus* are Gram-positive, whereas the others are Gram-negative. All are members of the ESKAPE group, a set of highly antibiotic-resistant nosocomial (hospital-acquired) pathogens.

## 2. Results

### 2.1. Synthesis of the Ligands and Ruthenium Complexes

**Scheme 1:**
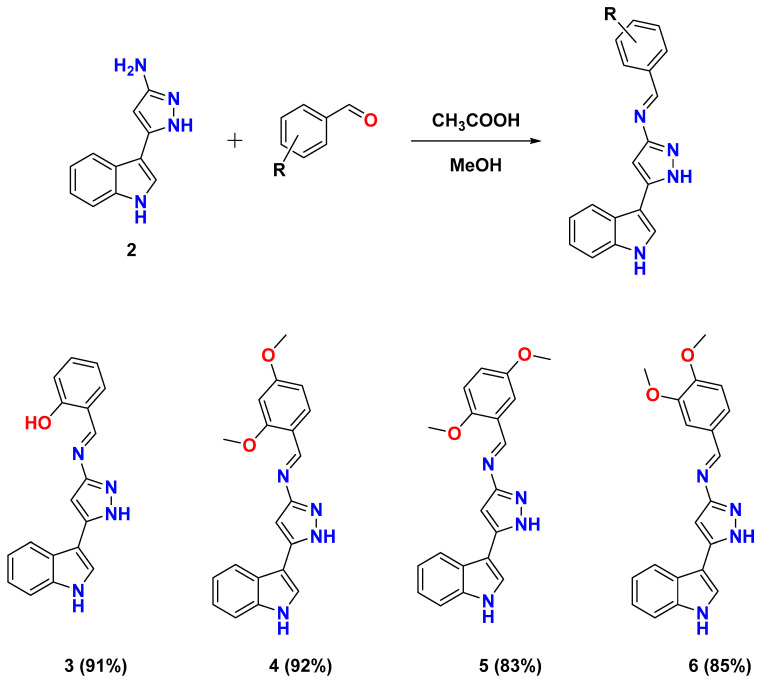
Synthesis of pyrazole-imine **3**–**6**.

**Figure 1:**
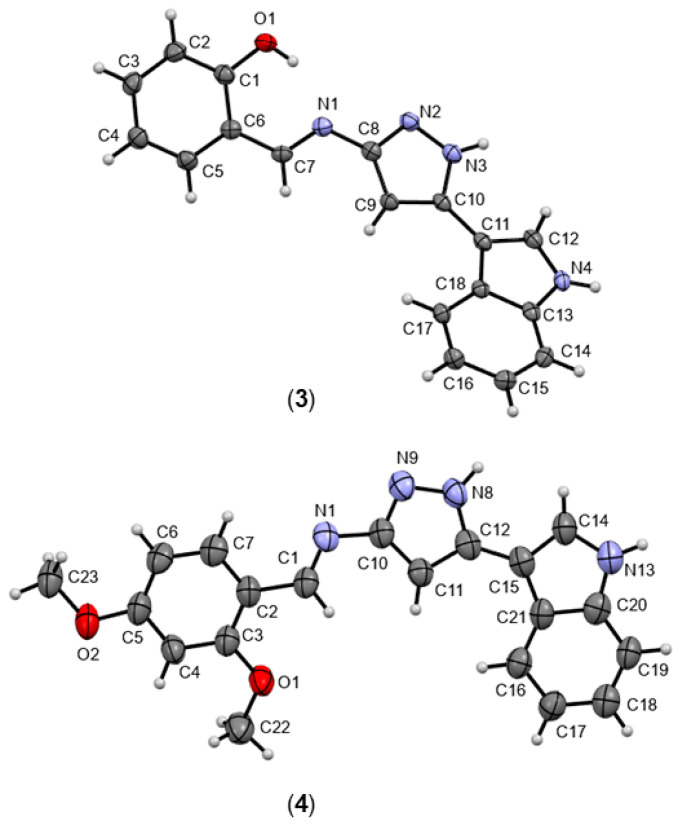
ORTEP diagram of pyrazole-imines **3** and **4**. Thermal ellipsoids set at 50% probability level.

Four *N*-donor ligands derived from imino-pyrazole-indole were synthesized by reacting the precursor 5-(1-*H*-indol-3-yl)-1-*H*-pyrazol-3-amine (**2**) with 2-hydroxybenzaldehyde, 2,4-dimethoxybenzaldehyde, 2,5-dimethoxybenzaldehyde, and 3,4-dimethoxybenzaldehyde, yielding compounds 2-[(*E*)-[5-(1*H*-indol-3-yl)-1*H*-pyrazol-3-yl]methyleneamino]phenol (**3**), (*E*)-*N*-(2,4-dimethoxyphenyl)-1-[5-(1*H*-indol-3-yl)-1*H*-pyrazol-3-yl]methanimine (**4**), (*E*)-*N*-(2,5-dimethoxyphenyl)-1-[5-(1*H*-indol-3-yl)-1*H*-pyrazol-3-yl]methanimine (**5**), and (*E*)-*N*-(3,4-dimethoxyphenyl)-1-[5-(1*H*-indol-3-yl)-1*H*-pyrazol-3-yl]methanimine (**6**), respectively, with yields ranging from 85% to 93%, as shown in Scheme 1. The ligands were fully characterized by NMR, IR, Raman, UV–vis, elemental analysis, DFT, ESI-MS (Figures S1–S68), and the structures of compounds **3** and **4** were confirmed by X-ray diffraction crystallographic studies (Figure 1) (see Section 4). The ESI-MS spectra reveal the precise molecular masses of the ligands. For compound **3**, the base peak corresponds to 285 *m*/*z*, consistent with the dissociation of the hydroxyl ion [M-OH]^+^, followed by the dissociation of the phenyl ion, yielding the ions [Ph]^+^ at 77 *m/z* and [M-105] at 198 *m*/*z*. For compounds **4**–**6**, the ESI-MS spectra show a peak at 347 *m*/*z* corresponding to the [M+1]^+^ ion.

Table 1 presents the crystal data, data-collection conditions, and structure-refinement details for compounds **3** and **4**. All hydrogen atoms were located from the difference Fourier maps and refined independently using isotropic displacement parameters. Suitable single crystals of compound **3** were obtained by slow evaporation of a methanol/acetonitrile solution, whereas crystals of compound **4** were grown by slow evaporation from ethyl ether. ORTEP representations of the molecular structures are shown in Figure 1. Compound **3** crystallized in the monoclinic system with space group C2, with two independent molecules in the asymmetric unit. The distances between the imine nitrogen and the pyrazole nitrogen atoms are 1.276 and 0.880 Å for the C3–N and N–H1 interactions, respectively. Compound **4** crystallized in the P21/n monoclinic space group and one molecule per asymmetric unit. The distances between the imine carbon and the pyrazole nitrogen atoms are 1.264 and 0.881 Å for the C3–N and N–H1 interactions, respectively. The H3–C3=N bond angles of the imine groups are 119.37° (**3**) and 118.86° (**4**), whereas the N–N=C15 bond angles of the pyrazole rings are 104.24° (**3**) and 105.18° (**4**) for the two independent molecules. These geometrical parameters are consistent with those reported in previous crystallographic studies from our research group, which has extensively investigated pyrazole-containing systems, confirming the preservation of the characteristic imine and pyrazole geometries in the solid state [21].

**Scheme 2:**
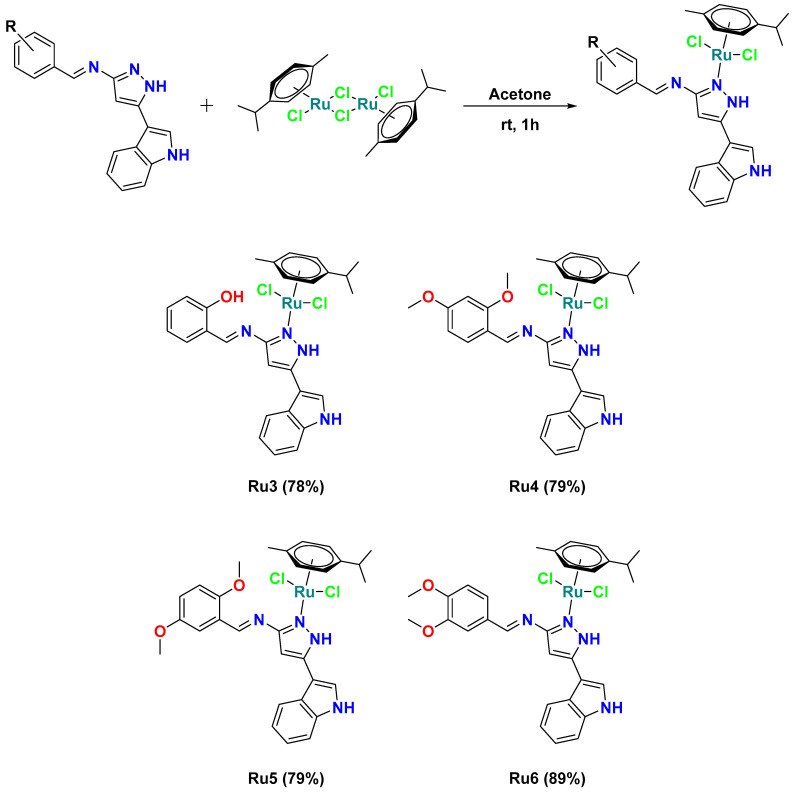
Synthesis of complexes **Ru3**–**Ru6**.

The coordination of these Schiff bases afforded the [Ru(η^6^-*p*-cymene)Cl_2_L] complexes (Scheme 2), with yields ranging from 78% to 89%. The ruthenium complexes were fully characterized by NMR, IR, Raman, UV–vis, elemental analysis, and ESI-MS and DFT calculations (Figures S34–S64, S67 and S69). The ESI-MS spectra reveal the precise molecular masses of the complexes. A peak at 617 *m*/*z* is observed in all complexes bearing methoxy groups, consistent with the loss of a methoxy group. The ^1^H NMR spectra of the ruthenium complexes reveal that some signals corresponding to the pyrazole and *p*-cymene ligands shift toward the high-field region, notably in the **Ru3**, **Ru4**, and **Ru6** complexes. These observations are consistent with the ligands being π-acceptors and good σ-electron donors, which allows ruthenium to transfer charge to the π-π* orbitals of the pyridine-type nitrogen in the pyrazole and to the π-π* orbitals of the *p*-cymene. This suggests that the pyrazole-indole-derived Schiff base ligands act as effective electron donors [22,23]. The methyl group protons in the *p*-cymene ring shift to a higher field upon coordination of the Schiff base ligands. This confirms that the pyrazole ligands act as excellent electron-density donors.

### 2.2. Antibacterial Activity of Compounds in the ESKAPE Bacterial Pathogens Group

The stability of the ruthenium complexes was evaluated in a phosphate-buffered saline (PBS)/DMSO system to assess their behavior under conditions relevant to the biological assays. Solutions of the complexes were prepared in PBS containing 0.1% DMSO at a final concentration of 10 μM, and their electronic absorption spectra were monitored for 24 h at room temperature. No significant changes were observed in either the position or intensity of the absorption bands during the monitoring period (Figure S70), indicating that the complexes remain largely intact in the PBS/DMSO medium. The spectral profiles suggest minimal decomposition under these conditions, with an estimated rate of less than 5% after 24 h. This stability is consistent with the requirements for biological evaluation in aqueous media and supports the use of these complexes in subsequent antibacterial studies. The corresponding free ligands were not examined under the same conditions due to their limited solubility in aqueous media, which precluded the acquisition of reproducible UV–vis spectra.

The antibacterial activity, Minimal Inhibitory Concentration (MIC), and Minimal Bactericidal Concentration (MBC) were evaluated against six bacterial isolates (*Staphylococcus aureus*, *Klebsiella pneumoniae*, *Acinetobacter baumannii*, *Pseudomonas aeruginosa*, *Escherichia coli*, *Shigella dysenteriae*) and two control strains: *Enterococcus faecalis* ATCC 29212 and *Enterobacter cloacae* ATCC 13047. *Enterococcus faecalis* and *Staphylococcus aureus* are Gram-positive, while the others are Gram-negative. All are members of the ESKAPE group, a set of highly antibiotic-resistant nosocomial (hospital-acquired) pathogens.

All four ruthenium compounds **Ru3**–**Ru6** exhibited antibacterial activity consistent with Gibbons, who proposed that compounds with favorable antibacterial activity should display MIC values not exceeding 64 µg/mL [24]. In contrast, the free ligands **3**–**6** showed no activity, with MIC values greater than 250 µg/mL (Table 2). Complexes **Ru4** and **Ru3** exhibited activity against two isolates, with **Ru4** showing MIC values of 62.5 µg/mL and 15.6 µg/mL against *Enterococcus faecalis ATCC 29212* and *Staphylococcus aureus*, respectively. Meanwhile, **Ru3** exhibited an MIC of 15.6 µg/mL against *Staphylococcus aureus* and 62.5 µg/mL against *Shigella dysenteriae*. Compounds **Ru5** and **Ru4** showed activity against a single isolate, with a MIC value of 62.5 µg/mL against *Staphylococcus aureus*. Overall, greater antibacterial activity was observed against Gram-positive bacteria. **Ru4** was active against *Enterococcus faecalis* ATCC 29212 and *Staphylococcus aureus*, while **Ru5** and **Ru3** were active against *Staphylococcus aureus*. In contrast, **Ru3** exhibited activity against Gram-negative bacteria *Shigella dysenteriae*. All data are shown in Table 2.

Regarding the control drugs used, compounds **Ru4**, and **Ru3** showed higher antibacterial activity than Trimethoprim against *Staphylococcus aureus*, with MIC values of 15.60 µg/mL compared with an MIC > 64 µg/mL for Trimethoprim. Compared with Gentamicin, compounds **Ru3** and **Ru4** exhibited a MIC of 15.60 µg/mL against *Staphylococcus aureus*, similar to the MIC of the control drug (16.0 µg/mL).

### 2.3. Molecular Docking Analysis

Penicillin-binding proteins (PBPs) are the primary molecular targets of β-lactam antibiotics because they bind radiolabeled penicillin, a property that initially facilitated their identification and characterization. These proteins are essential for maintaining the structural integrity of the bacterial cell wall. They are also the main targets of β-lactam antibiotics because of their catalytic activity in transpeptidation [25]. In particular, PBP2a from *Staphylococcus aureus* plays a fundamental role in the final stage of peptidoglycan assembly, a process essential for the integrity and viability of the bacterial cell. PBP2a is a class A enzyme involved in both cell wall synthesis and bacterial division, notably with a preferential localization at the cleavage septum [25]. Furthermore, PBP2a exhibits significantly reduced affinity for β-lactam antibiotics compared with other PBPs. This property allows *S. aureus* PBP2a to continue catalyzing cell wall synthesis even in the presence of penicillin-derived antibiotics, such as methicillin, oxacillin, nafcillin, and various cephalosporins. Consequently, PBP2a has been widely recognized as a promising therapeutic target for the development of specific inhibitors to treat infections caused by this resistant pathogen [26].

**Figure 2:**
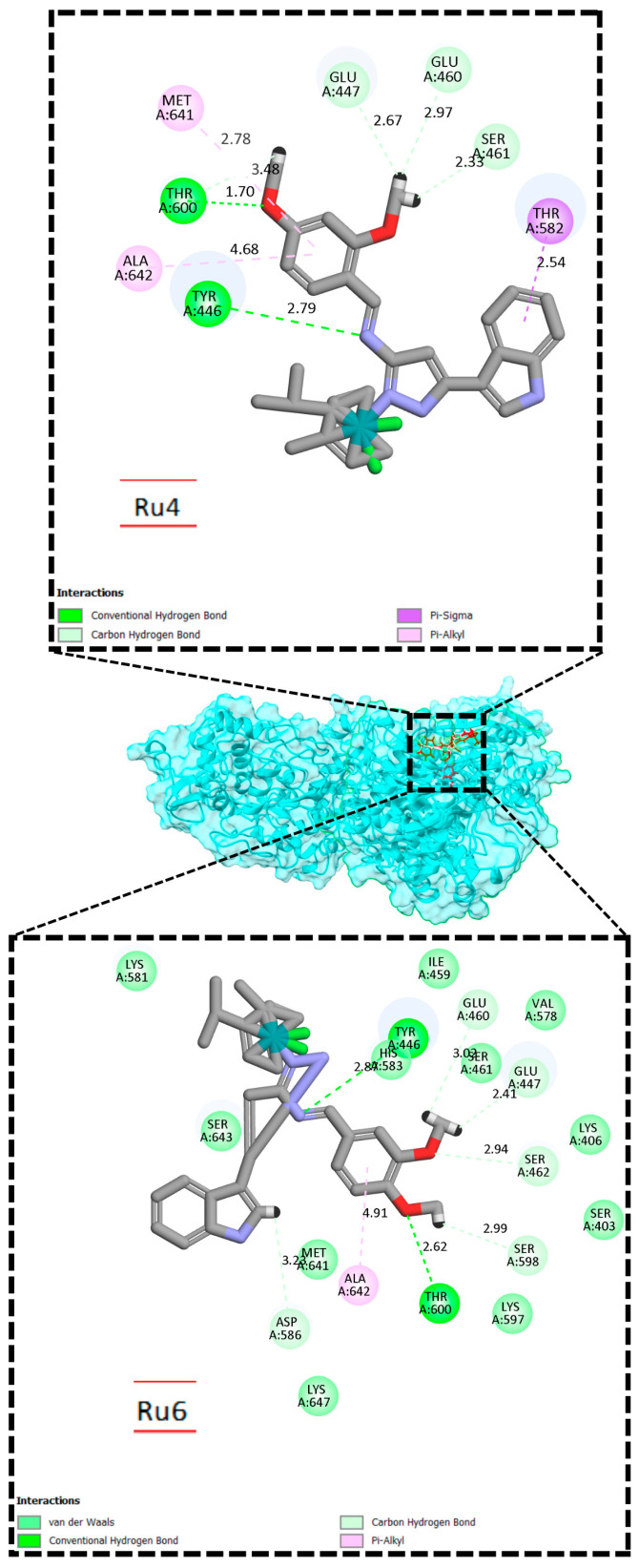
Binding of PBP2a with compounds **Ru4** and **Ru6**. It can be observed that both compounds bind to the same site and interact with residues in the active site.

Molecular docking analysis was performed on residues from the PBP2a protein’s active site and a second allosteric binding site. All four organometallic compounds interacted with the PBP2a active site (Figure 2). The binding energy ranged from −6.26 to −7.27 kcal/mol, compared with the control drugs, which showed affinity energies of −5.10 to −6.42 kcal/mol. Furthermore, compounds **Ru4** and **Ru6** exhibited more favorable binding energies, −7.05 and −7.27 kcal/mol, respectively, than **Ru3** and **Ru5** (Table 3). This is consistent with the in vitro findings, as these compounds exhibited lower MBC values against the tested strain than those observed for *S. aureus*. Therefore, these compounds showed a greater predicted ability to interact with the protein active site, supporting their potential as putative inhibitors. Compound **Ru4** exhibited various types of interactions (conventional hydrogen bonds, carbon-hydrogen bonds, π-σ, and π-alkyl). Notably, interactions were observed with residues in the active site, including hydrogen bonds with Tyr446 and Thr600 at distances of 2.79 Å and 1.70 Å, respectively. Additionally, a π–σ interaction with Thr582 was observed at a distance of 2.54 Å. On the other hand, compound **Ru6** showed interactions with residues in the active site, including hydrogen bonding with Tyr446 at 2.87 Å, Thr600 at 2.62 Å, and carbon-hydrogen bonding with Ser598 at 2.99 Å. Both compounds **Ru4** and **Ru6** exhibited potential inhibitory activity against PBP2a, a protein that is a pharmacological target due to its importance in bacterial survival and antibiotic resistance.

## 3. Discussion

The IR spectrum of compound **3** shows an N-H vibration of the indole group at 3401 cm^−1^ and an N-H vibration of the pyrazole group at 3387 cm^−1^. Theoretical calculations indicate that these N-H vibrations should be observed at 3522 and 3519 cm^−1^, respectively, with contributions of 99% and 100%, respectively (Table S2 and Figures S3 and S5), The IR and Raman frequencies and theoretical intensities were calculated using the B3LYP method. The method’s scaling factor was determined to be 0.960461 DFT-B3LYP/6–311++G. The correlation coefficients R^2^ are 0.9981 for IR and 0.9989 for Raman (Figures S6 and S7).

The experimental OH vibration occurs at 3149 cm^−1^, and the theoretical value is 3080 cm^−1^, with a contribution of 98%. The experimental hydroxyl vibration is observed as a very low-intensity broad band at 3149 cm^−1^, which is attributed to the formation of a strong intramolecular bond between the hydrogen of the hydroxyl group and the nitrogen of the azomethine functionality [21,27,28,29]. Additionally, compound **3** shows a C=N vibration of the azomethine group at 1621 cm^−1^, whereas theoretical calculations indicate that the C=N vibration should be observed at 1598 cm^−1^, with a contribution of 70%. The C=N vibration is observed at 1613 cm^−1^ in the experimental Raman spectrum of compound **3** and at 1598 cm^−1^ in the theoretical spectrum. For complex **Ru3**, the C=N vibration shifts to lower frequencies at 1617 cm^−1^ in IR and 1611 cm^−1^ in Raman (theoretical value of 1597 cm^−1^ in both cases). Experimental and theoretical data from infrared and Raman spectroscopy indicate that the C=N bond vibration of the azomethine group occurs at a lower frequency in the ruthenium complex than in the free ligand. This is consistent with the ligand acting as a good π-electron acceptor, resulting from charge transfer from the dπ orbitals of the ruthenium to the antibonding π orbitals of compound **3** (*p*π*←*d*π), strengthening the ruthenium–nitrogen bond. In contrast, for the ruthenium complexes containing methoxy-substituted ligands (**Ru4**–**Ru6**), a distinct trend is observed. In both experimental and theoretical infrared and Raman spectra, these complexes exhibit a shift toward higher vibrational frequencies for the C=N group compared to their respective free ligands (Tables S2 and S3). This shift indicates that these ligands act predominantly as strong sigma charge density donors [29]. The vibrational behavior of the Ru-N bond in infrared and Raman spectra confirms that the aromatic ring’s electron density directly modulates the coordination strength. Experimentally, the **Ru3** complex exhibits the highest stretching frequency in the IR region (473 cm^−1^), significantly surpassing the dimethoxylated systems: **Ru4** 427 cm^−1^, **Ru5** 416 cm^−1^ and **Ru6** 395 cm^−1^. This trend indicates a stronger Ru-N bond in the hydroxylated complex [30]. This phenomenon is corroborated by the Raman spectra, in which the **Ru3** complex retains the highest value, 465 cm^−1^, while the **Ru4**, **Ru5**, and **Ru6** complexes exhibit lower vibrational frequencies of 446, 445, and 398 cm^−1^, respectively. The observed similarity among these latter values suggests that, irrespective of the position of the methoxy groups, the electronic saturation of the ring inhibits metal backdonation to an almost equivalent degree. This tendency is further substantiated by theoretical DFT calculations. The Ru–N bond frequency for **Ru3** (468 cm^−1^) is systematically higher than that of the **Ru4** (452 cm^−1^), **Ru5** (447 cm^−1^), and **Ru6** (306 cm^−1^) systems. This agreement between experimental and theoretical data demonstrates that electronic modulation by the arene-ring substituents is the critical variable determining the strength of the metal-ligand bond.

The ^1^H NMR spectra of the ruthenium complexes indicate that the signals for OH, and NH, H-8, H-11 are shifted to the high-field region relative to the ligand signals (see Table S6 and Figure S1). This behavior is also observed in the H-12 signal in compounds **Ru3** and **Ru4**, but in compound **Ru5** it shifts to a lower field, and compound **Ru6** appears to exhibit the same chemical shift as ligand **6**. This suggests that the pyrazole-indole-derived Schiff base ligands act as effective electron acceptors. The protons of the methyl groups belonging to the *p*-cymene group shift to a higher field when the Schiff base ligands are coordinated. This shows that the ligands act as excellent electron-density donors. Therefore, it can be concluded that these ligands are good σ-donors and π-acceptors, particularly the ligand **3**. The **Ru4** and **Ru6** compounds exhibit increased chemical shifts at high fields for specific protons in the *p*-cymene, suggesting that ligands 4 and 6 act as effective σ-type electron-density donors. Consequently, it can be inferred that the **Ru4** and **Ru6** complexes function as efficient σ-donors and strong π-acceptors. Table S4 and Figures S66–S69 show the experimental and theoretical UV-vis data of the ligands and ruthenium complexes. A general characteristic is the similarity in the wavelengths of the transitions, as well as the fact that the ruthenium complexes exhibit metal-to-ligand charge transfer bands, which corroborates the observations made in the IR, Raman, and NMR spectra.

As shown in Table S6, Schiff bases **3**–**6** and their ruthenium complexes (**Ru3**–**Ru6**) exhibit varying degrees of reactivity. The position of the methoxy groups affects the reactivity of the molecules, particularly in the ruthenium complexes. For instance, the homo orbital of the **Ru6** complex has the lowest energy (−7.13 eV), followed by **Ru4** (−6.88 eV), **Ru5** (−6.76 eV), and **Ru3** (−5.85 eV). This results in the **Ru4** and **Ru6** compounds having the largest energy gaps, 6.12 and 6.06 eV, respectively. Additionally, **Ru6** has the lowest chemical potential (−4.10 eV) and the highest electronegativity, enabling it to retain its electrons more strongly and attract electrons. Conversely, the **Ru3** compound exhibits a comparatively small energy gap (3.52 eV), rendering it highly reactive and proficient at donating electron density. However, it has a low chemical potential, indicating its ability to both donate electrons and accept electron density. These findings are consistent with those observed in theoretical and experimental IR, Raman, and NMR studies.

The use of transition metal complexes as antibacterial agents is an emerging field in biomedical research. A total of four ruthenium complexes were evaluated, and all exhibited activity against ESKAPE group bacteria. The free ligands **3**–**6** showed no activity against the bacteria studied. The lowest MIC values observed for the ruthenium compounds were 15.60 µg/mL (**Ru3** and **Ru4** against *Staphylococcus aureus*). Organometallic ruthenium compounds with MIC values as low as 3.125 µg/mL against *Staphylococcus aureus* ATCC 29213 have been reported; this series of complexes has also been associated with low toxicity to human cells [31]. Such ruthenium complexes have gained prominence primarily as anticancer agents and, more recently, as antibacterial agents against a variety of ESKAPE pathogens, owing to their public health relevance. Our new ruthenium compounds exhibited activity against both Gram-positive bacteria (*Enterococcus faecalis* ATCC 29212 and *Staphylococcus aureus*) and Gram-negative bacteria (*Escherichia coli* and *Shigella dysenteriae*). Although the precise antibacterial mechanism of the complexes reported here remains to be established, previous studies on ruthenium complexes have proposed several possible mechanisms, including inhibition of biofilm formation with values ranging from 1.5 to 12.5 µg/mL [32]. Ruthenium complexes have been reported to be internalized by bacterial cells and target intracellular bacterial DNA in both Gram-positive (*Enterococcus faecalis* and *Staphylococcus aureus*) and Gram-negative (Escherichia coli and *Pseudomonas aeruginosa*) bacteria [33]. Our complexes did not exhibit activity against *Acinetobacter baumannii*, unlike a dinuclear ruthenium(II) polypyridyl complex reported to be highly active against ESKAPE pathogens, including *Acinetobacter baumannii* and *Pseudomonas aeruginosa* [34]. In addition to their antibacterial activity, some ruthenium complexes have been considered safe in previous studies, where moderate cytotoxicity and low toxicity have been reported against human ovarian adenocarcinoma (A2780), a cisplatin-resistant variant of A2780 (A2780cisR), and normal human prostatic epithelium (PNT2) cell lines [31].

Previous studies have highlighted the importance of inorganic and organometallic compounds as potential antibacterial agents against *S. aureus* by inhibiting proteins or enzymes involved in bacterial survival and antibiotic resistance. Among the compounds evaluated in this study, **Ru4** and **Ru6** showed the highest predicted affinity for PBP2a, even surpassing that of Ceftaroline, a direct inhibitor of this protein [35]. However, it is important to note that these values correspond to theoretical binding affinities derived from molecular docking and do not necessarily reflect the compounds’ actual inhibitory activity. The catalytic site of PBP2a was considered the target for molecular docking analyses with flavonoids and compounds used as negative and positive controls. After analyzing the interactions of nitrocefin, penicillin G, and methicillin with residues located in the catalytic domain of PBP2a, 14 key residues were identified in the active site of PBP2a: Ser337, Lys340, Ser403, Lys406, Tyr446, Ser462, Asn464, Thr500, Ser548, Gly549, Ser598, Gly599, Thr600, and Met681. Consequently, these amino acids were considered the receptor site for molecular docking analyses to identify potential inhibitors targeting the enzyme’s active site [26]. The affinity values obtained for the ruthenium complexes evaluated against PBP2a ranged from −6.26 to −7.27 kcal/mol, indicating a moderate capacity to interact with the protein’s active site. The more favorable binding of the **Ru4** and **Ru6** complexes compared to **Ru5** can be attributed to the substitution pattern on the aromatic ring. In **Ru4** (meta) and **Ru6** (ortho), the ether oxygen atoms are favorably positioned to form short-range hydrogen bonds with key catalytic residues such as Tyr446 and Thr600. Conversely, the substitution in **Ru5** likely orients the functional groups away from these specific interaction sites, thereby lowering the binding energy. Furthermore, the [Ru(p-cymene)] unit provides structural rigidity. These results are comparable to those reported for rhenium complexes, whose affinity values ranged from -6.7 to -9.8 kcal/mol for PBP2a [36]. While the rhenium complexes showed higher predicted binding affinities, the ruthenium complexes evaluated in this study exhibited affinity values within a similar range and established interactions with key residues of the catalytic site, particularly Tyr446 and Ser598. Therefore, the results suggest that these ruthenium complexes possess molecular recognition capacity for PBP2a and warrant further studies to evaluate their inhibitory potential and mechanism of action.

## 4. Materials and Methods

All chemicals and solvents used (analytical grade) were purchased from Sigma- Aldrich and Across without further purification (St Louis, MO, USA). The melting points were determined on a Büchi melting point apparatus (Flawil, Switzerland). Infrared spectra were measured in an Alpha ATR spectrometer from Bruker. Raman spectra were recorded using a Horiba XploRA Plus confocal micro-spectrometer (Palaiseau, France) in an upright microscope configuration. The NMR spectra were recorded at 25 °C on a Bruker Avance 500 spectrometer (Billerica, MA, USA, EE.UU) operating at 400 MHz for ^1^H and 100 MHz for ^13^C in deuterated acetone or DMSO. NMR data are reported as follows: chemical shift (δ ppm), multiplicity (s = singlet, d = doublet, t = triplet, hept = heptuplet, m = multiplet, b = broad), integration, assignment, and coupling constant. The atoms were assigned according to the numbering scheme described in Figure S1. The mass spectra were obtained using a SHIMADZU GCMS-2010-DI-2010 spectrometer equipped (Kioto, Japan) with a direct input probe operating at 70 eV. Electrospray ionization mass spectrometry (ESI-MS) was performed using a Bruker Esquire spectrometer (Billerica, MA, USA, EE.UU). The UV–vis absorption spectra were obtained in the 200–600 nm range using a Shimadzu UV–vis 160 spectrophotometer (Kioto, Japan). Microanalyses were performed on an Agilent CHNS elemental analyzer (Santa Clara, CA, USA).

### 4.1. Stability Studies

Stability studies were performed using a Shimadzu 2600 UV−vis spectrophotometer (Kioto, Japan) at 37 °C from 1 × 10^−5^ M solutions of each compound in PBS (Dulbecco’s Phosphate-Buffered Saline, Dominique Dutscher SAS (Bernolsheim, France), without magnesium and calcium) with 0.1% DMSO. Spectra were recorded every hour for 8 h.

### 4.2. Crystallography

Single crystals suitable for X-ray diffraction were obtained for **3** by slow evaporation at room temperature from a methanol/acetonitrile mixture (9:1 mL), while crystals of **4** were obtained by slow evaporation from a diethyl ether solution. Data were collected at 298 K on a Bruker Apex-II CCD diffractometer (Billerica, MA, USA) using monochromatic graphite Mo Kα radiation (0.71073 Å). Cell determination and final cell parameters were obtained for all reflections using the Bruker SAINT-Plus (2007, Bruker, Billerica, MA, USA) software included in APEX2 suite [37]. The integration and scaling of the data were carried out using the Bruker SAINT software [37]. For compounds **3** and **4**, weak high-angle diffraction, combined with multiple components and some degree of disorder, resulted in a large number of refined parameters and a poor data-to-parameter ratio, which led to the observed low C–C precision. However, the identity of the structures was clearly established without issue. The CIF files have been deposited in the Cambridge Structural Database under CCDC 2549434 (accessed on 25 April 2026) for 3 and CCDC 2549435 for 4 (accessed on 25 April 2026). Copies of the data can be obtained, free of charge, at www.ccdc.cam.ac.uk.

### 4.3. Reactivation of Bacterial Isolates

The isolates were removed from the −80 °C deep freezer for partial thawing. They were then placed at −20 °C for 15 min, followed by thawing at room temperature for 10 min. The isolates were cultured in brain-heart infusion (BHI) medium and incubated at 37 °C for 24 h. The strains were plated onto MacConkey agar, mannitol salt agar, and blood agar, incubated at 35 °C for 18–20 h, and then stored at 4 °C. The biochemical profile of the strains was determined. Gram staining was also performed. After reactivation and identification of the strains, susceptibility profiles were confirmed using the Kirby-Bauer method.

### 4.4. Determination of Antibacterial Activities Using the Broth Microdilution Method

Antibacterial activity was evaluated using the broth microdilution method [38]. The minimum inhibitory concentration (MIC) and minimum bactericidal concentration (MBC) were determined. Trimethoprim and gentamicin were used as control drugs. *Enterococcus faecalis* ATCC 29212 and *Enterobacter cloacae* ATCC 13047 were used as control strains. Trimethoprim and gentamicin were used as reference drugs to compare the MIC and MBC values obtained against the isolates. The assay was conducted in the same manner as for the compounds, following CLSI guidelines [38]. Concentrations used were 64 μg/mL, 16 μg/mL, 8 μg/mL, 4 μg/mL, 2 μg/mL, 1 μg/mL, 0.5 μg/mL, 0.25 μg/mL, and 0.12 μg/mL.

### 4.5. Minimum Inhibitory Concentration (MIC)

Bacterial isolates were subcultured on TSA medium and incubated for 18–20 h at 35 °C to reach the logarithmic phase. A concentrated solution of each compound (500 μg/mL) was prepared using 10% DMSO and water as the solvent. Dilutions were made to obtain the concentrations to be evaluated: 250 μg/mL, 62.5 μg/mL, 15.6 μg/mL, 3.9 μg/mL, 0.97 μg/mL, 0.24 μg/mL, and 0.06 μg/mL. The highest concentration was sterilized by filtration through a syringe filter with a 0.20 μm PTFE membrane and a 25 mm diameter. A suspension of the inoculum was prepared in saline solution (0.85% *w*/*v*), and its turbidity was adjusted to 0.5 on the McFarland scale (1 × 10^8^ CFU/mL; %T = 62.7 ± 0.5) at a wavelength of 530 nm. A 0.1 mL aliquot of this suspension was added to 9.9 mL of Mueller-Hinton broth to obtain a working inoculum (1 × 10^6^ CFU/mL). The assay was performed in sterile 96-well U-bottom ELISA plates with lids. 50 μL of the reagent and 50 μL of the inoculum were added. The assay was performed in triplicate for each strain, including a positive control (inoculum and solvent) and a negative control (Mueller-Hinton broth and reagent). After adding the controls and samples, the ELISA plate was capped, sealed with parafilm, and incubated at 37 °C for 18–20 h. The MIC was determined visually, based on the absence of growth at the bottom of the well or turbidity in the medium after incubation.

### 4.6. Minimum Bactericidal Concentration (MBC)

To determine the MBC, MacConkey agar plates for Gram-negative bacteria and TSA agar plates for Gram-positive bacteria were used. These plates were inoculated in triplicate with the inoculum from each well where no microbial growth was observed, including those at the MIC, and incubated at 37 °C for 18 to 20 h. The concentration of the compound on the plate where no growth was observed in at least two of the three replicates was established as the MBC value.

### 4.7. Molecular Docking

The three-dimensional (3D) structures of the ligands were drawn in ChemDraw v23.1.1 and subsequently saved in Structure Data File (.sdf) format, and the control drugs (Ceftaroline, Gentamicin, and Trimethoprim) were downloaded from the PubChem database (https://pubchem.ncbi.nlm.nih.gov/, accessed on 25 May 2026). Ligand and drug preparation were performed using MOE v.2014.09.01 (Chemical Computing Group, Montreal, QC, Canada), where Gasteiger partial charges were assigned to the drugs and AM1-BCC to the organometallic compounds. Subsequently, the molecular geometry and the orientation of hydrogen atoms and electron pairs were automatically corrected to obtain chemically stable configurations. Energy minimization was performed using the MMFF94 force field for the drug control and the AMBER12:EHT force field for the organometallic compounds, under non-periodic conditions. During optimization, the orientations of the hydroxyl groups and hydrogen atoms were adjusted to obtain stable conformations. The crystal structure of the penicillin 2a-binding protein (PDB ID: 4CJN) was obtained from the Protein Data Bank. The receptor was prepared by first removing non-standard molecules and water molecules from the structure. Protein preparation and docking calculations were performed using MOE v.2014.09.01 (Chemical Computing Group, Montreal, QC, Canada). The protein was prepared according to the standard structure preparation protocol (Calculate, Structure Preparation, 3D Protonation, and Correct); missing hydrogen atoms were added, and the protein was protonated under physiological conditions at 310 K. Automatic correction was also performed. The docking analysis was directed at the enzyme’s active site, specifically at residues Ser337, Lys340, Ser403, Lys406, Tyr446, Ser462, Asn464, Thr500, Ser548, Gly549, Ser598, Gly599, Thr600, and Met681, as well as residues Y105 and Y297, which form a second allosteric binding site. The analysis was performed using the Triangle Matcher algorithm. Evaluation was conducted using London dG, retaining the 100 best poses, followed by refinement using the forcefield method and re-evaluation with the GBVI/WSA dG scoring function, retaining the 30 best poses. The most favorable docking conformation was selected based on the S-score value (kcal/mol).

### 4.8. DFT Calculations

The geometry of all ligands and metal complexes was optimized at the density of functional theory (DFT) level. The calculations were carried out using the Gaussian 09 program packet 09, with the B3LYP functional for ligands and CAM- B3LYP functional for metal complexes [39,40,41,42]. The LANL2DZ relativistic effective core potential basis set [42,43] was used for the ruthenium atom, and the 6-311G(d,p) basis set was used for the lighter atoms (C, N, O, Cl, H). Theoretical vibrational spectra of imines (**3**–**6**) and their ruthenium complexes (**Ru3**–**Ru6**) were interpreted by means of Potential Energy Distributions (PED) using the VEDA 4 program [44]. The electronic properties of the metal complexes were investigated using a TD-DFT (time-dependent density functional theory) approach. UV-vis spectra of all compounds were simulated in DMSO [19]. The molecular orbital composition, UV-vis spectra and assignment of electronic transitions were extracted from the output files using GaussSum 3.0 software [45].

### 4.9. Synthesis of 3-(1H-Indol-3-yl)-3-oxopropanone Nitrile (***1***)

The title compound was synthesized following a modified methodology described in the literature [46]. Cyanoacetic acid (2.1 g; 24.7 mmol) was added to 7 mL of acetic anhydride, and the reaction mixture was maintained at 55–60 °C for 15 min. The temperature was raised to 80 °C, indole (3.0 g, 25.6 mmol) was added, and the mixture was allowed to react for 30 min before being cooled in an ice bath. Compound **1** precipitated as a white solid, which was isolated by filtration and washed with cold ethanol (77% yield).

### 4.10. Synthesis of Pyrazole-Amine (***2***)

A mixture of 3-(1*H*-indol-3-yl)-3-oxopropanone nitrile (5.4 mmol) and 80% hydrazine monohydrate (27.2 mmol) in 50 mL of ethanol was heated to 80 °C for 24 h [46]. The solution was allowed to cool in an ice bath, and the resulting solid was filtered and washed with cold distilled water (77% yield).

### 4.11. Synthesis of Pyrazole-Imines (***3***–***6***)

The pyrazole-imine derivatives were prepared following reported methodologies [47,48]. Aminopyrazole **2** (50 mg, 25.2 mol) and the corresponding aldehyde were added in 5 mL of ethanol. The reaction mixture was stirred at 25 °C for 12 h. The resulting solids were washed with cold diethylether. The solids were filtered and dried under vacuum.

***(****E)-2-(((5-(1H-indol-3-yl)-1H-pyrazol-3-yl)imino)methyl)phenol (***3***)*: Yellow solid, 70 mg, yield: 91%. Anal. Calc. For C_18_H_14_N_4_O: %C 71.51, %H 4.67, %N 18.53; found: %C 71.05, %H 4.66, %N 18.35. mp 257–259 °C. MS (70 eV) *m*/*z* (%) 302 [M^+^] (95.75); 285 [M^+^-17] (100.0); 198 [M^+^-104] (2.47); 183 [M^+^-119] (9.53); 155 [M^+^-147] (16.67); 143 [M^+^-159] (36.09);128 [M^+^-174] (8.64); 117 [M^+^-185] (1.58); 105 [M^+^-197] (5.32); 77 [M^+^-225] (14.44); 40 [M^+^-262] (1.23). FTIR (ATR) ν (cm^−1^): 3401 (ν-OH); 3401 (ν-NH_indole_); 3149 (ν-OH); 3102 (ν-NH_Py_); 3000 (ν-CH_ph_); 1621 (ν-C=N); 1598(ν-C=C_indole_). Raman (ATR) ν (cm^−1^): 1613 (ν-C=N); 1580 (ν-C=C_indole_). UV–vis (DMSO, λ_max_ nm) (log ε): λ_1_ 287 (4.09); λ_2_ 343 (4.02). ^1^H NMR (300 MHz, acetone-*d_6_*, δ in ppm) 6.96 (s, 1H, H-12); 6.99 (t, 1H, H-10, ^3^*J* = 8.2); 7.01 (td, 1H, H-9, ^3^*J* = 7.48, ^4^*J* = 0.9); 7.16 (t, 1H, H-11, ^3^*J* = 7.4); 7.21 (t, 1H, H-7, ^3^*J* = 7.5); 7.42 (d, 1H, H-30, ^3^*J* = 8.4); 7.49 (d, 1H, H-8, ^3^*J* = 7.8); 7.65 (dd, 1H, H-6, ^3^*J* = 7.7, ^4^*J* = 1.5); 7.84 (*s*, 1H, H-5); 7.98 (bs, 1H, H-4,); 9.23 (s, 1H, H-3); 11.49 (s, 1H, H-2); 12.99 (**s**, 1H, H-1); 13.31 (s, 1H, OH). ^13^C NMR (75 MHz, acetone-*d_6_*, δ in ppm): 92.22 (C-12); 105.45 (C-22); 112.46 (C-7); 117.09 (C-30); 119.84 (C-4); 120.35 (C-10); 122.38 (C-9); 123.86 (C-5); 124.85 (C-21); 124.89 (C-20); 132.72 (C-6); 133.42 (C-8); 136.84 (C-19); 139.65 (C-18); 157.35 (C-16); 160.73 (C-15); 162.85 (C-3).

*(E)-N-(5-(1H-indol-3-yl)-1H-pyrazol-3-yl)-1-(2,4-dimethoxyphenyl)methanimine (***4****): Yellow solid, yield: 92%. Anal. Calc. For C_20_H_18_N_4_O_2_: %C 69.35, %H 5.24, %N 16.17; found: %C 67.94, %H 5.30, %N 15.63. mp 203–206 °C (decomp). MS (ESI) *m*/*z* 347 [C_20_H_18_N_4_O_2_+H]^+^. FTIR (ATR) ν (cm^−1^): 3364 (ν-NH_indole_); 3265 (ν-NH_Py_); 3007 (ν-CH_ph_); 2944 (ν_as_-CH_3_); 2886 (ν_s_-CH_3_); 1600 (ν-C=C); 1621 (ν-C=N). Raman (ATR) ν (cm^−1^): 1604 (ν-C=N); 1583 (ν-C=C_indole_). UV–vis (DMSO, λ_max_ nm) (log ε): λ_1_ 287 (4.02); λ_2_ 340 (4.05). ^1^H NMR (75 MHz, DMSO-*d_6_*, δ in ppm) 3.86 (s, 3H, H-14); 3.93 (s, 3H, H-13); 6.68 (m, 3H, H-10, H-11, H-12); 7.16 (m, 2H, H-9, H-8); 7.45 (bs,1H H-7); 7.81 (s, 1H, H-6); 7.94 (bs, 1H, H-5); 8.02 (d, 1H, H-4, ^3^*J* = 8.62); 9.15 (s, 1H, H-3); 11.45 (s, 1H, H-2); 12.74 (s, 1H, H-1). ^13^C NMR (300 MHz, DMSO-*d_6_*, δ in ppm): 55.32 (C-14); 56.01 (C-13); 93.03 (C-12); 98.67 (C-11); 107.02 (C-22 and C-10);112.25 (C-9); 112.35 (C-8); 117.80 (C-20); 119.91 (C-7); 120.30 (C-5); 123.72 (C-6); 124.84 (C-21); 128.40 (C-4); 136.82 (C-19); 154.78 (C-3); 154.83 (C-18); 160.47 (C-17); 161.18 (C-16); 163.97 (C-15).

*(E)-N-(5-(1H-indol-3-yl)-1H-pyrazol-3-yl)-1-(2,5-dimethoxyphenyl)methanimine (***5***)*: Yellow solid, yield: 83%. Anal. Calc. For C_20_H_18_N_4_O_2_: %C 69.35, %H 5.24, %N 16.17; found: %C 69.01, %H 5.38%N 16.18. mp 202–204 °C (decomp). MS (ESI) m/z 347 [C_20_H_18_N_4_O_2_+H]^+^. FTIR (ATR) ν (cm^−1^): 3313 (ν-NH_indole_); 3114 (ν-NH_Py_); 3001 (ν-CH_ph_); 2941 (ν_as_-CH_3_); 2832 (ν_s_-CH_3_); 1577 (ν-C=C_indole_); 1615 (ν-C=N). Raman (ATR) ν (cm^−1^): 1608 (ν-C=N); 1586 (ν-C=C_indole_). UV–vis (DMSO, λ_max_ nm) (log ε): λ_1_ 283 (4.12); λ_2_ 365 (4.04). ^1^H NMR (300 MHz, DMSO-*d_6_*, δ in ppm) 3.79 (s, 3H, H-14); 3.88 (*s*, 3H, H-13); 6.75 (s, 1H, H-12); 7.10 (m, 4H, H-7, H-9, H-10, H-11); 7.48 (d, 1H, H-8, ^3^*J* = 7.5); 7.59 (s, 1H, H-6); 7.82 (s, 1H, H-5); 7.97 (bs, 1H, H-4); 9.23 (s, 1H, H-3); 11.46 (s, 1H, H-2); 12.85 *(s*, 1H, H-1). ^13^C NMR (75 MHz, DMSO-*d_6_*, δ in ppm): 55.94 (C-14); 56.75 (C-13); 93.21 (C-12); 105.73 (C-22); 110.07 (C-6); 112.34 (C-8); 114.10 (C-7); 119.92 (C-10); 119.98 (C-4); 120.29 (C-11); 122.27 (C-9); 123.80 (C-5); 124.87 (C-20); 125.06 (C-21); 136.82 (C-19); 139.28 (C-18); 153.71 (C-17); 154.27 (C-16); 154.35 (C-3); 160.01 (C-15).

(*E)-N-(5-(1H-indol-3-yl)-1H-pyrazol-3-yl)-1-(3,4-dimethoxyphenyl)methanimine (***6***)*: White solid, yield: 85%. Anal. Calc. For C_20_H_18_N_4_O_2_: %C 69.35, %H 5.24, %N 16.17; found: %C 68.64, %H 5.13, %N 16.13. mp 199–201 °C (decomp). MS (ESI) *m*/*z* 347 [C_20_H_18_N_4_O_2_+H]^+^. FTIR (ATR) ν (cm^−1^): 3337 (ν-NH_indole_); 3214 (ν-NH_Py_); 3062 (ν-CH_ph_); 2964 (ν_as_-CH_3_); 2873 (ν_s_-CH_3_); 1611 (ν-C=N); 1597 (ν-C=C). Raman (ATR) ν (cm^−1^): 1608 (ν-C=N); 1592 (ν-C=C_indole_). UV–vis (DMSO, λ_max_ nm) (log ε): λ_1_ 328 (4.38). ^1^H NMR (300 MHz, DMSO-*d_6_*, δ in ppm) 3.85 (s, 3H, H-14); 3.89 (s, 3H, H-13); 6.80 (s, 1H, H-12); 7.129 (m, 3H, H-9, H-10, H-11); 7.47 (m, 2H, H-8, H-7); 7.59 (s, 1H, H-6); 7.81 (s, 1H, H-5); 8.17 (d, 1H, H-4, ^3^*J* = 6.09); 8.91 (s, 1H, H-3); 11.45 (s, 1H, H-2); 12.80 (s, 1H, H-1). ^13^C NMR (75 MHz, DMSO-*d_6_*, δ in ppm): 55.88 (C-14); 56.11 (C-13); 92.26 (C-12); 105.80 (C-22); 109.45 (C-6); 111.86 (C-8); 112.43 (C-11); 119.88 (C-4); 120.31 (C-10); 122.31 (C-9); 123.58 (C-5); 124.12 (C-4); 124.95 (C-21); 129.85 (C-20); 136.84 (C-19); 139.09 (C-18); 149.54 (C-17); 152.09 (C-16); 159.57 (C-3); 160.14 (C-15).

### 4.12. Synthesis of [Ru(p-Cymene)(pyrazole-imine)Cl_2_] Complexes (***Ru3***–***Ru6***)

A mixture of 57.8 mmol of the corresponding pyrazole-imine (**3**–**6**) and 28.9 mmol of [Ru(*p*-cymene)Cl_2_]_2_ in 5 mL of dry acetone was stirred at room temperature for 1 h under an inert atmosphere. The solids were washed with cold diethylether and dried under vacuum.

*[Ru(p-cymene)(***3***)Cl_2_] (***Ru3***):* Orange solid, 39 mg, yield: 78%. mp 264 °C (decomp). MS (ESI) *m*/*z* 614 [C_28_H_28_Cl_2_N_4_ORu+6H]^+^. FTIR (ATR) ν (cm^−1^): 3401(ν-NH_indole_); 3252 (ν-NH_Py_); 3127 (ν-CH_ph_); 2943 (ν_as_-CH_3_); 2833 (ν_s_-CH_3_); 1621 (ν-C=N); 1566 (ν-C=C); 384 (ν-Ru-N); 473 (ν-Ru-N); 385 (ν-Ru-C); 292 (ν_as_-Ru-Cl); 242 (ν_s_-Ru-Cl). Raman (ATR) ν (cm^−1^): 1611 (ν-C=N); 1574 (ν-C=C_indole_); 1520 (ν-C=C*_p_*_-cym_); 465 (ν-Ru-N); 359 (ν-Ru-C); 286 (ν_s_-Ru-Cl); 230 (ν_as_-Ru-Cl). UV–vis (DMSO, λ_max_ nm) (log ε): λ_1_ 297 (4.12); λ_2_ 344 (4.02); λ_3_ 447 (2.41). ^1^H NMR (400 MHz, DMSO-*d_6_*, δ in ppm) 1.19 (d, 6H, H-27, ^3^*J* = 6.9); 2.09 (s, 3H, H-26); 2.84 (hept, 1H, H-25, ^3^*J* = 6.9); 5.77 (d, 2H, H-24, ^3^*J* = 6.3); 5.82 (d, 2H, H-23, ^3^*J* = 6.3); 6.95 (s, 1H, H-12); 6.99 (t, 1H, H-10, ^3^*J* = 7.4); 7.02 (t, 1H, H-9, ^3^*J* = 7.4); 7.16 (*t*, 1H, H-11, ^3^*J* = 7.4); 7.21 (t, H-7, ^3^*J* = 7.4); 7.42 (d, 1H, H-30, ^3^*J* = 7.4); 7.49 (d, 1H, H-8, ^3^*J* = 7.9); 7.66 (dd, 1H, H-6, ^3^*J* = 7.6, ^4^*J* = 1.6); 7.84 (s, 1H, H-5); 7.99 (d, 1H, H-4); 9.23 (s, 1H, H-3); 11.47 (s, 1H, H-2); 12.99 (s, 1H, H-1); 13.29 (s, 1H, OH). ^13^C NMR (75 MHz, DMSO-*d_6_*, δ in ppm): 18.33 (C-26); 21.97 (C-27); 30.45 (C-25); 85.98 (C-23); 86.83 (C-24); 92.19 (C-12); 100.57 (C-29); 106.89 (C-28); 112.46 (C-7); 117.09 (C-30); 119.70 (C-11); 119.95 (C-4); 120.31 (C-10); 122.38 (C-9); 123.89 (C-5); 124.89 (C-20 and C-21); 132.71 (C-6); 133.42 (C-8); 136.85 (C-19); 137.33 (C-18); 160.70 (C-15); 162.72 (C-16); 162.80 (C-3).

*[Ru(p-cymene)(***4***)Cl_2_] (***Ru4***):* Orange solid, 30 mg, yield: 79%. mp 223 °C(decomp). MS (ESI) *m*/*z* 658 [C_30_H_32_Cl_2_N_4_O_2_Ru+H]^+^. Anal. Calc. For C_30_H_32_Cl_2_N_4_O_2_Ru: %C 55.52, %H 4.94, %N 8.59; found: %C 54.47, %H 5.10, %N 8.49. FTIR (ATR) ν (cm^−1^): 3335 (ν-NH_indole_); 3152 (ν-NH_Py_); 3000 (ν-CH_ph_); 2956 (ν_as_-CH_3_); 2869 (ν_s_-CH_3_); 1621 (ν-C=N); 1504 (ν-C=C); 427 (ν-Ru-N); 384 (ν-Ru-C); 323 (ν_as_-Ru-Cl); 292 (ν_s_-Ru-Cl). Raman (ATR) ν (cm^−1^): 1617 (ν-C=N); 1584 (ν-C=C_indole_);1520 (ν-C=C*_p_*_-cym_); 446 (ν-Ru-N); 343 (ν-Ru-C); 317 (ν_s_-Ru-Cl); 269 (ν_as_-Ru-Cl). UV–vis (DMSO, λ_max_ nm) (log ε): λ_1_ 286 (4.25); λ_2_ 341(4.27); λ_3_ 447 (2.60). ^1^H NMR (300 MHz, DMSO-*d_6_*, δ in ppm) 1.19 (d, 6H, H-27, ^3^*J* = 6.94); 2.09 (s, 3H, H-26); 2.81 (hept, 1H, H-25, ^3^*J* = 6.97); 3.87 (s, 3H, H-14); 3.93 (s, 3H, H-13); 5.77 (d, 2H, H-24, ^3^*J* = 6.39); 5.82 (d, 2H, H-23, ^3^*J* = 6.42); 6.657 (s, 1H, H-12); 6.67 (m, 3H, H-10, H-12; H-11); 7.15 (m, 2H, H-9, H-8); 7.45 (d, 1H, H-7, ^3^*J* = 7.77); 7.79 (s, 1H, H-6); 7.96 (s, 1H, H-5); 8.02 (m, 2H, H-5; H-4); 9.11 (s, 1H, H-3); 11.41 (s, 1H, H-2); 12.73 (s, 1H, H-1). ^13^C NMR (75 MHz, DMSO-*d_6_*, δ in ppm): 18.84 (C-26); 21.96 (C-27); 30.45 (C-25); 56.03 (C-13); 56.33 (C-14); 85.98 (C-23); 86.83 (C-24); 92.98 (C-12); 98.62 (C-11); 100.58 (C-29); 106.85 (C-28); 107.07 (C-22); 112.26 (C-8); 117.72 (C-7); 117.79 (C-20); 119.75 (C-10); 120.10 (C-21); 120.22 (C-5); 122.15 (C-9); 123.72 (C-6); 128.39 (C-4); 136.82 (C-19); 154.19 (C-18); 154.29 (C-17); 154.44 (C-3); 161.229 (C-16); 164.083 (C-15).

*[Ru(p-cymene)(***5***)Cl_2_] (***Ru5***)*: Orange solid, 35 mg, yield: 79%. mp 225 °C (decomp). MS (ESI) *m*/*z* 658 [C_30_H_32_Cl_2_N_4_O_2_Ru+H]^+^. Anal. Calc. For C_30_H_32_Cl_2_N_4_O_2_Ru: %C 55.52, %H 4.94, %N 8.59; found: %C 54.60, %H 4.80, %N 8.44. FTIR (ATR) ν (cm^−1^): 3259 (ν-NH_indole_); 3216 (ν-NH_Py_); 3071 (ν-NH_indole_); 3029 (ν-CH_ph_); 2961 (ν_as_-CH_3_); 2833 (ν_s_-CH_3_); 1616 (ν-C=N); 1511 (ν-C=C); 416 (ν-Ru-N); 379 (ν-Ru-C); 289 (ν_as_-Ru-Cl); 235 (νs-Ru-Cl). Raman (ATR) ν (cm^−1^): 1609 (ν-C=N); 1530 (ν-C=C*_p_*_-cym_); 445 (ν-Ru-N); 350 (ν-Ru-C); 312 (ν_s_-Ru-Cl); 277 (ν_as_-Ru-Cl). UV–vis (DMSO, λ_max_ nm) (log ε): λ_1_ 283 (4.26); λ_2_ 365 (3.99); λ_3_ 477 (2.50). ^1^H NMR (300 MHz, DMSO-*d_6_*, δ in ppm) 1.19 (d, 6H, H-27, ^3^*J* = 6.94); 2.09 (s, 3H, H-26); 2.83 (hept, 1H, H-25, ^3^*J* = 6.92); 3.79 (s, 3H, H-14); 3.89 (s, 3H, H-13); 5.77 (d, 2H, H-24, ^3^*J* = 6.38); 5.82 (d, 2H, H-23, ^3^*J* = 6.39); 6.75 (s, 1H, H-12); 7.12 (m, 4H, H-7, H-9,H-10,H-11); 7.15 (m, 2H, H-10, H-7); 7.46 (bs, 1H, H-8); 7.58 (s, 1H, H-6); 7.82 (s, 1H, H-5); 7.95 (bs, 1H, H-4); 9.22 (s, 1H, H-3); 11.46 (s, 1H, H-2); 12.85 (s, 1H, H-1). ^13^C NMR (75 MHz, DMSO-*d_6_*, δ in ppm): 18.34 (C-26); 21.95 (C-27); 30.45 (C-25); 55.95 (C-14); 56.77 (C-13); 85.97 (C-23); 86.83 (C-24); 92.92 (C-12); 100.57 (C-29); 106.86 (C-22); 110.07 (C-6); 111.94 (C-28); 112.30 (C-8); 114.12 (C-8); 119.92 (C-10); 122.23 (C-9); 123.80 (C-5); 124.94 (C-20); 125.05 (C-21); 136.82 (C-19); 139.78 (C-18); 153.71 (C-17); 154.28 (C-16); 154.46 (C-3); 154.65 (C-15).

*[Ru(p-cymene)(***6***)Cl_2_] (***Ru6***):* Orange solid, 33 mg, yield: 89%. mp 246 °C (decomp). MS (ESI) m/z 658 [C_30_H_32_Cl_2_N_4_O_2_Ru+H]^+^. Anal. Calc. For C_30_H_32_Cl_2_N_4_O_2_Ru: %C 55.52, %H 4.94, %N 8.59; found: %C 54.47, %H 4,87, %N 8.49. FTIR (ATR) ν (cm^−1^): 3551 (ν-NH_indole_); 3282 (ν-NH_Py_); 3058 (ν-CH_ph_); 2929 (ν_as_-CH_3_); 2837 (ν_s_-CH_3_); 1627 (ν-C=N); 1596 (ν-C=C); 395 (ν-Ru-N); 385 (ν-Ru-C); 318 (ν_as_-Ru-Cl); 285 (ν_s_-Ru-Cl). Raman (ATR) ν (cm^−1^): 1601 (ν-C=N); 1582 (ν-C=C_indole_); 1519 (ν-C=C_p-cym_); 450 (ν-Ru-N); 384 (ν-Ru-C); 310 (ν_s_-Ru-Cl); 262 (ν_as_-Ru-Cl). UV–vis (DMSO, λ_max_ nm) (log ε): λ_1_ 329 (4.12); λ_2_ 477 (2.20). ^1^H NMR (300 MHz, DMSO-d_6_, δ in ppm) 1.19 (d, 6H, H-27, ^3^*J* = 6.94); 2.091 (s, 3H, H-26); 2.84 (hept, 1H, H-25, ^3^*J* = 6.92); 3.85 (s, 3H, H-14); 3.87 (s, 3H, H-13); 5.78 (d, 2H, H-24, ^3^*J* = 6.36); 5.82 (d, 2H, H-23, ^3^*J* = 6.39); 6.78 (s, 1H, H-12); 7.12 (m, 3H; H-9, H-10; H-11); 7.46 (m, 2H, H-8, H-7); 7.58 (s, 1H, H-6); 7.79 (s, 1H, H-5); 7.97 (bs, 1H, H-4); 8.89 (s, 1H, H-3); 11.43 (s, 1H, H-2); 12.79 (s, 1H, H-1). ^13^C NMR (75 MHz, DMSO-d_6_, δ in ppm): 18.35 (C-26); 21.97 (C-27); 30.45 (C-25); 55.89 (C-14); 56.01 (C-13); 85.98 (C-23); 86.84 (C-24); 92.16 (C-12); 100.55 (C-29); 106.46 (C-22, C-28); 109.46 (C-6); 112.37 (C-8); 111.88 (C-11); 120.08 (C-10); 120.23 (C-4); 122.26 (C-9); 123.58 (C-5); 125.03 (C-21); 129.63 (C-18); 129.77 (C-20); 136.84 (C-19); 139.09 (C-18); 149.55 (C-17); 152.18 (C-16); 159.63 (C-3); 159.67 (C-15).

## 5. Conclusions

Four new Schiff bases derived from pyrazole-indole were synthesized and successfully coordinated to a [Ru^II^(η^6^-*p*-cymene)Cl_2_] fragment. The free ligands were inactive, whereas all four ruthenium complexes exhibited bactericidal activity. In particular, **Ru4** killed Gram-positive bacteria, including *E. faecalis* and *S. aureus*, at 62.5 µg/mL. The **Ru3** and **Ru6** derivatives killed *S. dysenteriae* and *S. aureus* at 62.5 µg/mL. Molecular docking of **Ru4** and **Ru6** showed more favorable binding affinities at the active site of *S. aureus* PBP2a, suggesting a possible mechanism of action. These compounds exhibited hydrogen-bonding interactions with residues in the active site. Furthermore, the docking results were consistent with the in vitro antibacterial activity, as **Ru4** and **Ru6** showed the lowest MBC values against *S. aureus*. As such, the **Ru4** and **Ru6** complexes represent the most promising candidates as PBP2a inhibitors and highlight the potential of ruthenium compounds for developing new antibacterial agents against resistant bacterial pathogens.

## Figures and Tables

**Table 1:** Crystallographic data for compounds **3** and **4**.

Compound	3	4
Chemical formula	C_18_H_14_N_4_O	C_20_H_18_N_4_O_2_
FW	302.12	364.12
Crystal system, space group	Monoclinic, C2	Monoclinic, P2_1_/n
Temperature (K)	100	150
*a* (Å)	26.7587 (7)	15.7360 (7)
*b* (Å)	5.0903 (2)	5.3614 (2)
*c* (Å)	23.1689 (7)	23.3582 (9)
(*α*) Å	90	90
*β* (Å)	94.254	109.150 (2)
(*ϒ*) Å	90	90
*V* (Å^3^)	3147.14 (18)	1861.618 (13)
*Z*	8	4
ρ_cal_ (mg × m^3^)	1.352	1.236
Abs.Coeff. (mm^−1^)	0.092	0.668
F (000)	1.344.0	728.0
Range	2.645 to 28.699	2.990 to 74.621
Crystal size (mm)	0.02/0.07/0.29	1.101/−0.779/0.159
Reflections collected/unique	8118/	3820/
R_int_		
Completeness (%)	99.8	96.4
Data/restraints/parameters	28787/1/441	3687/0/238
GoF on F^2^	1.037	1.888
(sin θ/***λ***) _max_ (Å^−1^)	28.699	74.621
*R1*[F^2^ > 2σ (*F^2^*)]	0.0409	0.1460
w *R2* (*F^2^*)	0.0996	0.4543
Final R-index	0.0409	0.1460

**Table 2:** Antibacterial activity of compounds in the evaluated isolates. Dilutions were prepared to obtain the concentrations to be evaluated: 250 μg/mL, 62.5 μg/mL, 15.6 μg/mL, 3.9 μg/mL, 0.97 μg/mL, 0.24 μg/mL, and 0.06 μg/mL, and the dilutions were incubated at 37 °C for 18 to 20 h. Orange color highlights compounds with antibacterial activity [24].

	*Enterococcus* *faecalis ATCC* *29212*		*Staphylococcus* *aureus*		*Klebsiella* *pneumoniae*		*Acinetobacter* *baumannii*		*Pseudomonas* *aeruginosa*		*Enterobacter* *cloacae ATCC* *13047*		*Escherichia* *coli*		*Shigella* *dysenteriae*		*Average* *MIC*
	17		4R		H-9871		S-300		S-171		13047		U-13824		20		
	MIC	MBC	MIC	MBC	MIC	MBC	MIC	MBC	MIC	MBC	MIC	MBC	MIC	MBC	MIC	MBC	
**3**	>250	>250	>250	>250	>250	>250	>250	>250	>250	>250	>250	>250	>250	>250	>250	>250	>250
**4**	>250	>250	>250	>250	>250	>250	>250	>250	>250	>250	>250	>250	>250	>250	>250	>250	>250
**5**	>250	>250	>250	>250	>250	>250	>250	>250	>250	>250	>250	>250	>250	>250	>250	>250	>250
**6**	>250	>250	>250	>250	>250	>250	>250	>250	>250	>250	>250	>250	>250	>250	>250	>250	>250
**Ru3**	250	>250	15.60	>250	>250	>250	>250	>250	>250	>250	>250	>250	>250	>250	62.5	62.5	>197.26
**Ru4**	62.5	62.5	15.60	62.5	>250	>250	>250	>250	>250	>250	>250	>250	>250	>250	250	250	>197.26
**Ru5**	>250	>250	62.5	>250	>250	>250	>250	>250	>250	>250	>250	>250	>250	>250	>250	>250	>226.52
**Ru6**	>250	>250	62.5	62.5	>250	>250	250	>250	>250	>250	250	>250	>250	>250	250	250	>226.52

**Table 3:** Ligand affinity energies and control drugs with PBP2a.

Compound	Affinity (Kcal/mol)
**Ru3**	−6.26
**Ru4**	−7.05
**Ru5**	−6.31
**Ru6**	−7.27
Ceftaroline	−6.42
Gentamicin	−5.11
Trimethoprim	−5.10

## Data Availability

The data presented in this study are available in the article and Supplementary Materials.

## Supplementary Materials

The following supporting information can be downloaded at: https://www.mdpi.com/article/10.3390/antibiotics15070675/s1. ^1^H-NMR, ^13^C-NMR, UV-vis, MS, IR, and Raman spectra of **3**–**6** and **Ru3**–**Ru6** (Figures S2–S69); UV-vis absorption spectra for the stability assay of **Ru-3**–**Ru-6** (Figures S1–S70); Selected bond distances and angles for compounds **3** and **4** (Table S1); Experimental and theoretical IR, UV-Vis, and Raman, ^1^H- and ^13^C-NMR spectroscopic data (Tables S2–S6).
