# Supplementary material for: Activity Against ESKAPE Bacterial Pathogens of Pyrazole-Indol-Ruthenium(II) Complexes
Source: Antibiotics (Basel). 2026 Jul 9;15(7):675. doi: 10.3390/antibiotics15070675 (PMC13405323; doi:10.3390/antibiotics15070675)
Supplement: Supplementary file 1 [file antibiotics-15-00675-s001.zip › antibiotics-4412620-supplementary.pdf]

Supporting Information

Activity Against ESKAPE Bacterial Pathogens of Pyrazole-Indol-Ruthenium(II) Complexes

Yahaira Cuenú-Ibargüen [1,2], Andrés Restrepo-Acevedo [1], Juan Felipe Zambrano-Bedoya [1], Isabel Murillo-Rodriguez [1], Carlos Felipe Mejía [1], Sandra Fabiola Alzate-Walteros [2], Gladymar Guadalupe Valenzuela-Ramirez [3], Gilmar López-Armenta [3], Federico del Rio-Portilla [1], Jesús Ricardo Parra-Unda [3,*], Fernando Cuenú-Cabezas [2,*] and Ronan Le Lagadec [1,*]

[1] Instituto de Química, Universidad Nacional Autónoma de México, Circuito Exterior s/n, Ciudad Universitaria, Ciudad de México 04510, Mexico; yahaira.cuenui@uqvirtual.edu.co (Y.C.-I.); acrestrepoa@gmail.com (A.R.-A.); jufezabe@comunidad.unam.mx (J.F.Z.-B.); murillo.isabel5@gmail.com (I.M.-R.); cfmejiag@comunidad.unam.mx (C.F.M.); federico.delrio@iquimica.unam.mx (F.d.R.-P.)

[2] Programa de Química, Facultad de Ciencias Básicas y Tecnologías, Universidad del Quindío, Carrera 15 Calle 12 Norte, Armenia 630004, Quindío, Colombia; sfalzate@uniquindio.edu.co

[3] Unidad de Investigaciones en Salud Pública "Dra Kaethe Willms", Facultad de Ciencias Químico Biológicas, Universidad Autónoma de Sinaloa, Ciudad Universitaria, Culiacán 80013, Sinaloa, Mexico; gladymarvzla.fcqb@uas.edu.mx (G.G.V.-R.); gilmar.fcqb@uas.edu.mx (G.L.-A.)

* Correspondence: ricardoparraund@uas.edu.mx (J.R.P.-U.); fercuenu@uniquindio.edu.co (F.C.-C.); ronan@unam.mx (R.L.L.)

Figure S1. Numbering scheme for NMR assignment of pyrazole-imines **3 – 6** and ruthenium complexes **Ru3 – Ru6**

Figure S2. GM-MS (IE) spectrum of **3**

Figure S3. FT-IR(ATR) spectrum of **3**

Figure S4. RAMAN(ATR) spectrum of **3**

Figure S5. FT-IR (ATR) and Raman (ATR) theoretical spectrums of **3**

Figure S6. IR Correlation diagram of compound **3**

Figure S7. Raman Correlation diagram of compound **3**

Figure S8. ^{1}H-NMR spectrum of **3**

Figure S9. ^{13}C-NMR spectrum of **3**

Figure S10. GM-MS (ESI) spectrum of **4**

Figure S11. FT-IR (ATR) spectrum of **4**

Figure S12. Raman (ATR) spectrum of **4**

Figure S13. FT-IR (ATR) and Raman (ATR) theoretical spectrums of **4**

Figure S14. IR Correlation diagram of compound **4**

Figure S15. Raman Correlation diagram of compound **4**

Figure S16. ^{1}H-NMR spectrum of **4**

Figure S17. ^{13}C-NMR spectrum of **4**

Figure S18. GM-MS (ESI) spectrum of **5**

Figure S19. FT-IR (ATR) spectrum of **5**

Figure S20. Raman (ATR) spectrum of **5**

Figure S21. FT-IR (ATR) and Raman (ATR) theoretical spectrums of **5**

Figure S22. IR Correlation diagram of compound **5**

Figure S23. Raman Correlation diagram of compound **5**

Figure S24. ^{1}H-NMR spectrum of **5**

Figure S25. ^{13}C-NMR spectrum of **5**

Figure S26. GM-MS (ESI) spectrum of **6**

Figure S27. FT-IR (ATR) spectrum of **6**

Figure S28: Raman (ATR) spectrum of **6**

Figure S29. FT-IR (ATR) and Raman (ATR) theoretical spectrums of **6**

Figure S30. IR Correlation diagram of compound **6**

Figure S31. Raman Correlation diagram of compound **6**

Figure S32. ^{1}H-NMR spectrum of **6**

Figure S33. ^{13}C-NMR spectrum of **6**

Figure S34. GM-MS (ESI) spectrum of **Ru3**

Figure S35. FT-IR (ATR) spectrum of **Ru3**

Figure S36. Raman (ATR) spectrum of **Ru3**

Figure S37. FT-IR (ATR) and Raman (ATR) theoretical spectrums of Ru**3**

Figure S38. IR Correlation diagram of compound **Ru3**

Figure S39. Raman Correlation diagram of compound **Ru3**

Figure S40. ^{1}H-NMR spectrum of **Ru3**

Figure S41. ^{13}C-NMR spectrum of **Ru3**

Figure S42. GM-MS (ESI) spectrum of **Ru4**

Figure S43. FT-IR(ATR) spectrum of **Ru4**

Figure S44. Raman (ATR) spectrum of **Ru4**

Figure S45. FT-IR (ATR) and Raman (ATR) theoretical spectrums of **Ru4**

Figure S46. IR Correlation diagram of compound **Ru4**

Figure S47. Raman Correlation diagram of compound **Ru4**

Figure S48. ^{1}H-NMR spectrum of **Ru4**

Figure S49. ^{13}C-NMR spectrum of **Ru4**

Figure S50. GM-MS (ESI) spectrum of **Ru5**

Figure S51. FT-IR (ATR) spectrum of **Ru5**

Figure S52. RAMAN (ATR) spectrum of **Ru5**

Figure S53. FT-IR (ATR) and Raman (ATR) theoretical spectrums of **Ru5**

Figure S54. IR Correlation diagram of compound **Ru5**

Figure S55. IR Correlation diagram of compound **Ru5**

Figure S56. ^{1}H-NMR spectrum of **Ru5**

Figure S57. ^{13}C-NMR spectrum of **Ru5**

Figure S58. GM-MS (ESI) spectrum of **Ru6**

Figure S59: FT-IR (ATR) spectrum of **Ru6**

Figure S60. RAMAN (ATR) spectrum of **Ru6**

Figure S61. FT-IR (ATR) and Raman (ATR) theoretical spectrums of **Ru6**

Figure S62. IR Correlation diagram of compound **Ru6**

Figure S63. Raman Correlation diagram of compound **Ru6**

Figure S64. ^{1}H-NMR spectrum of **Ru6**

Figure S65. ^{13}C-NMR spectrum of **Ru6**

Figure S66. UV–vis spectra of **Ru3** (**A**), **Ru4** (**B**), **Ru5** (**C**), and **Ru6** (**D**) at 10 μM in a PBS/DMSO (0.1%) solution for 24 h at 25 °C.

Figure S67. Experimental UV–vis spectra of **3-6**

Figure S68. Experimental UV–vis spectra of **Ru3-Ru6**

Figure S69. Theorical UV–vis spectra of **3-6**

Figure S70. Theorical UV–vis spectra of **Ru3-Ru6**

Table S1. Selected bond angles (°) and lengths (Å) of compounds **3** and **4**.

Table S2. Experimental and theoretical vibrational frequencies (cm^{-1}) in the infrared region of ligands and ruthenium complexes.

Table S3. Experimental and theoretical vibrational frequencies (cm^{-1}) in the RAMAN of ligands and ruthenium complexes

Table S4. Experimental electronic absorption wavelengths and important Bases of Schiff and their complexes.

Table S5. HOMO - LUMO energies and calculated global reactivity parameters of Schiff and their complexes calculated via B3LYP/6-311Gþþ (d, p) method.

Table S6. ^{1}H-NMR and ^{13}C-NMR experimental shifts for the Schiff bases and their complexes.

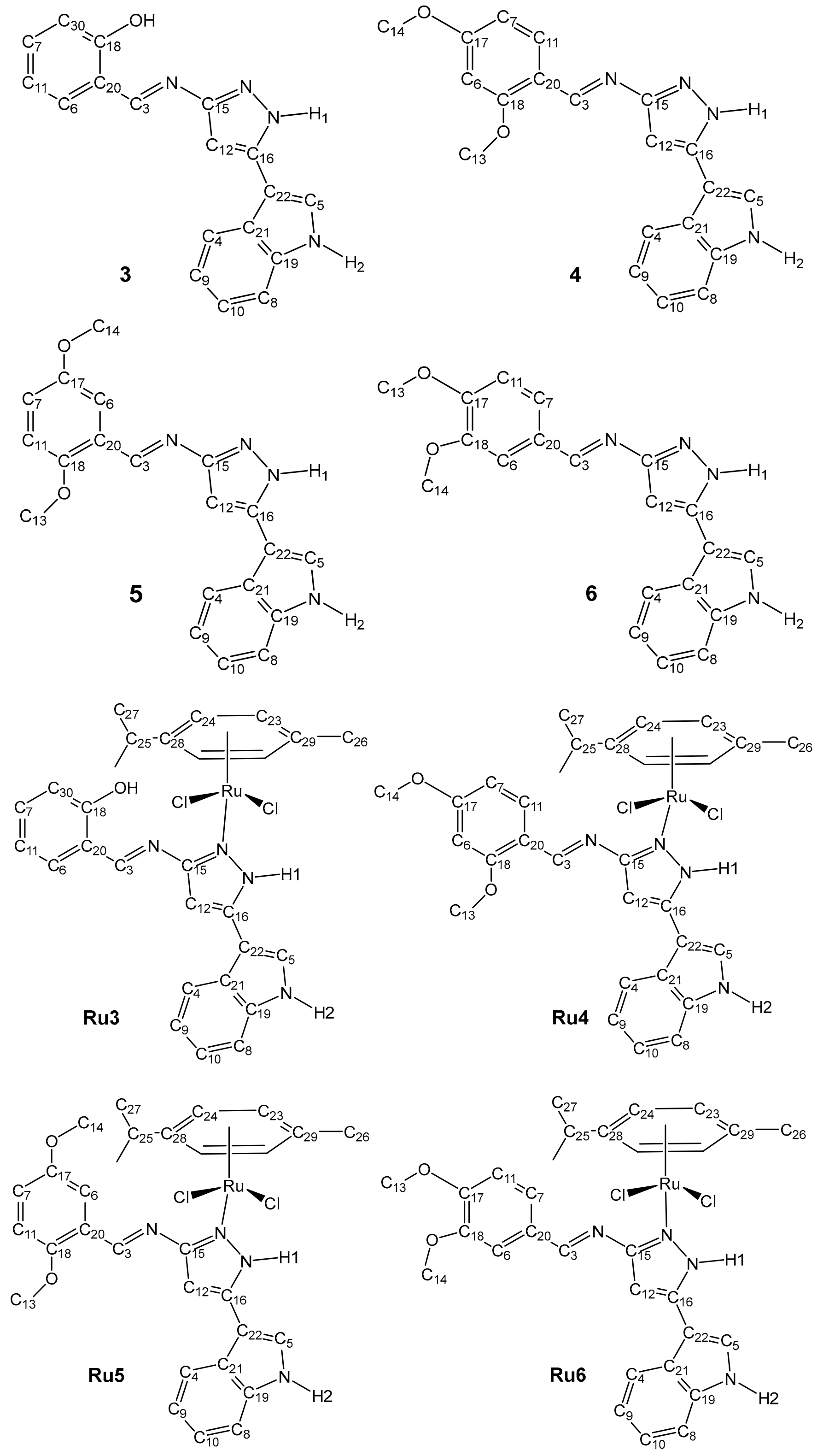

Figure S1. Numbering scheme for NMR assignment of pyrazole-imines **3 – 6** and ruthenium complexes **Ru3 – Ru6**.

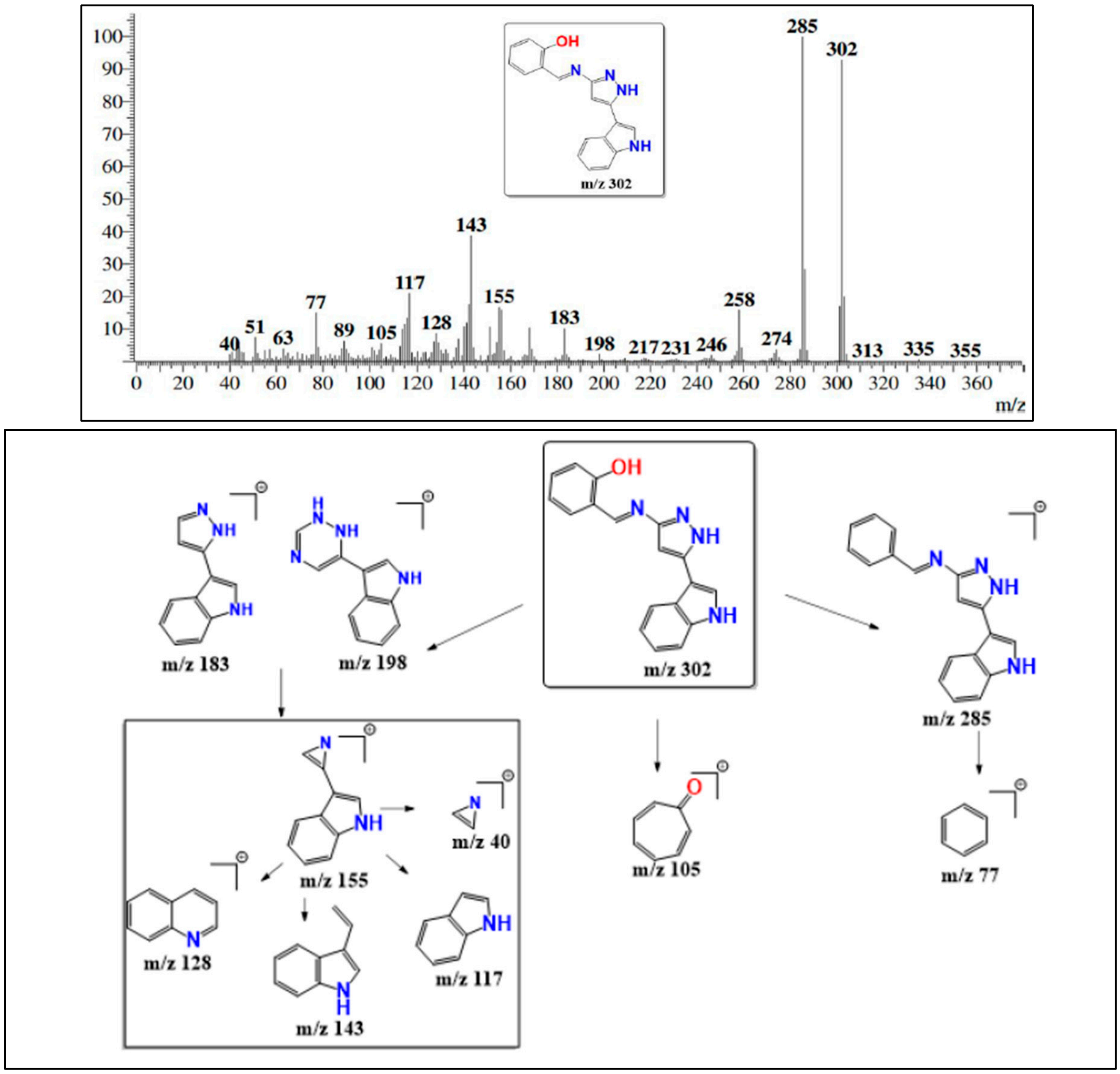

Figure S2. GM-MS (IE) spectrum of **3**.

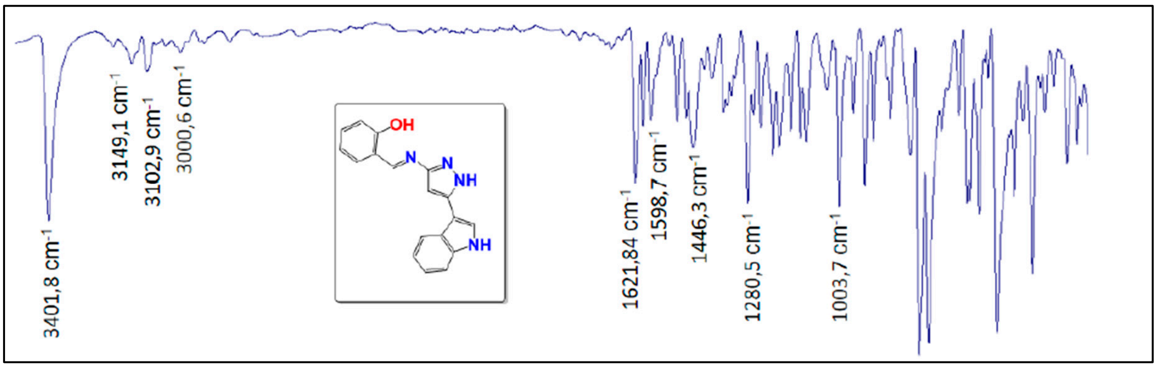

Figure S3. FT-IR(ATR) spectrum of **3**.

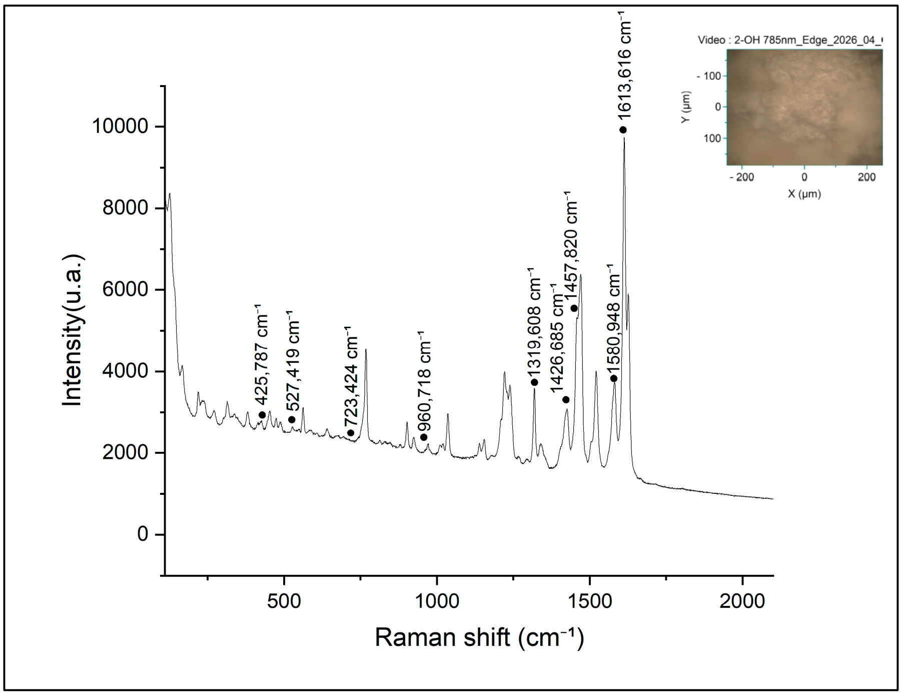

Figure S4. Raman(ATR) spectrum of **3**.

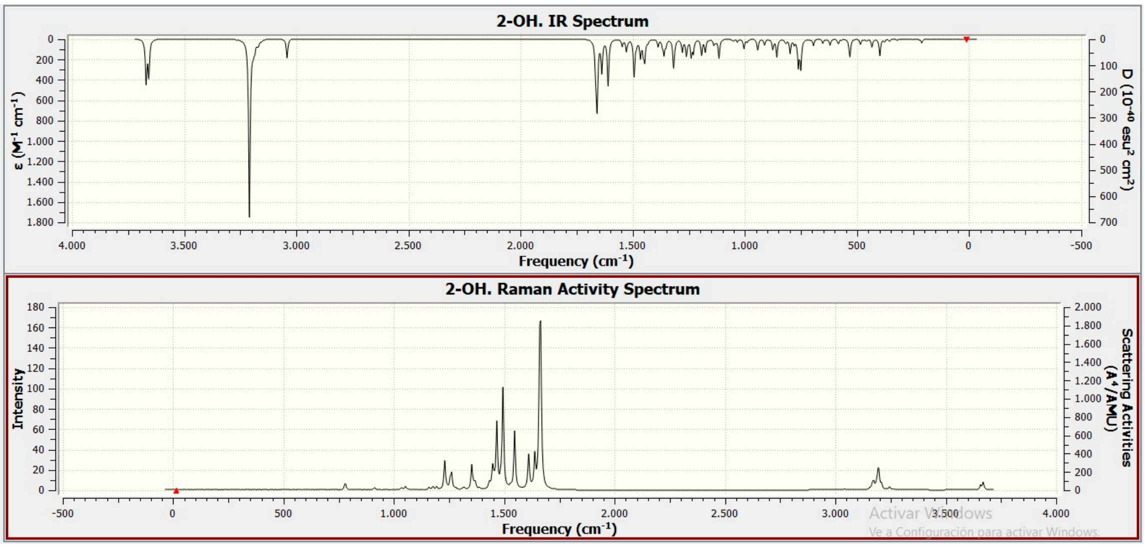

Figure S5. FT-IR (ATR) and Raman (ATR) theoretical spectrums of **3**.

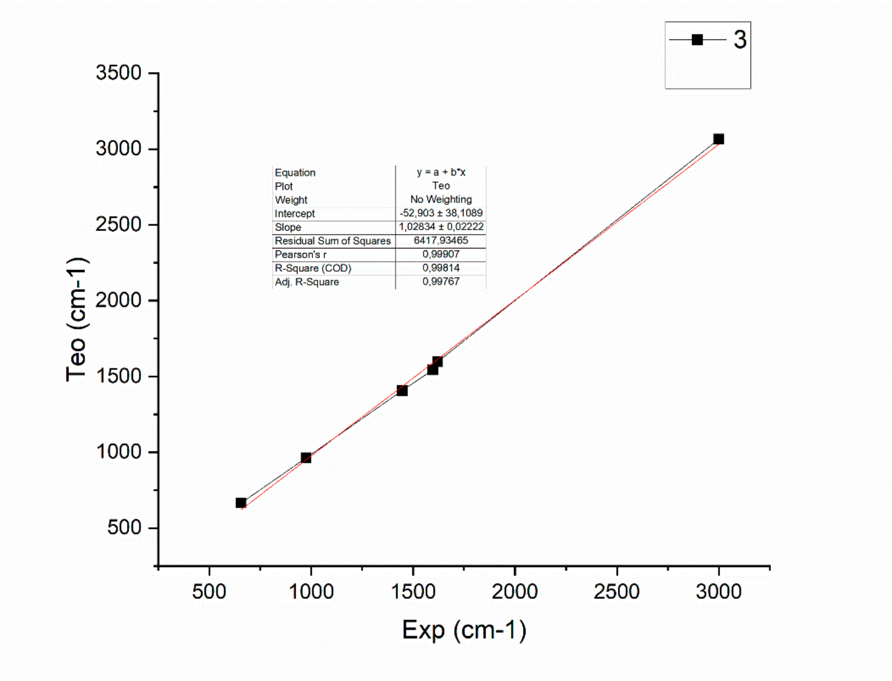

Figure S6. IR Correlation diagram of compound **3.**

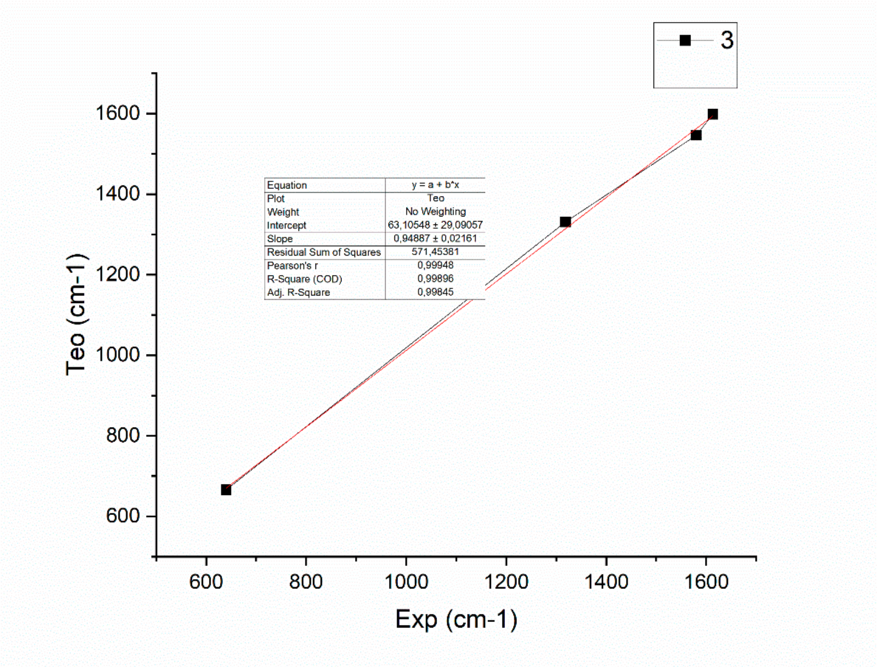

Figure S7. Raman Correlation diagram of compound **3.**

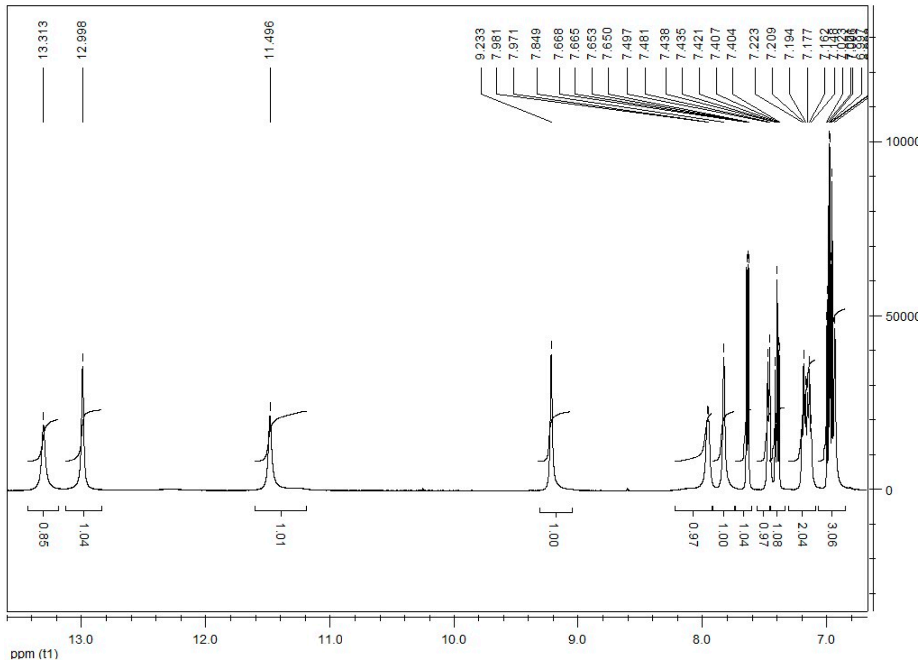

Figure S8. ^{1}H-NMR spectrum of **3**.

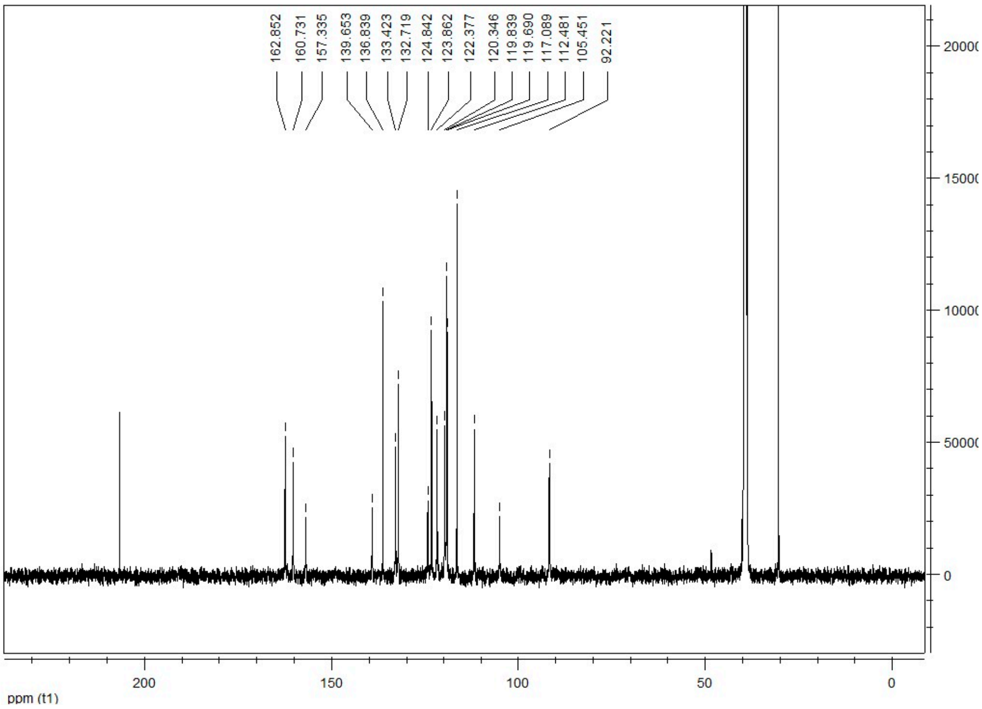

Figure S9. ^{13}C-NMR spectrum of **3**.

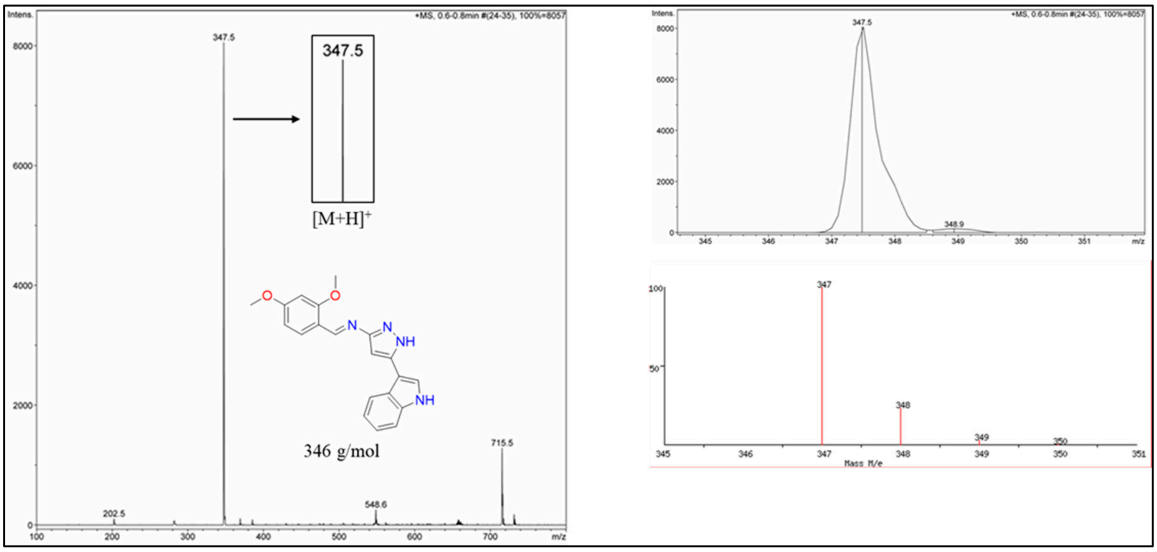

Figure S10. GM-MS (ESI) spectrum **4**.

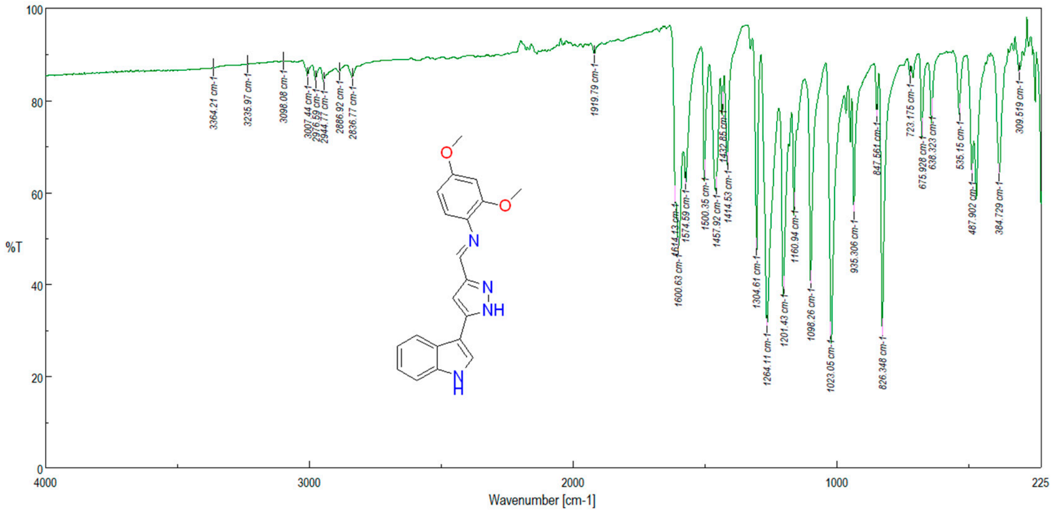

Figure S11. FT-IR (ATR) spectrum of **4**.

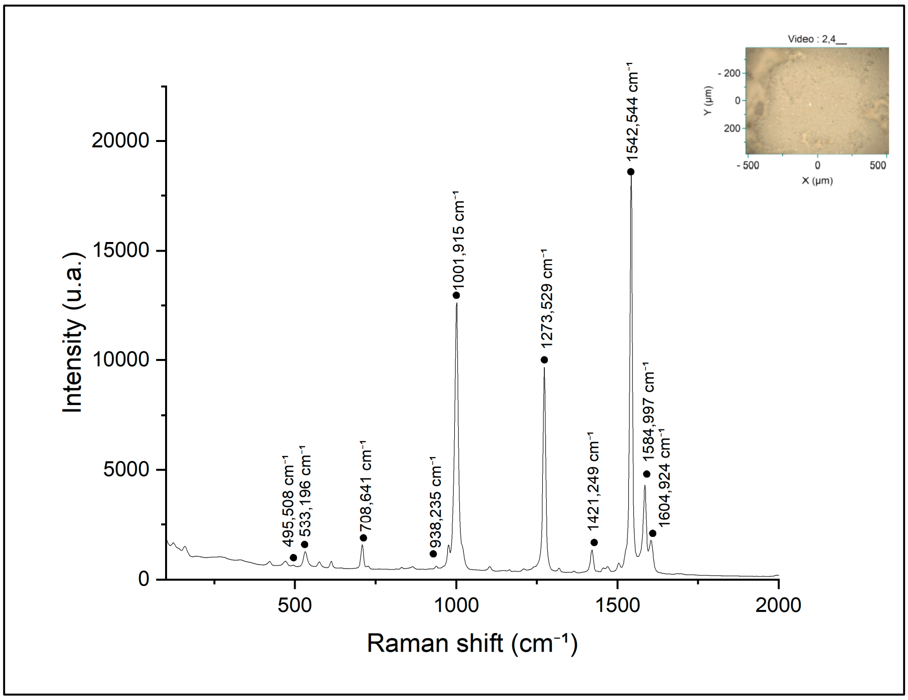

Figure S12. Raman (ATR) spectrum of **4**.

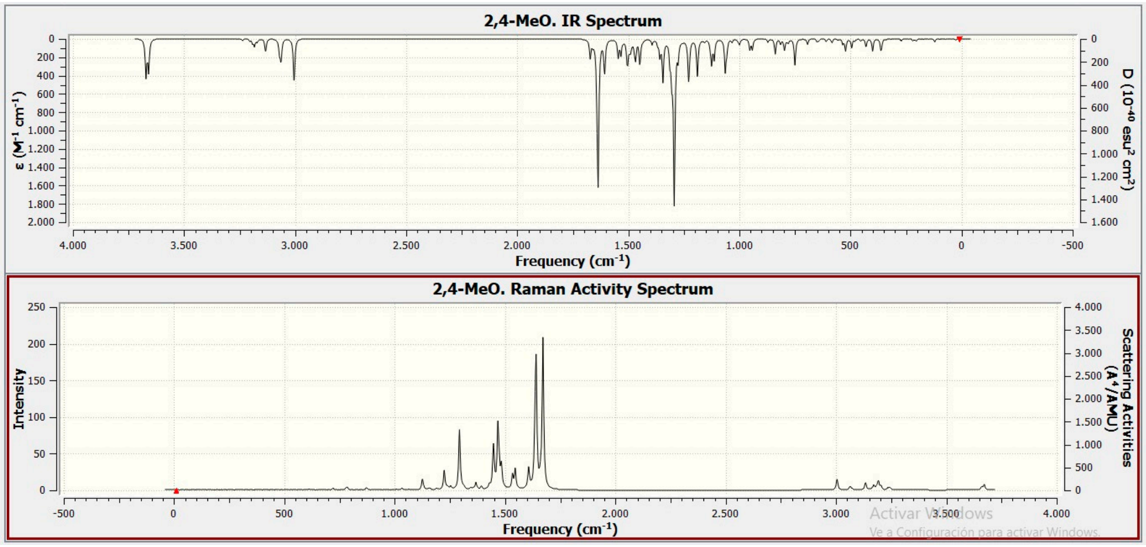

Figure S13. FT-IR (ATR) and Raman (ATR) theoretical spectrums of **4**.

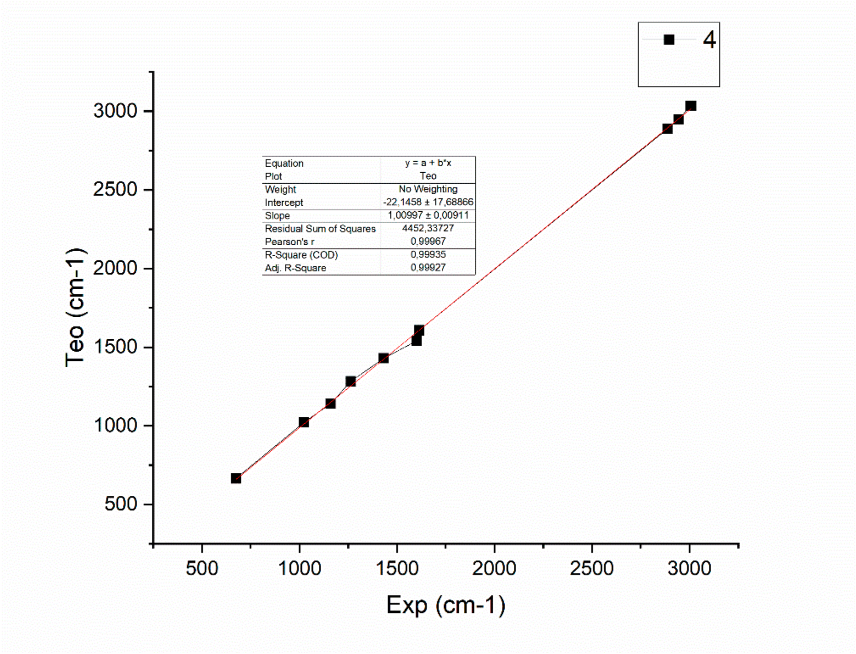

Figure S14. IR Correlation diagram of compound **4.**

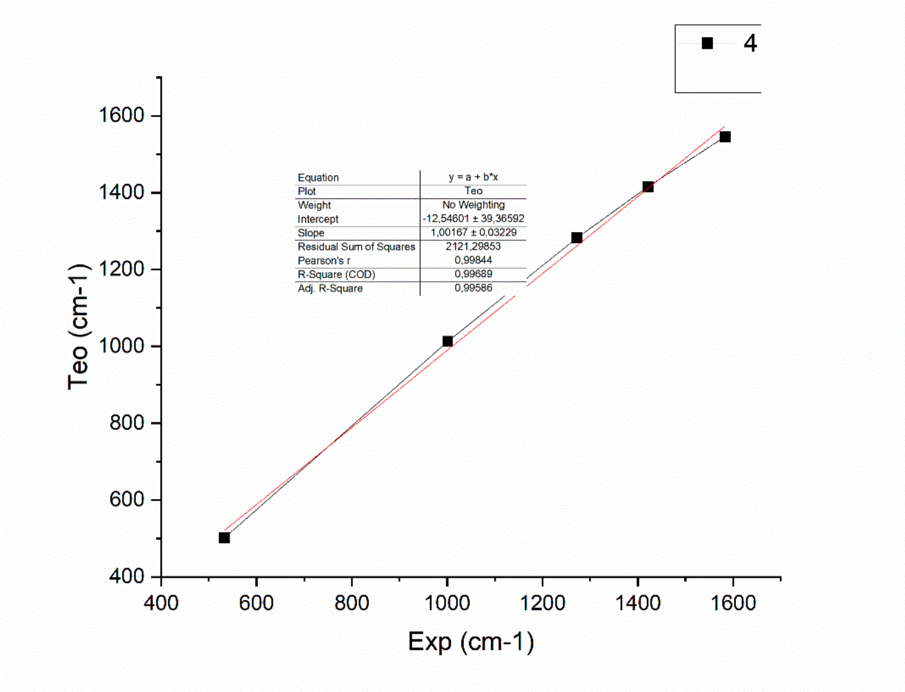

Figure S15. Raman Correlation diagram of compound **4.**

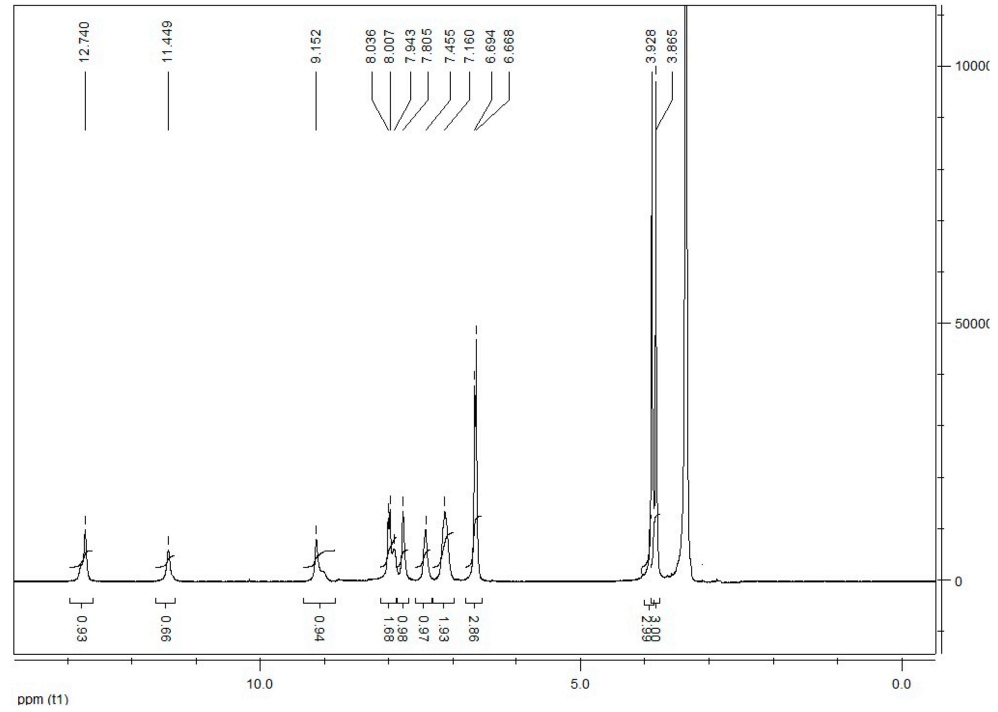

Figure S16. ^{1}H-NMR spectrum of **4**.

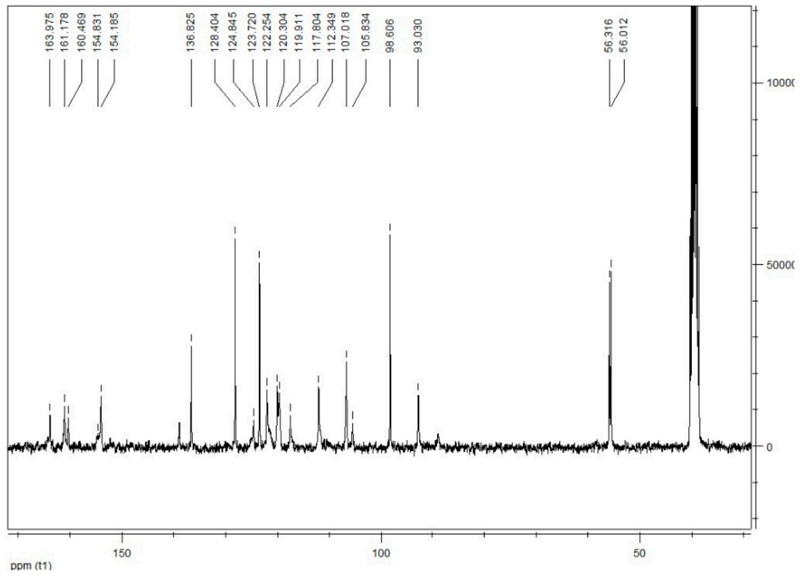

Figure S17. ^{13}C-NMR spectrum **4**.

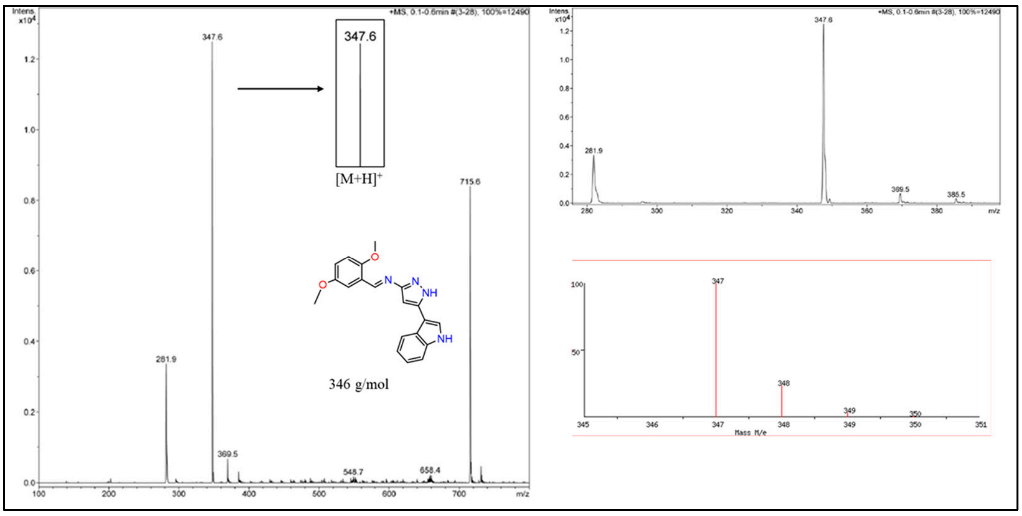

Figure S18. GM-MS (ESI) spectrum of **5**.

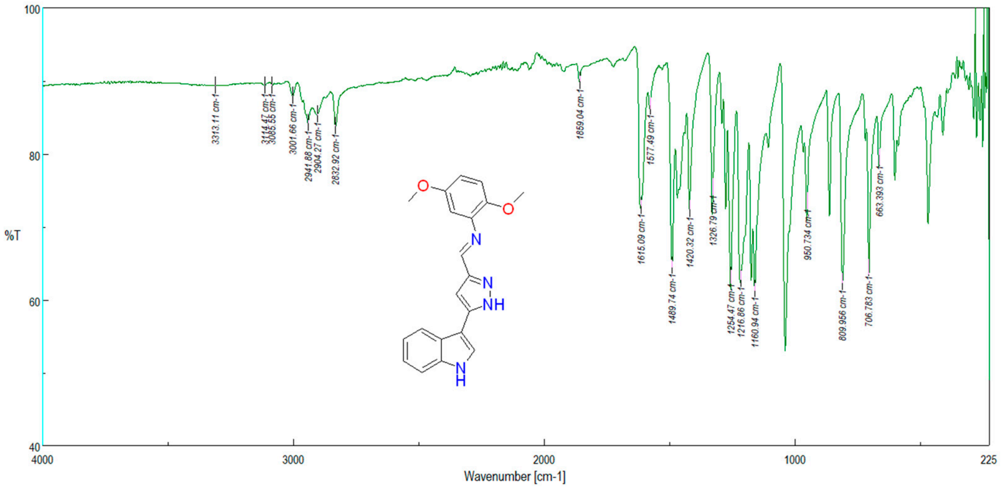

Figure S19. FT-IR (ATR) spectrum of **5**.

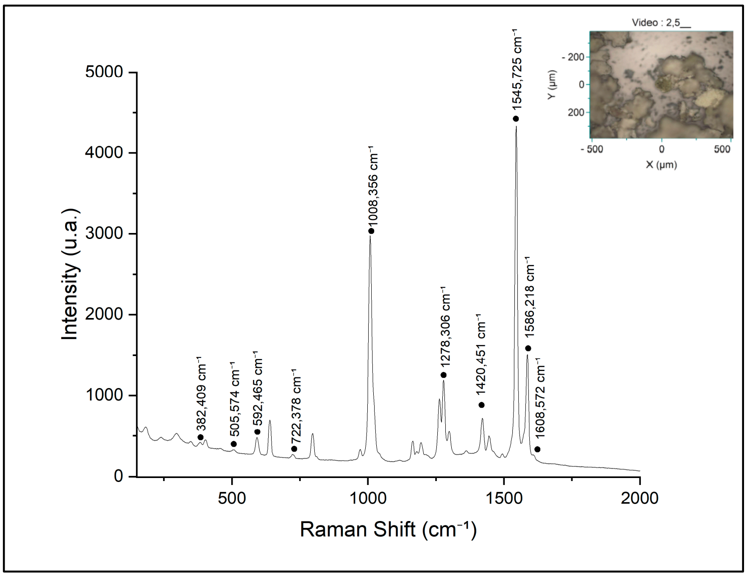

Figure S20. RAMAN (ATR) spectrum of **5**.

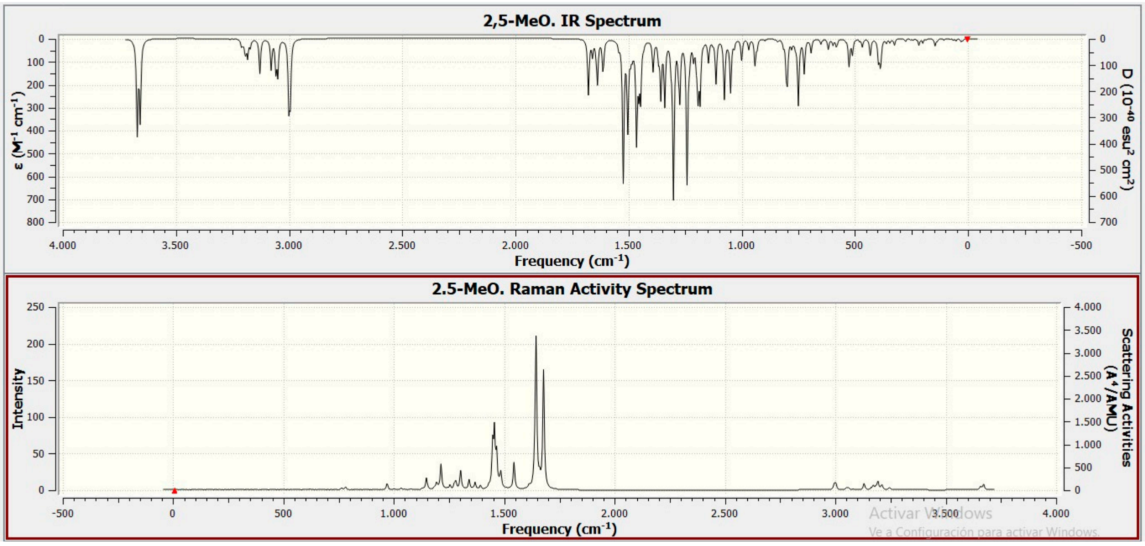

Figure S21. FT-IR (ATR) and Raman (ATR) theorical spectrums of **5**.

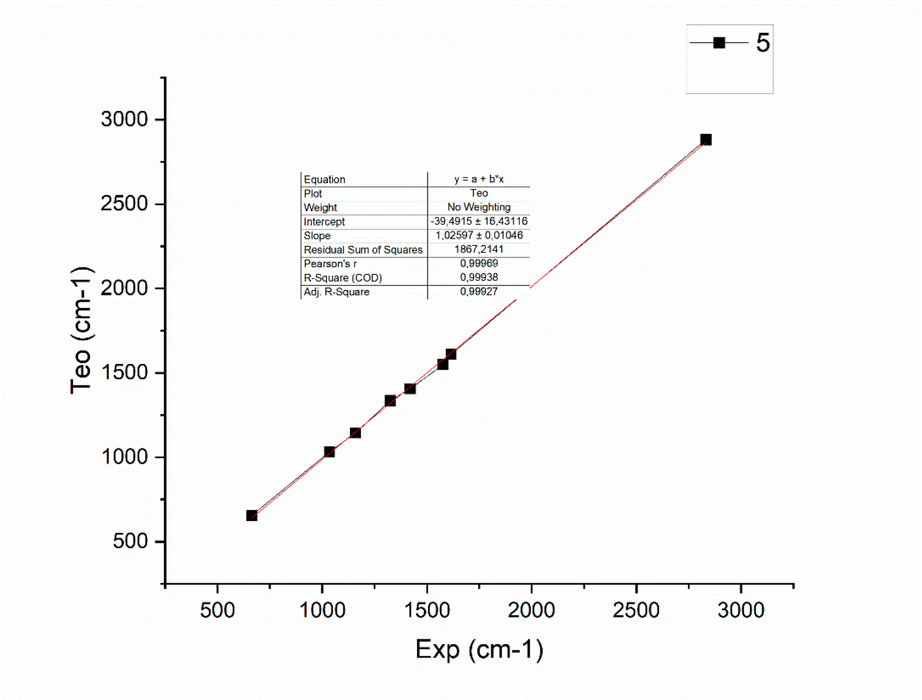

Figure S22. IR Correlation diagram of compound **5.**

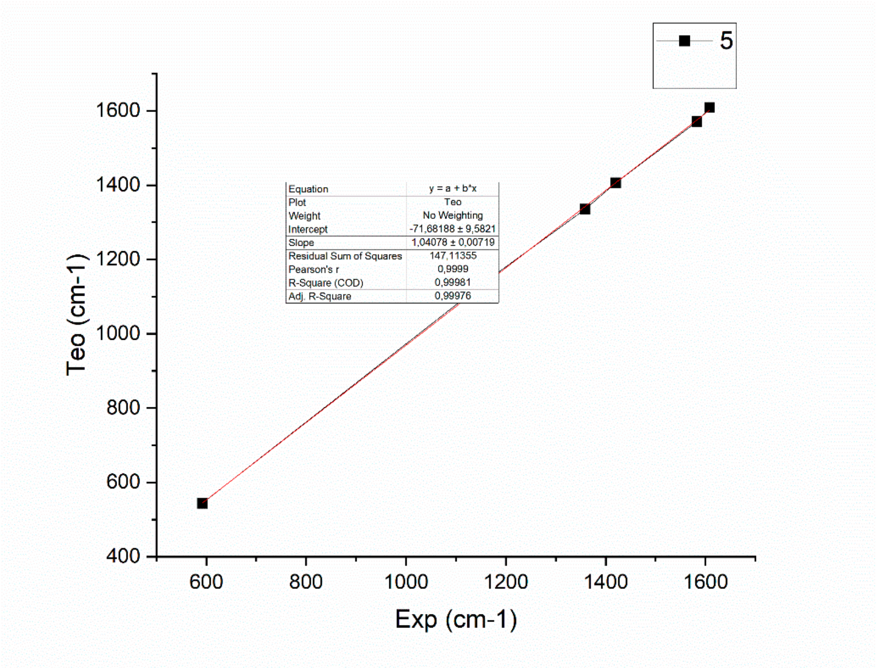

Figure S23. Raman Correlation diagram of compound **5.**

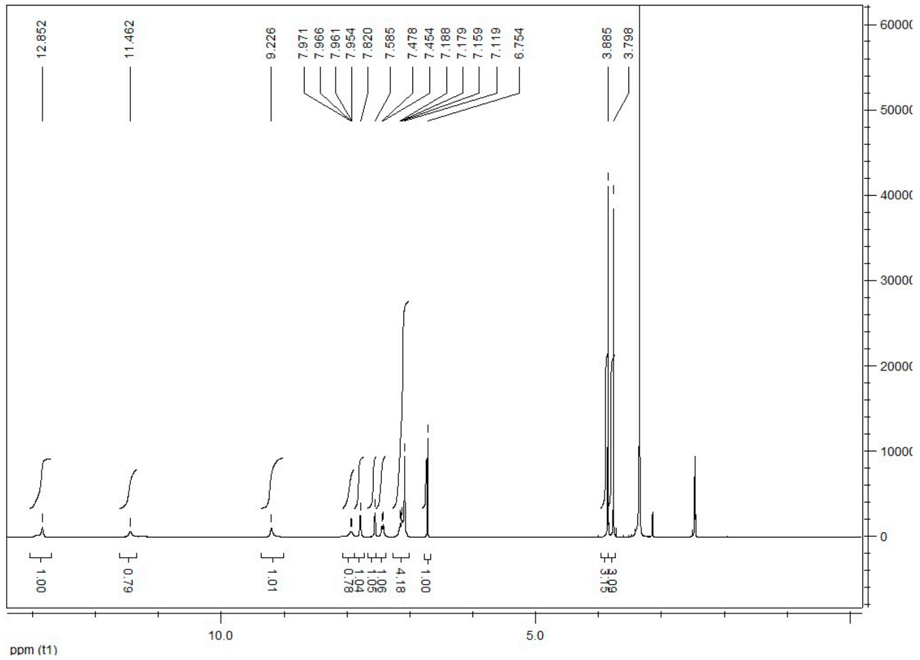

Figure S24. ^{1}H-NMR spectrum of **5**.

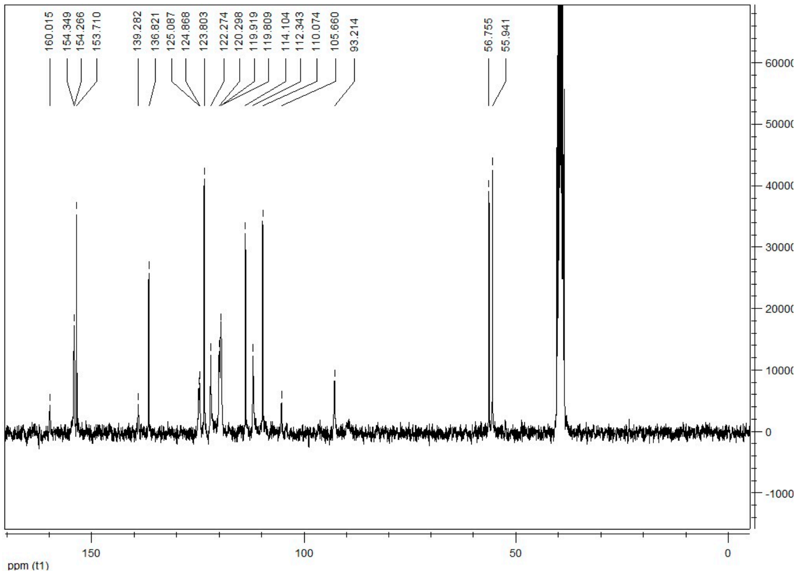

Figure S25. ^{13}C-NMR spectrum of **5**.

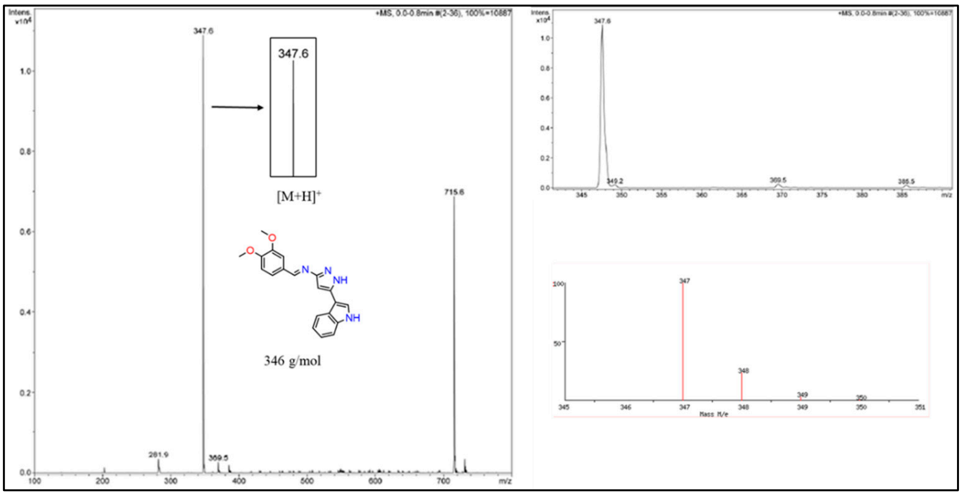

Figure S26. GM-MS (ESI) spectrum of **6**.

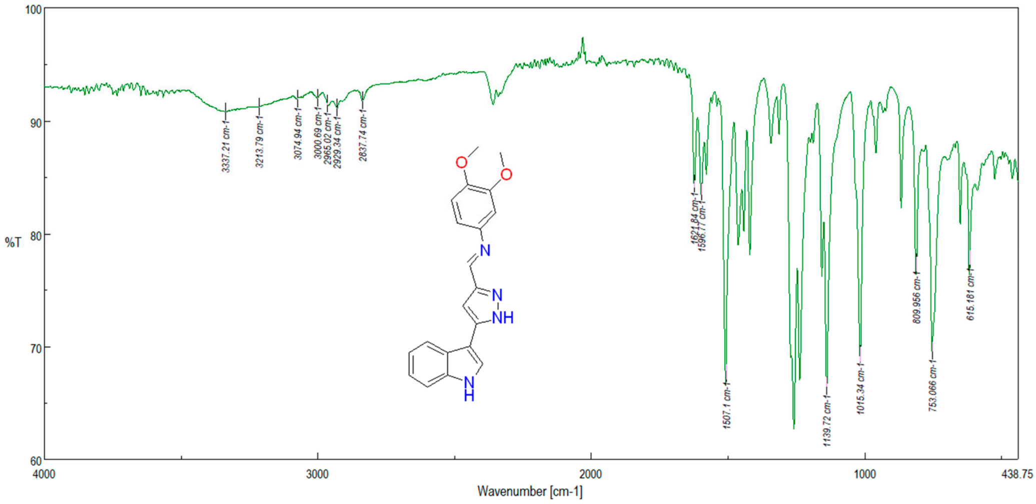

Figure S27. FT-IR (ATR) spectrum of **6**.

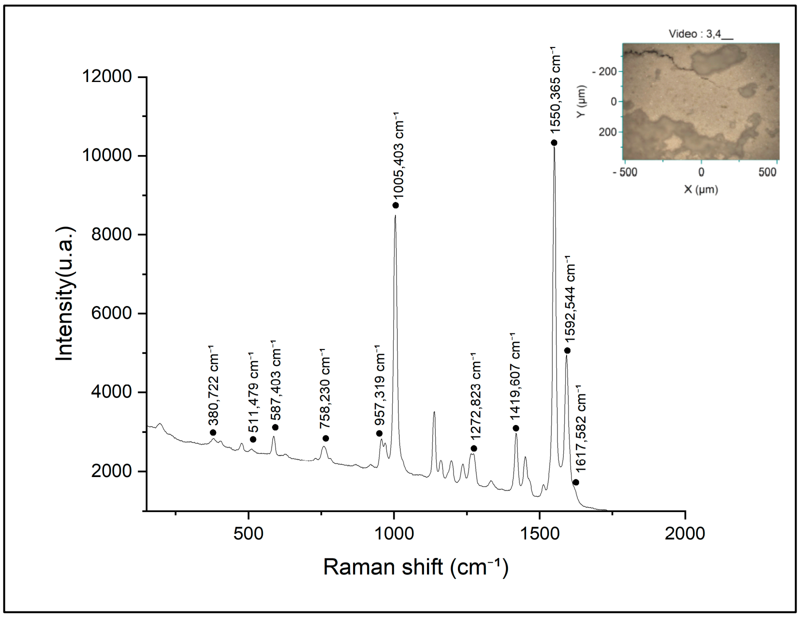

Figure S28: RAMAN (ATR) spectrum of **6**.

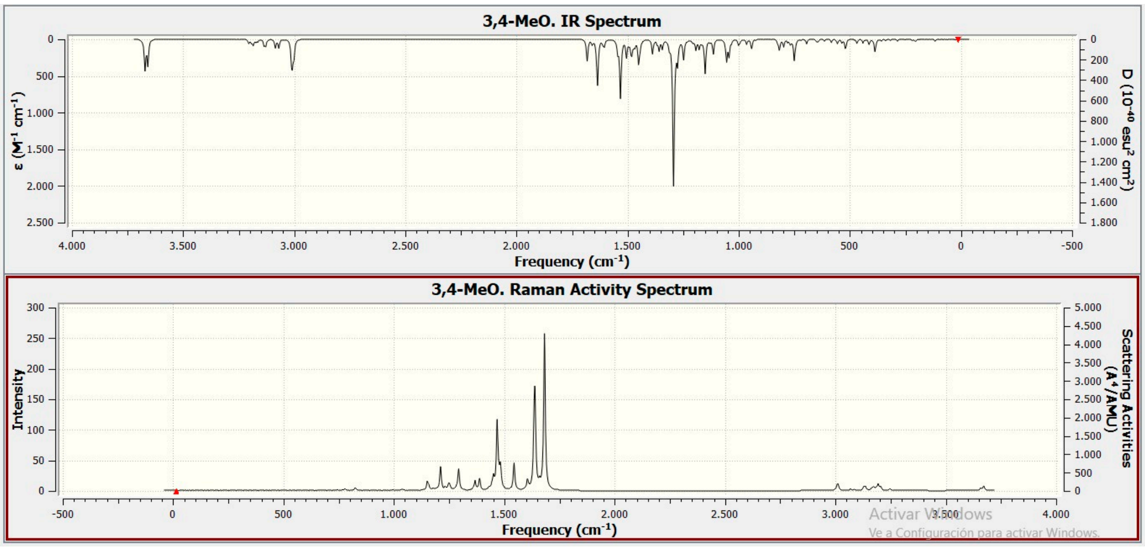

Figure S29. FT-IR (ATR) and Raman (ATR) theoretical spectrums of **6**.

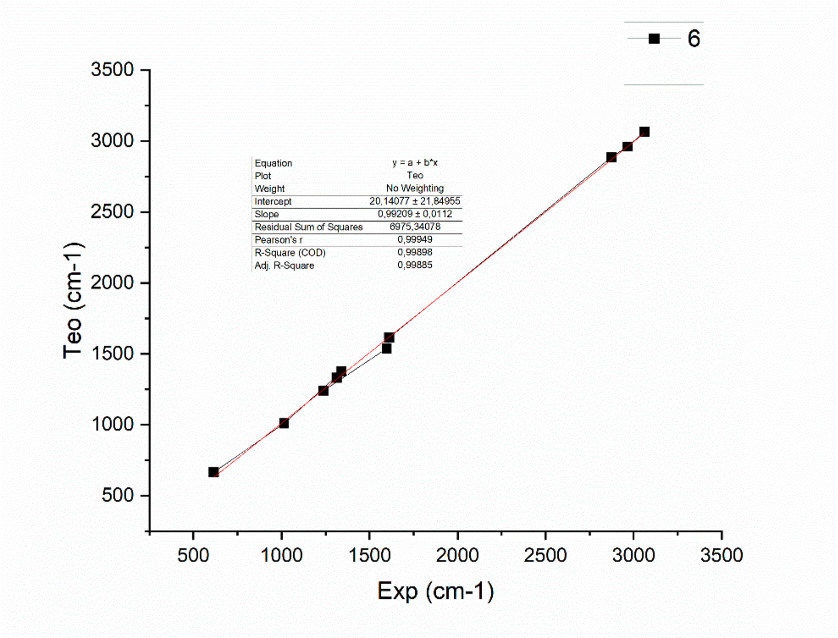

Figure S30. IR Correlation diagram of compound **6.**

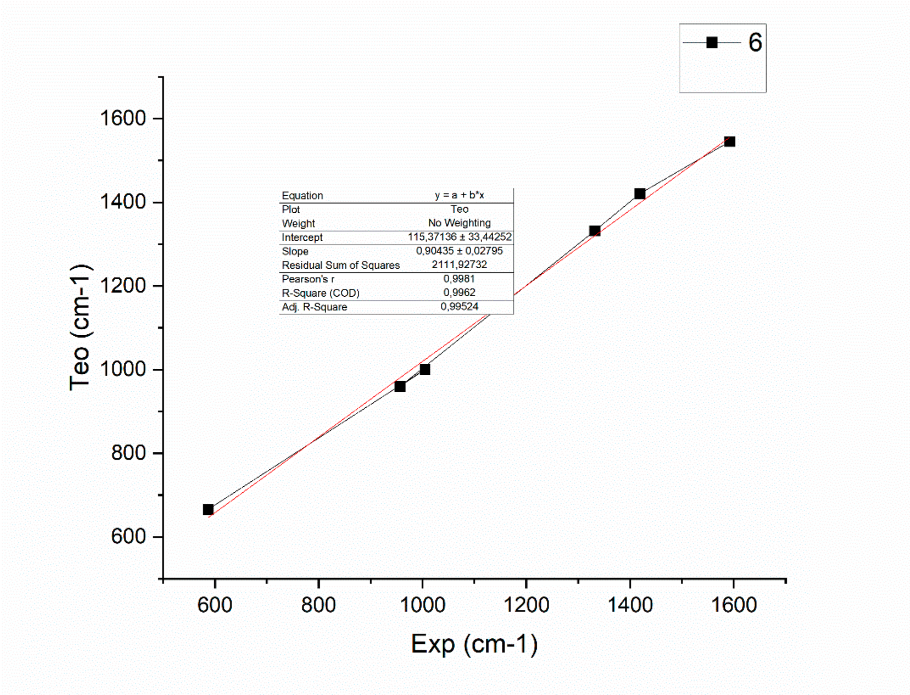

Figure S31. Raman Correlation diagram of compound **6.**

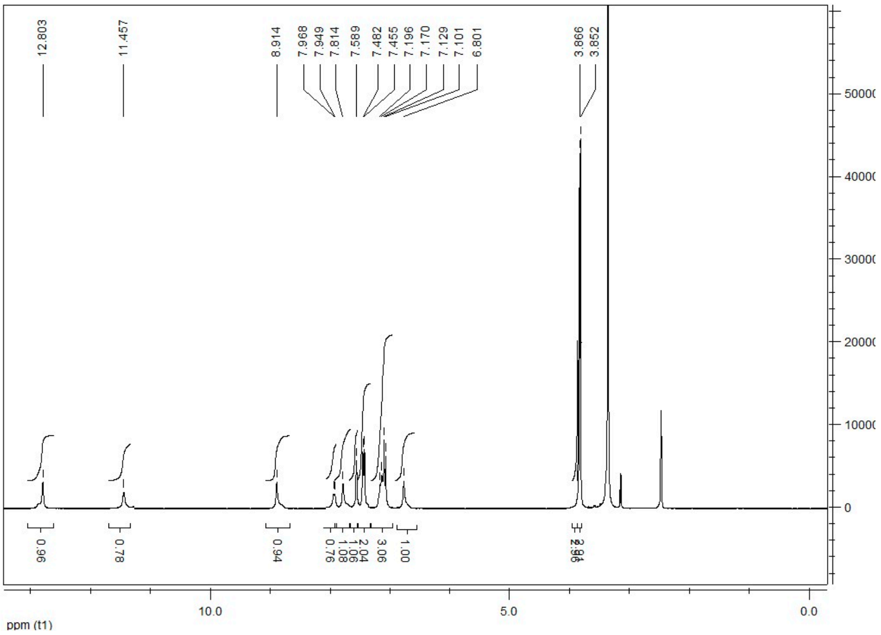

Figure S32. ^{1}H-NMR spectrum of **6**.

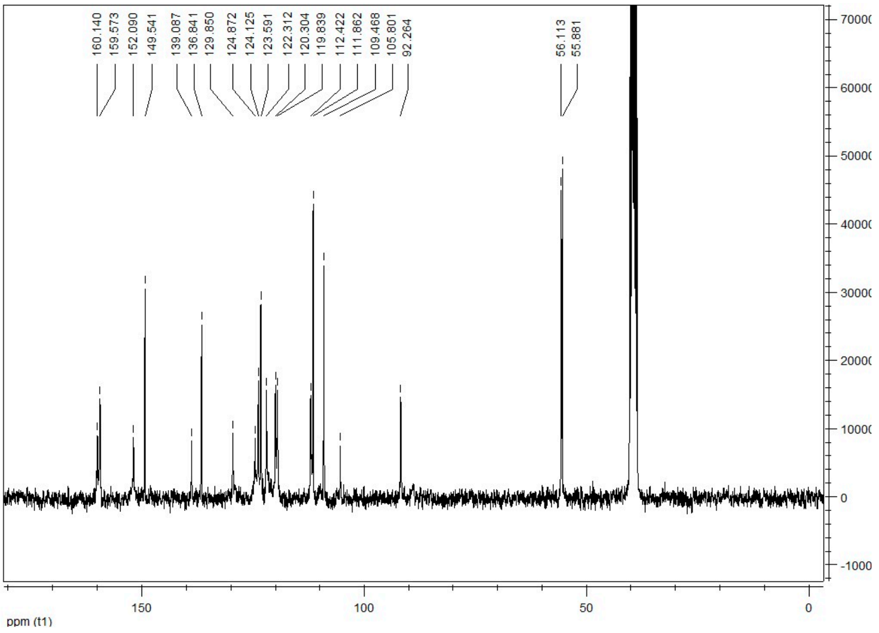

Figure S33. ^{13}C-NMR spectrum of **6**.

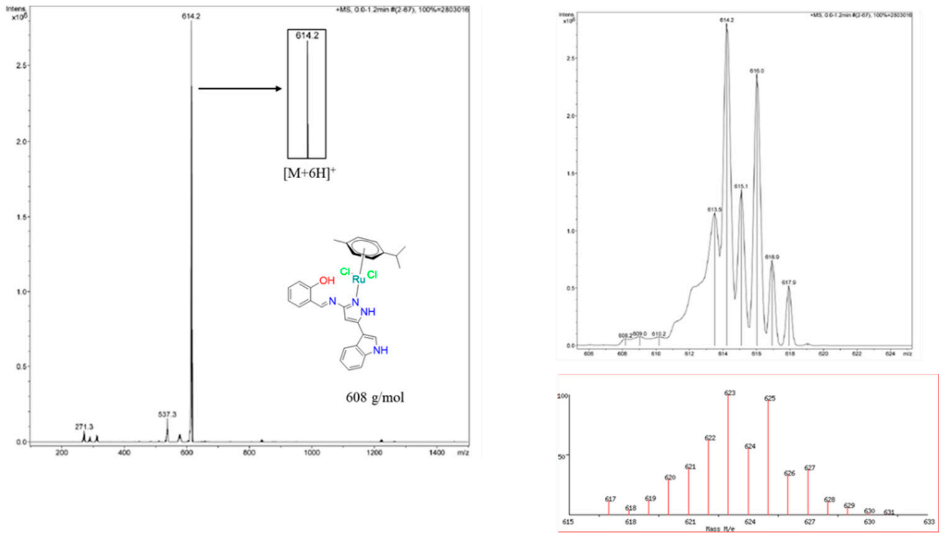

Figure S34. GM-MS (ESI) spectrum of **Ru3**.

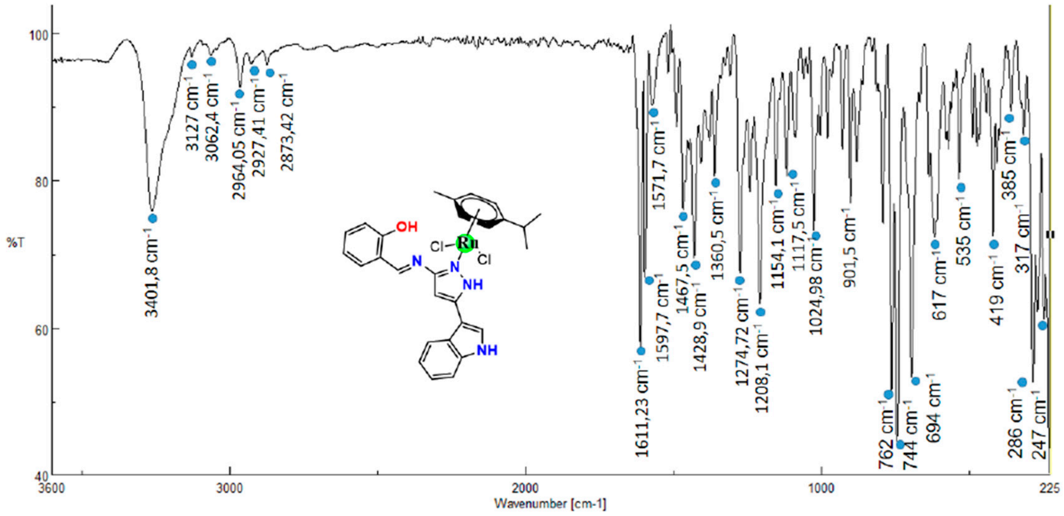

Figure S35. FT-IR (ATR) spectrum of **Ru3**.

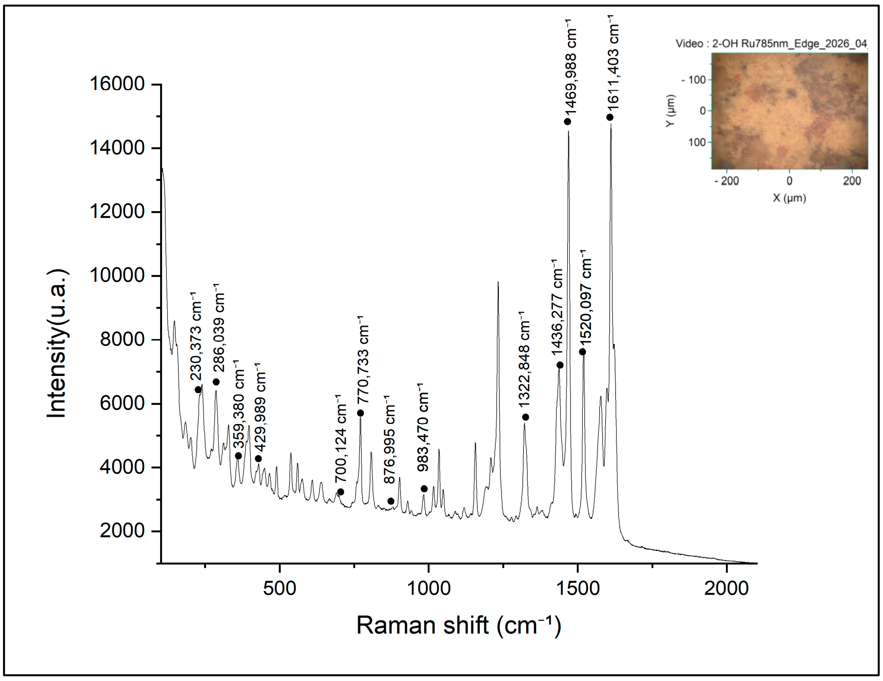

Figure S36. Raman (ATR) spectrum of **Ru3**.

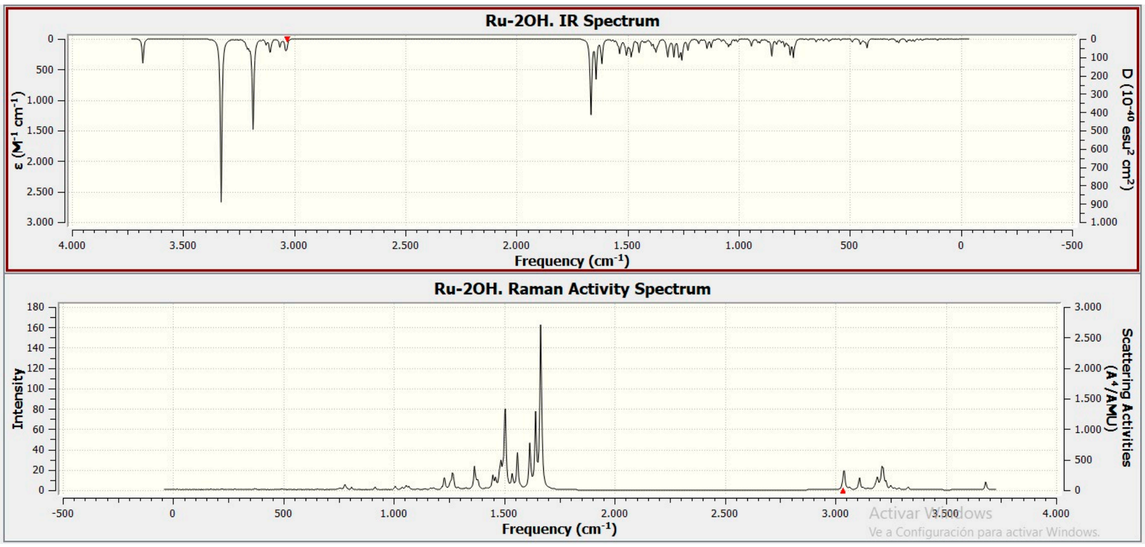

Figure S37. FT-IR (ATR) and Raman (ATR) theoretical spectrums of **Ru3**.

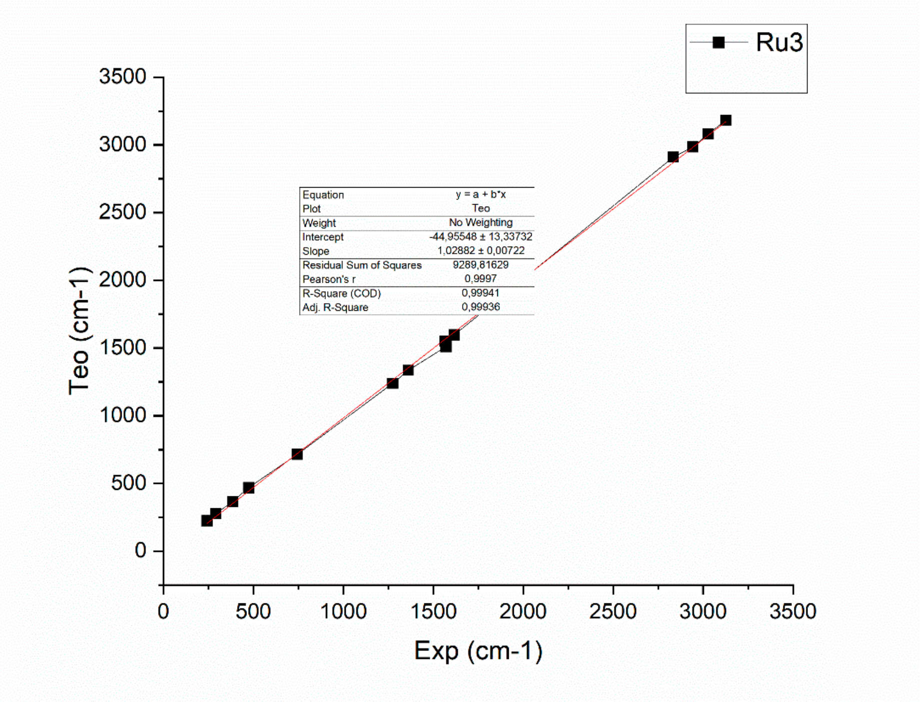

Figure S38. IR Correlation diagram of compound **Ru3.**

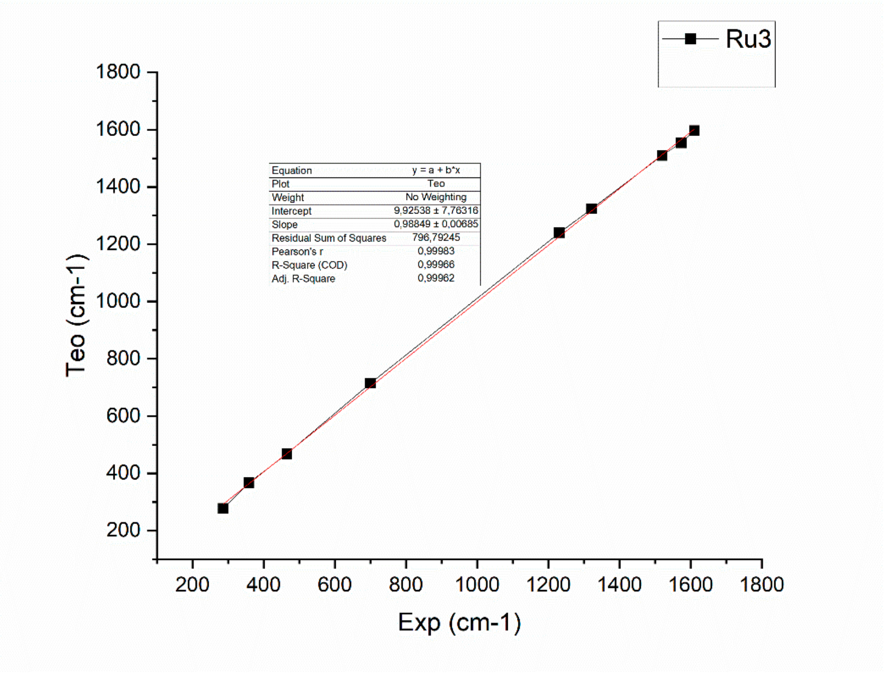

Figure S39. Raman Correlation diagram of compound **Ru3.**

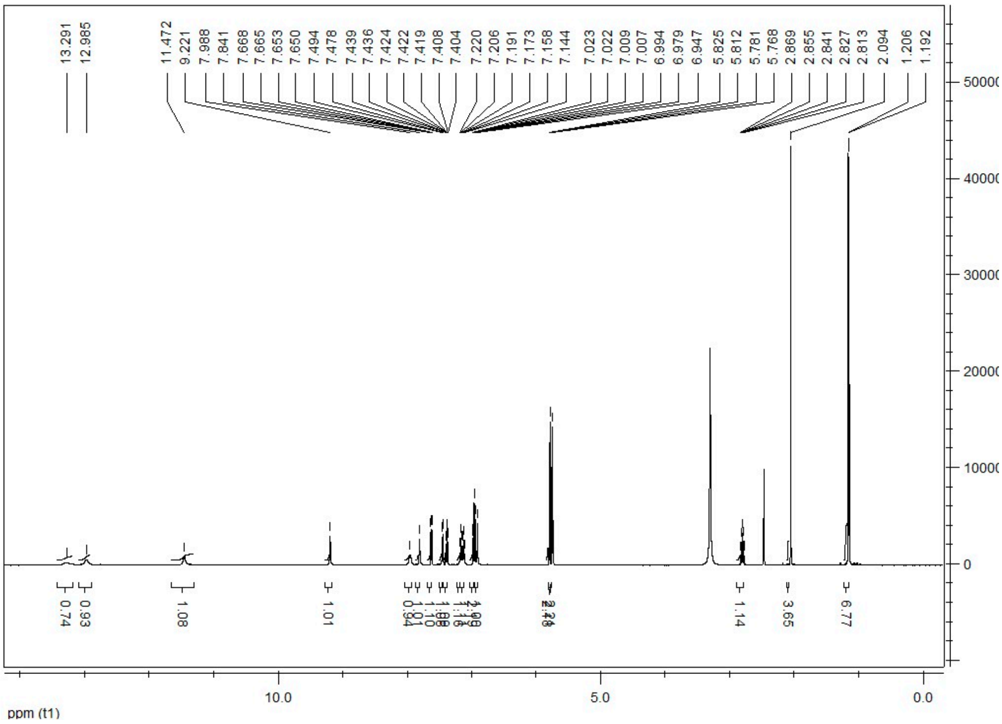

Figure S40. ^{1}H-NMR spectrum of **Ru3**.

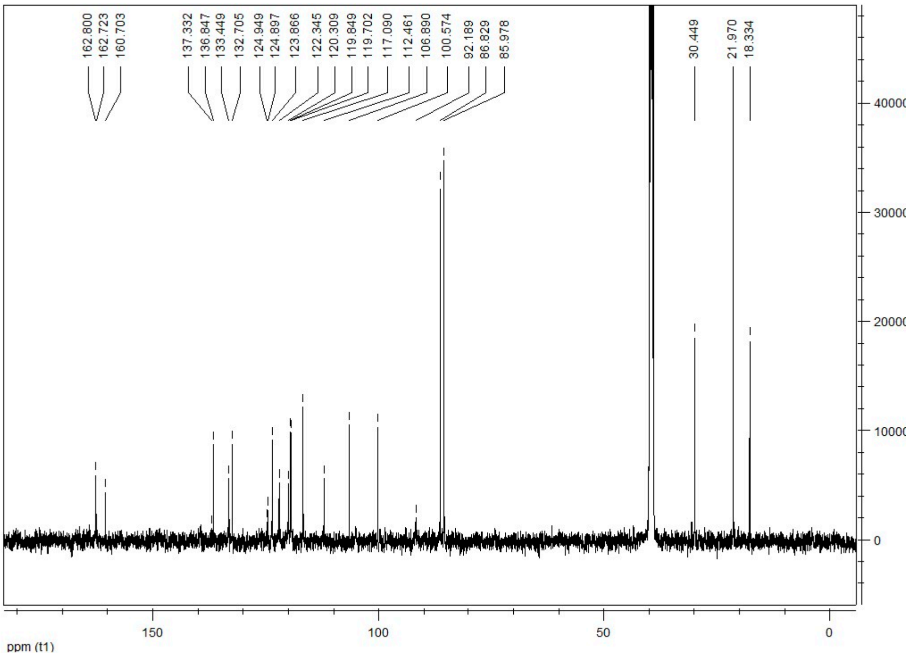

Figure S41. ^{13}C-NMR spectrum of **Ru3**.

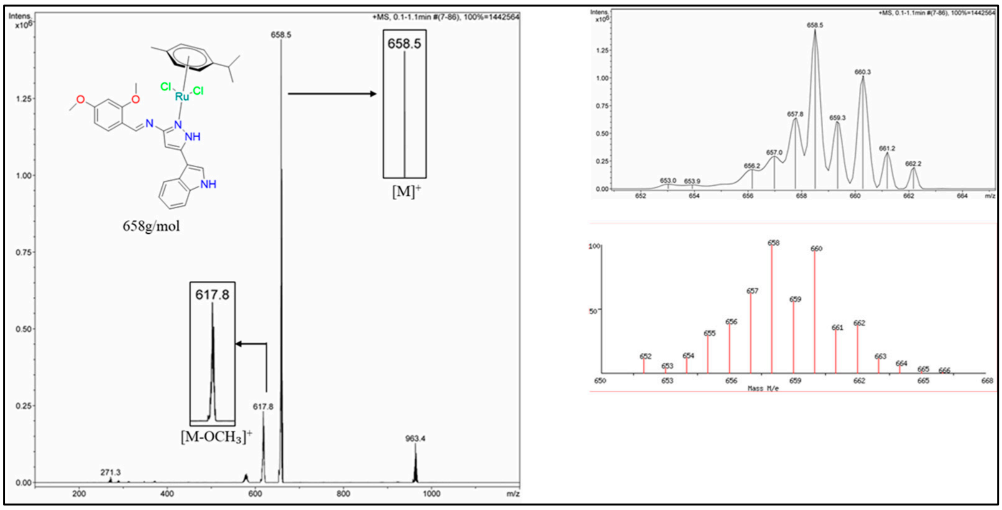

Figure S42. GM-MS (ESI) spectrum of **Ru4**.

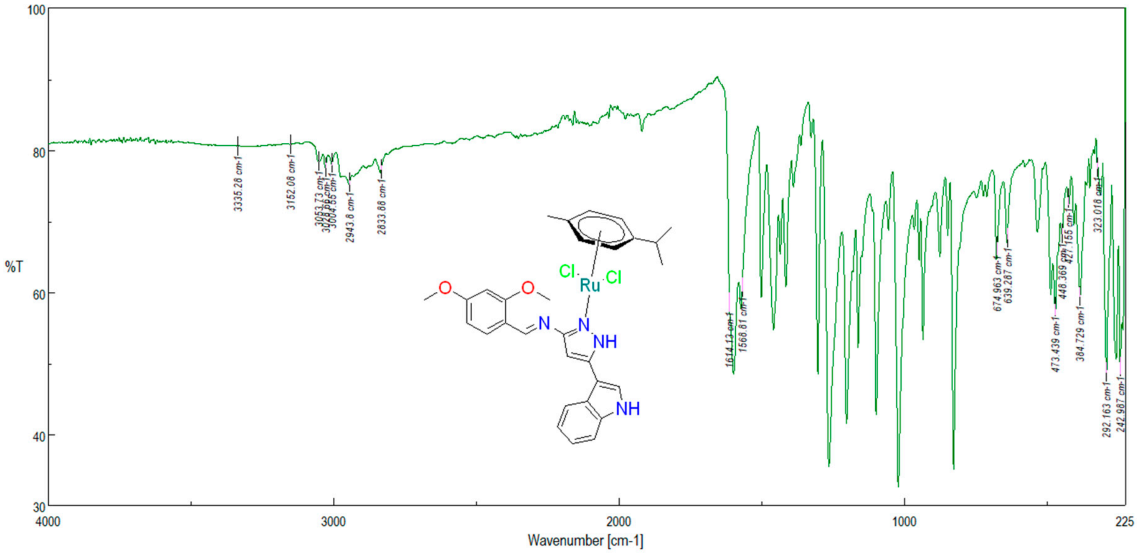

Figure S43. FT-IR(ATR) spectrum of **Ru4**.

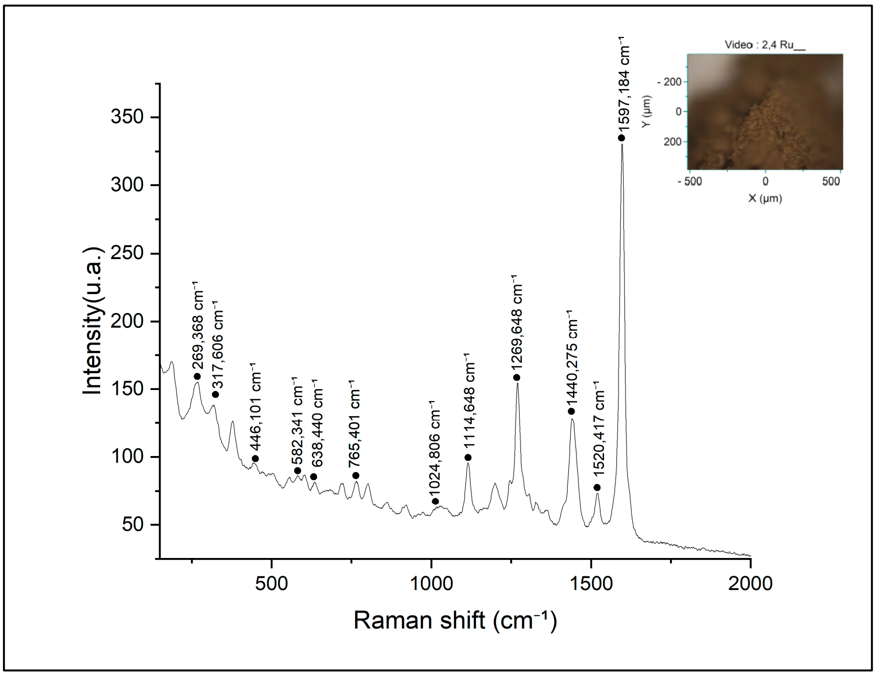

Figure S44. Raman (ATR) spectrum of **Ru4**.

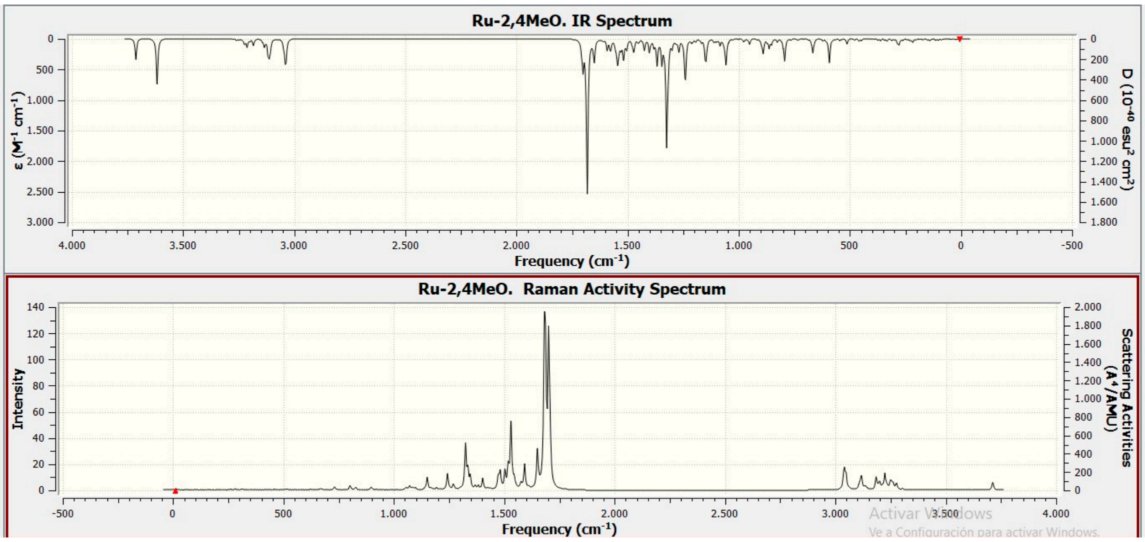

Figure S45. FT-IR (ATR) and Raman (ATR) theoretical spectrums of **Ru4**.

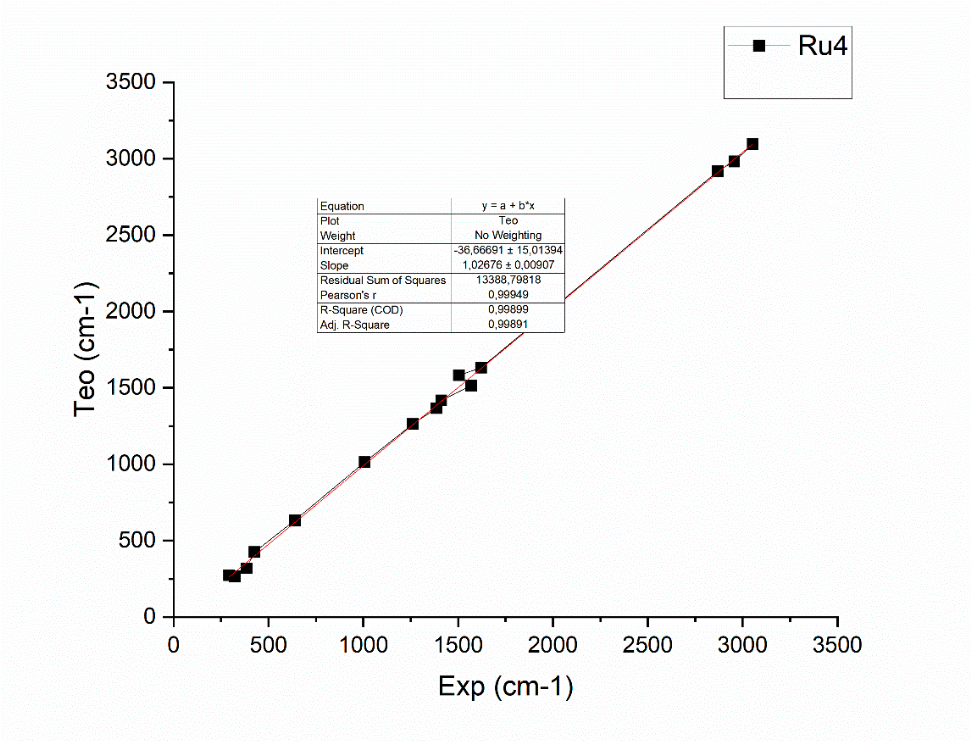

Figure S46. IR Correlation diagram of compound **Ru4.**

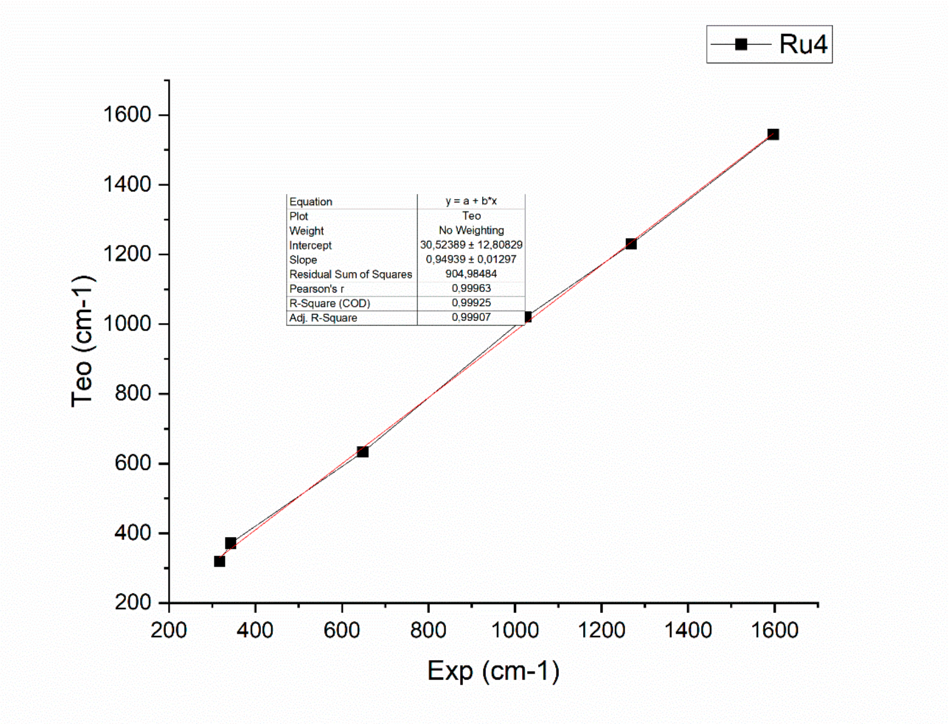

Figure S47. Raman Correlation diagram of compound **Ru4.**

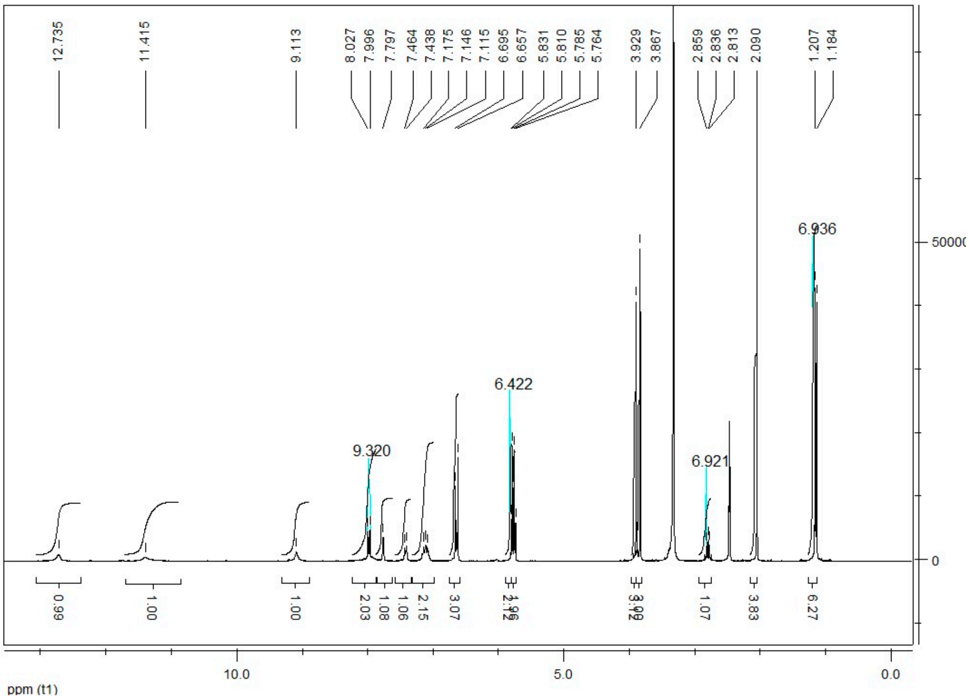

Figure S48. ^{1}H-NMR spectrum of **Ru4**.

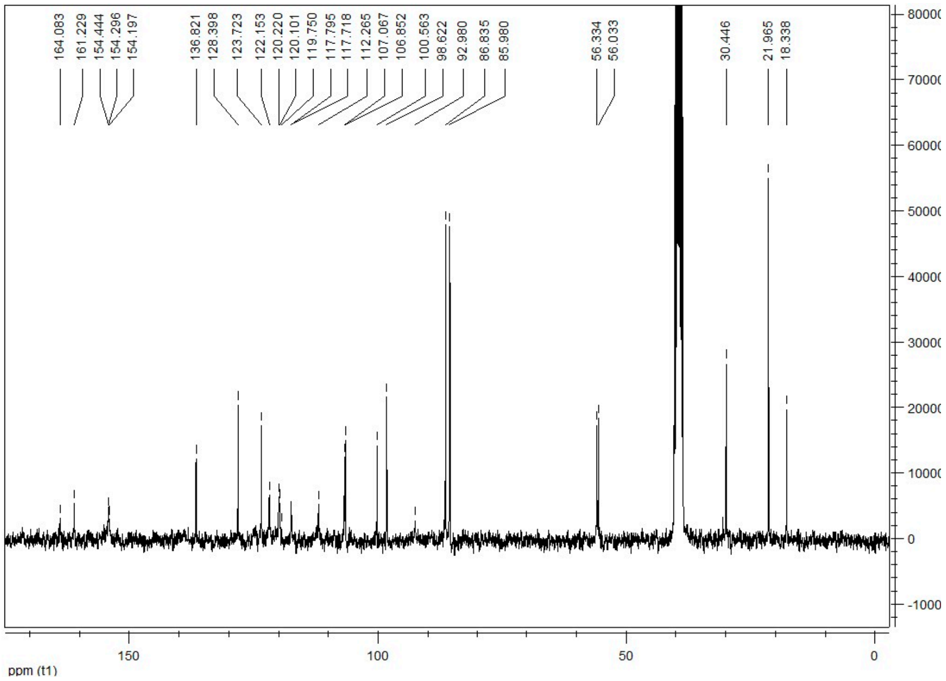

Figure S49. ^{13}C-NMR spectrum of **Ru4**.

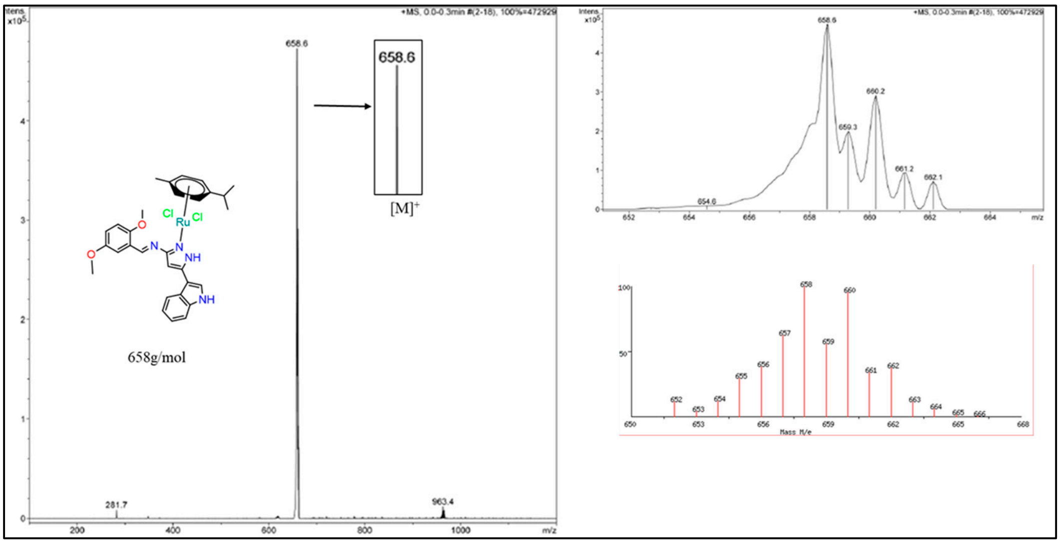

Figure S50. GM-MS (ESI) spectrum of **Ru5**.

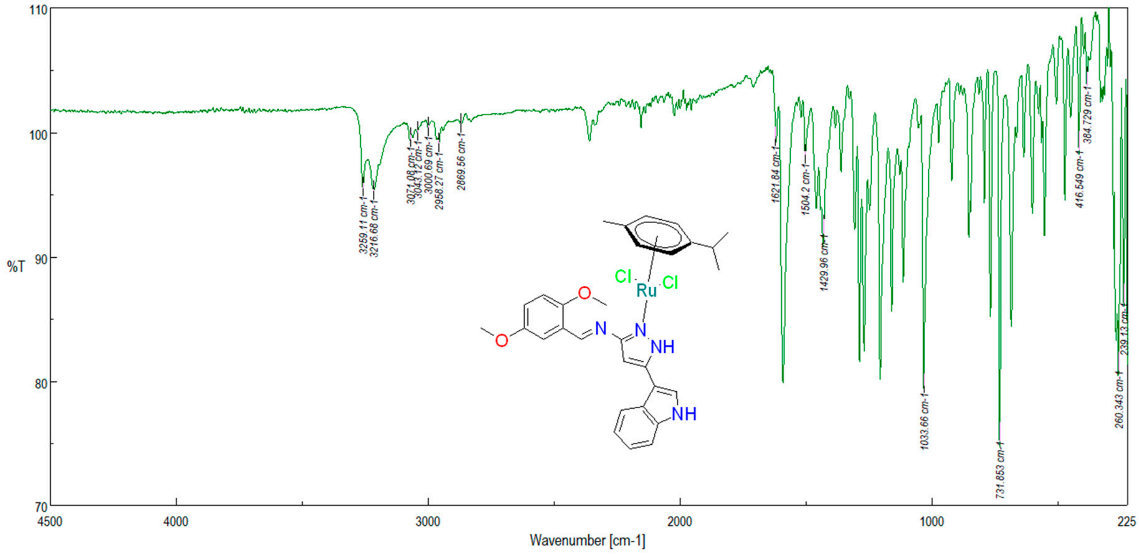

Figure S51. FT-IR (ATR) spectrum of **Ru5**.

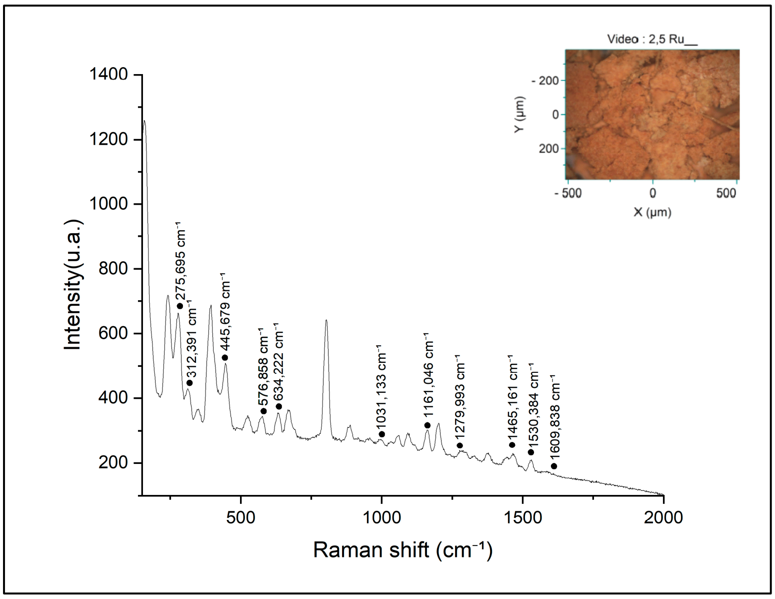

Figure S52. Raman (ATR) spectrum of **Ru5**.

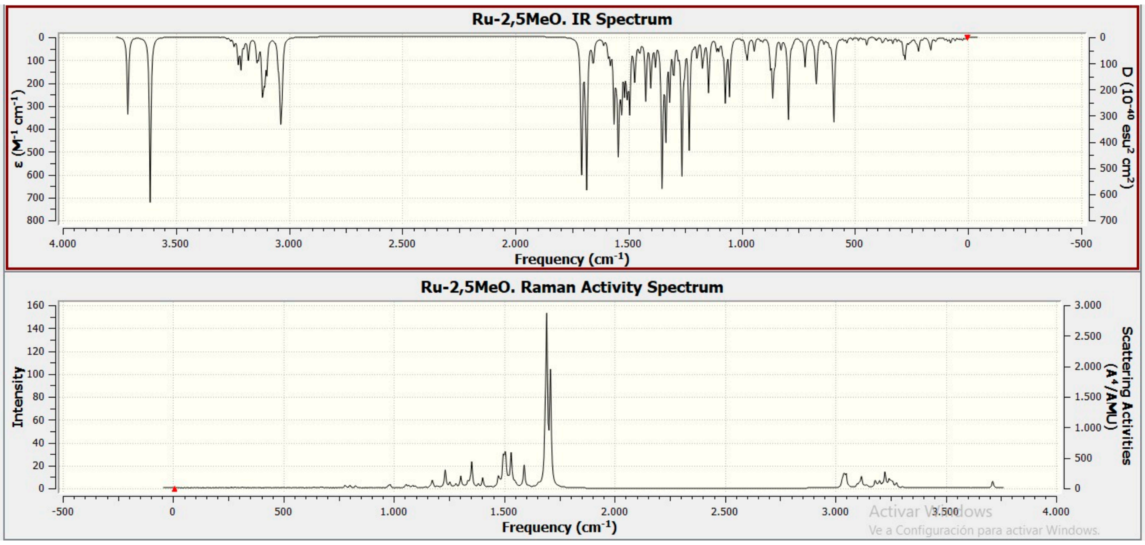

Figure S53. FT-IR (ATR) and Raman (ATR) theoretical spectrums of **Ru5**.

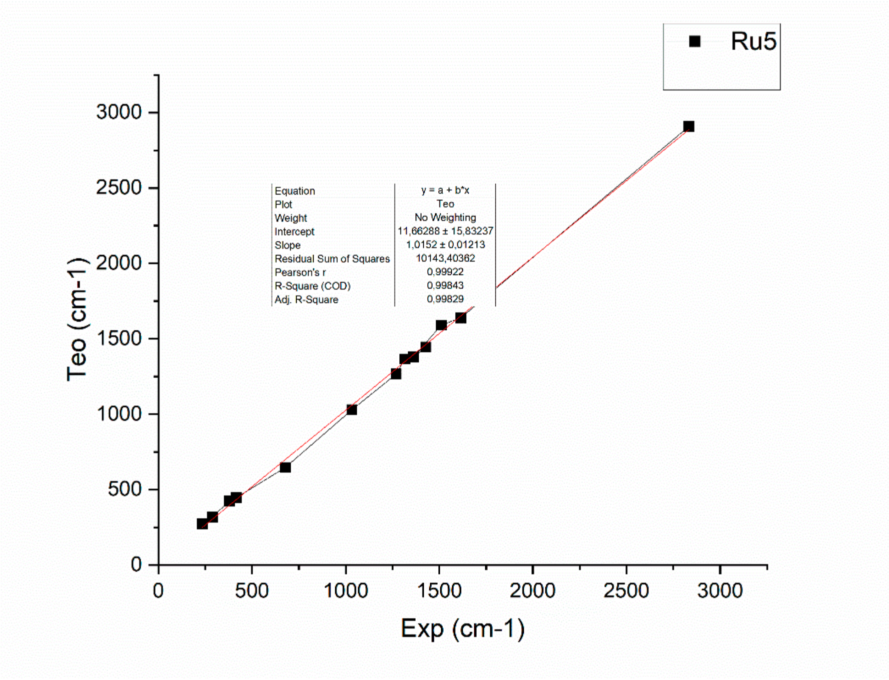

Figure S54. IR Correlation diagram of compound **Ru5.**

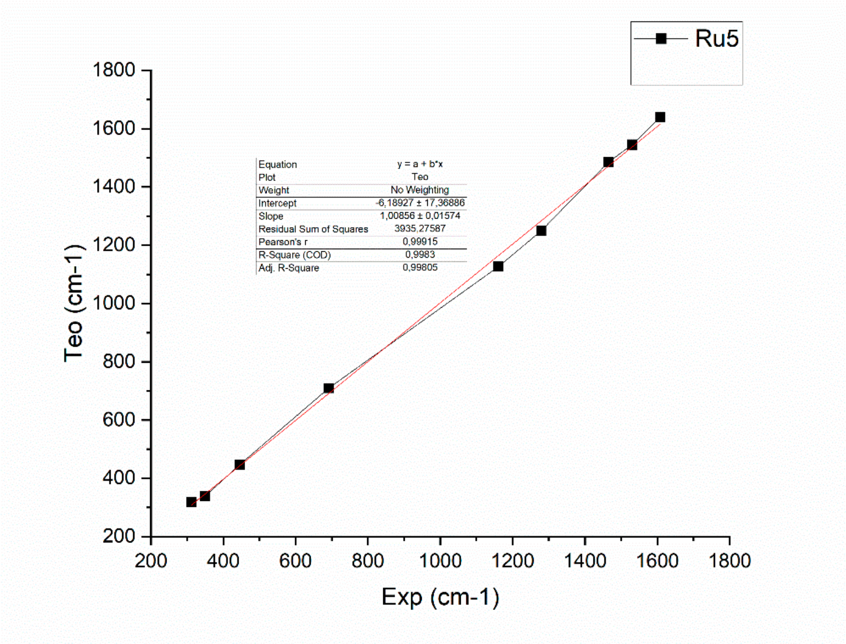

Figure S55. Raman Correlation diagram of compound **Ru5.**

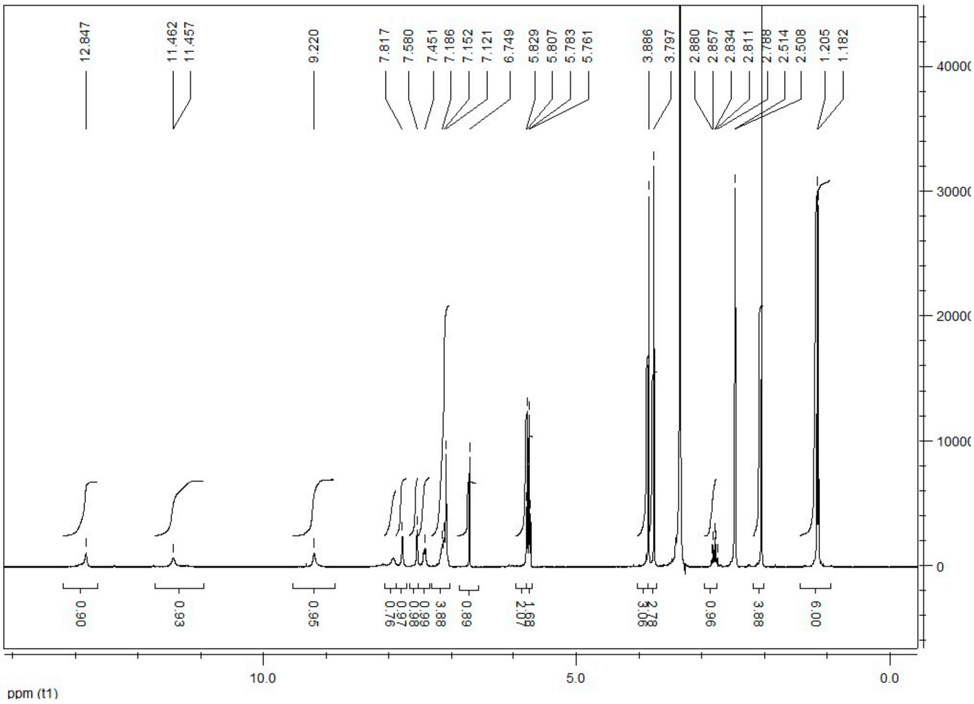

Figure S56. ^{1}H-NMR spectrum of **Ru5**.

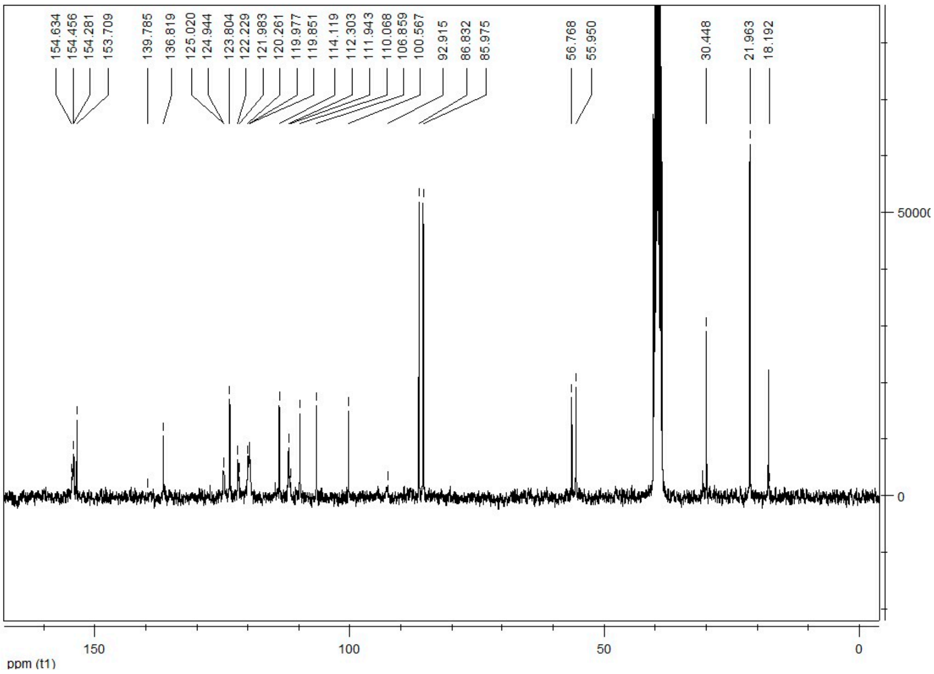

Figure S57. ^{13}C-NMR spectrum of **Ru5**.

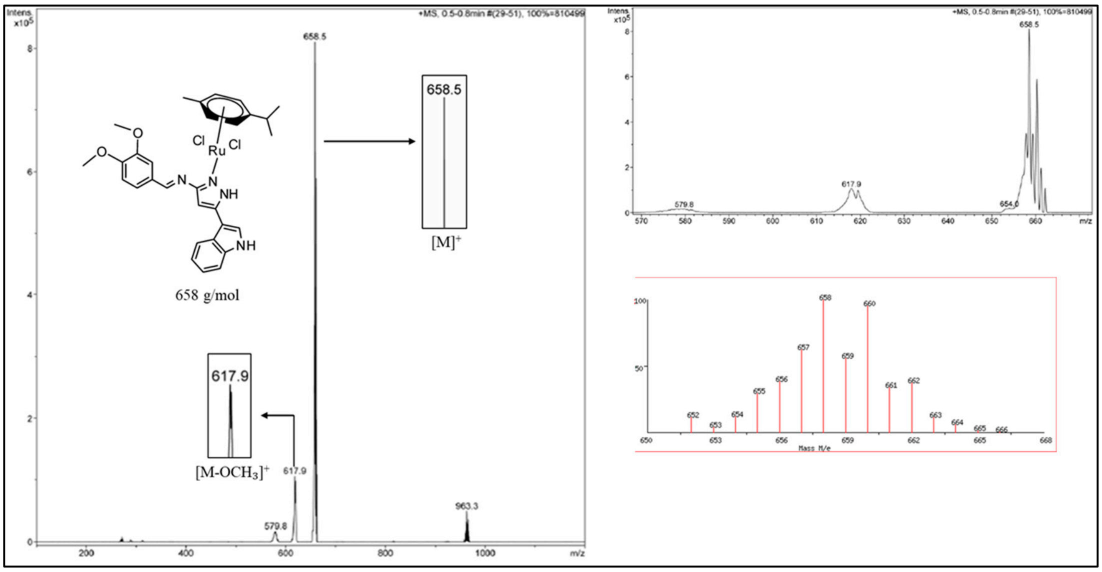

Figure S58. GM-MS (ESI) spectrum of **Ru6**.

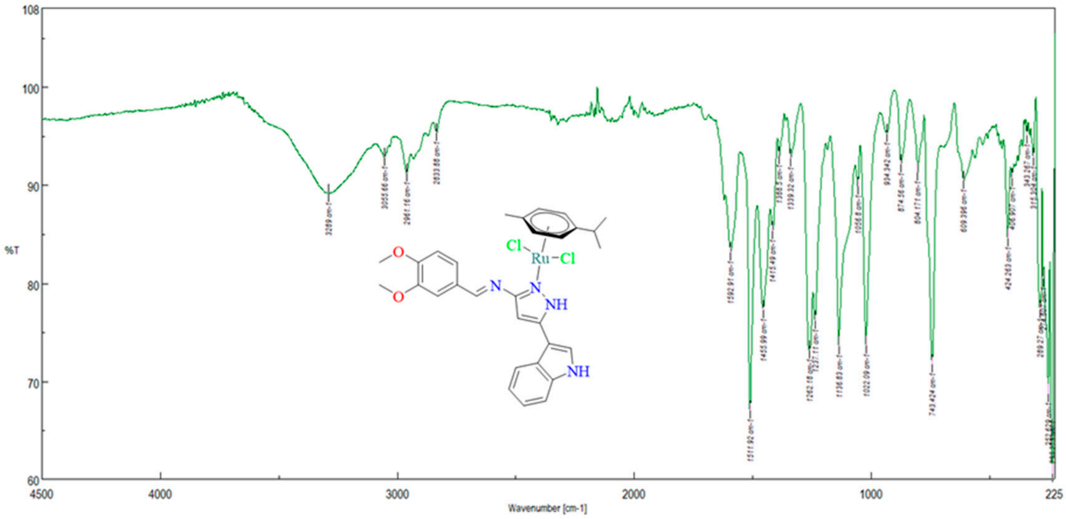

Figure S59: FT-IR (ATR) spectrum of **Ru6**.

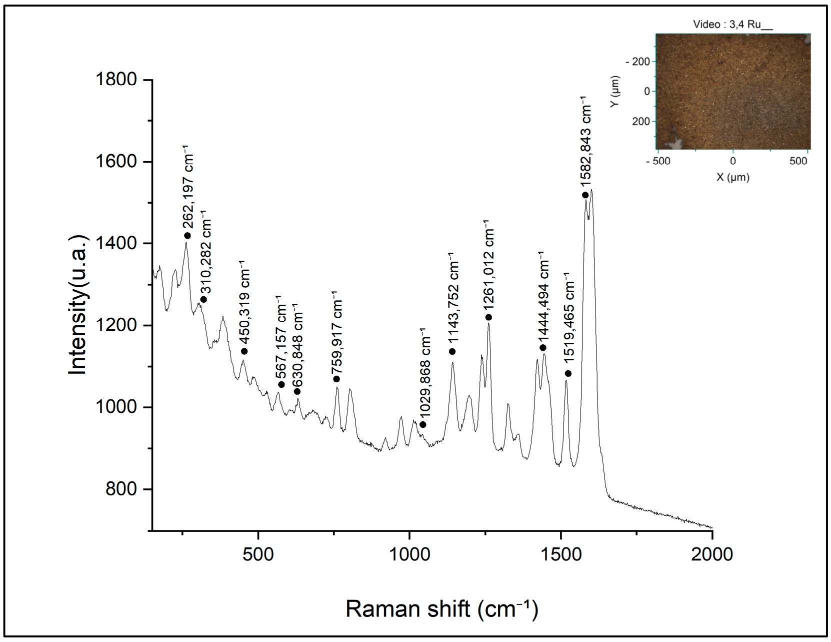

Figure S60. Raman (ATR) spectrum of **Ru6**.

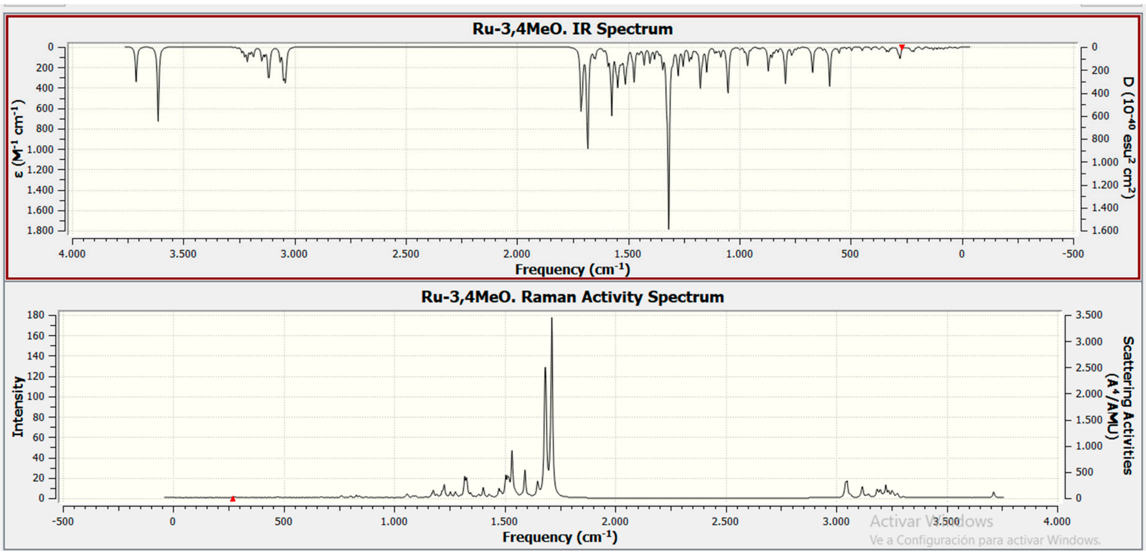

Figure S61. FT-IR (ATR) and Raman (ATR) theoretical spectrums of **Ru6**.

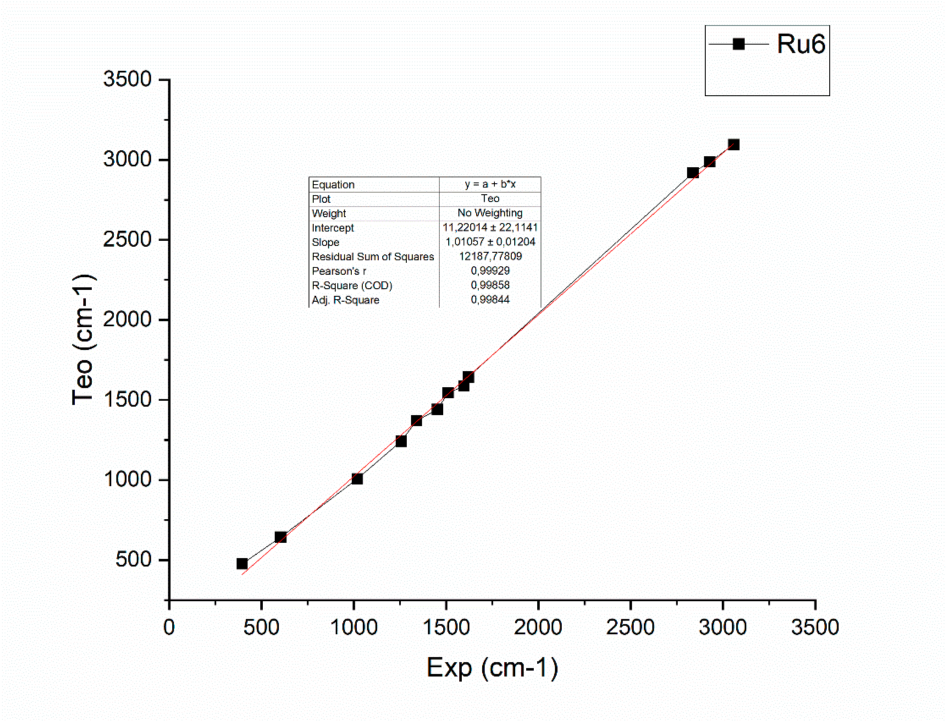

Figure S62. IR Correlation diagram of compound **Ru6.**

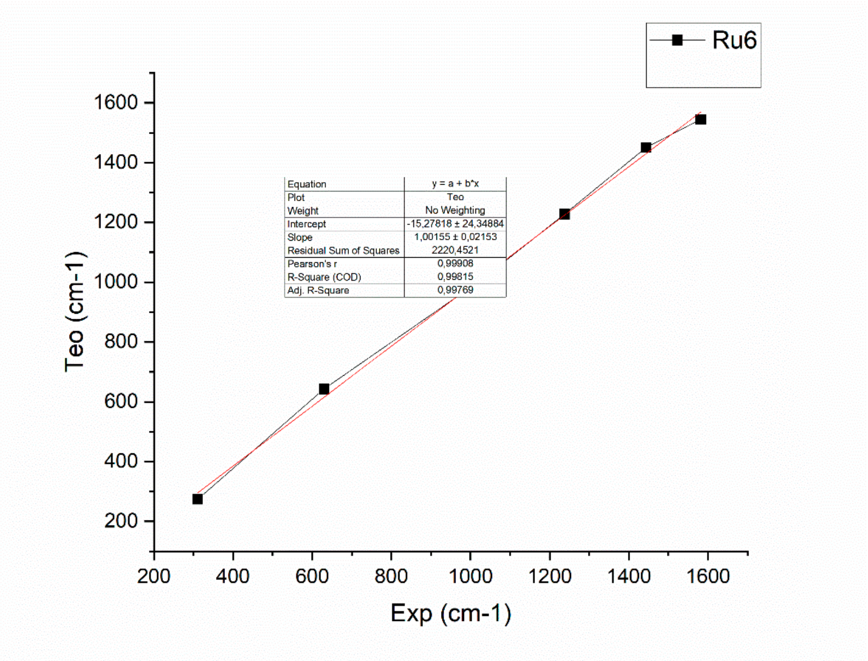

Figure S63. Raman Correlation diagram of compound **Ru6.**

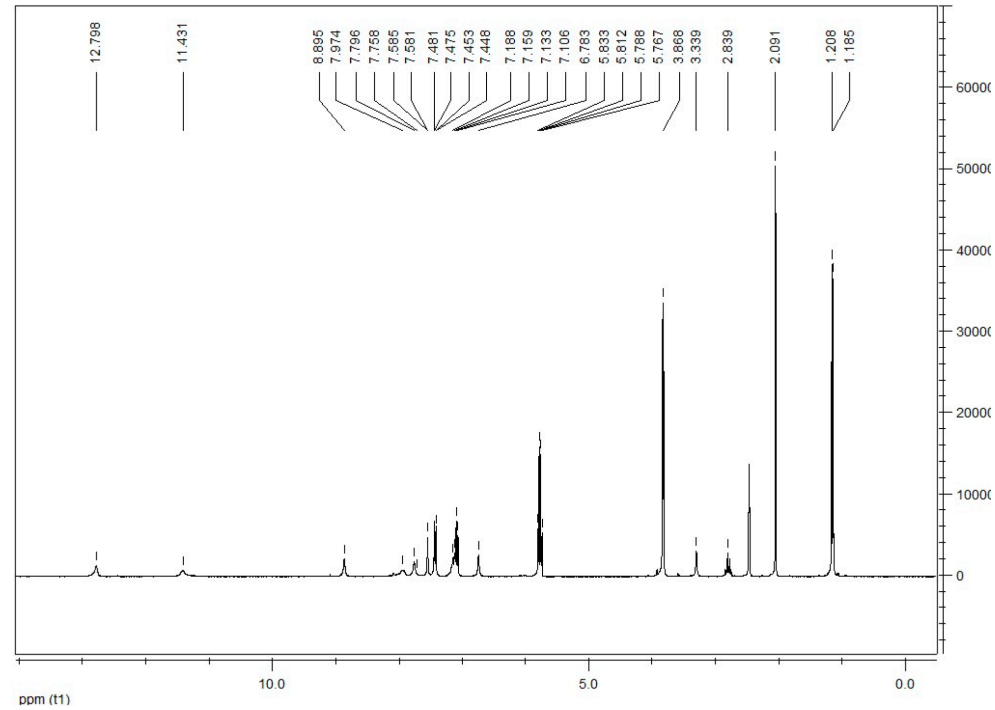

Figure S64. ^{1}H-NMR spectrum of **Ru6**.

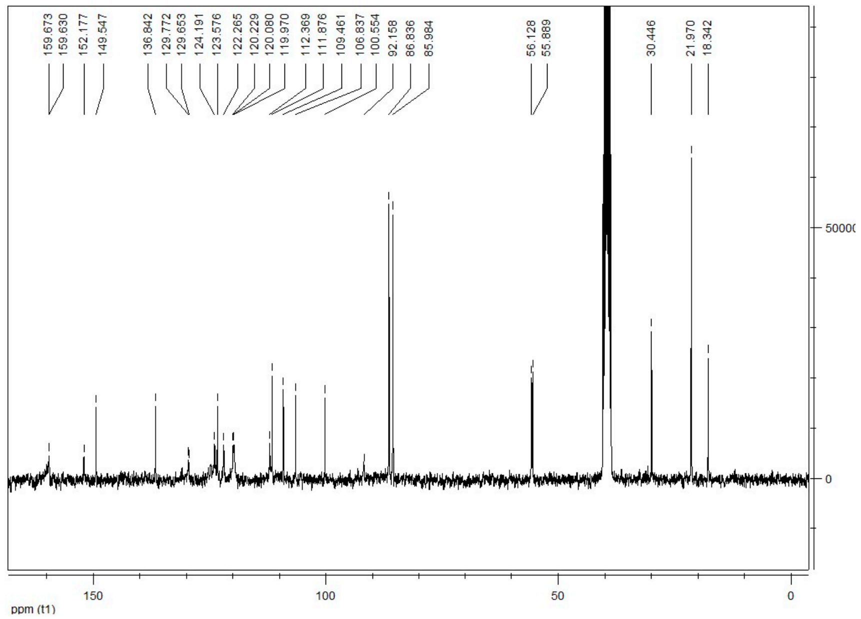

Figure S65. ^{13}C-NMR spectrum of **Ru6**.

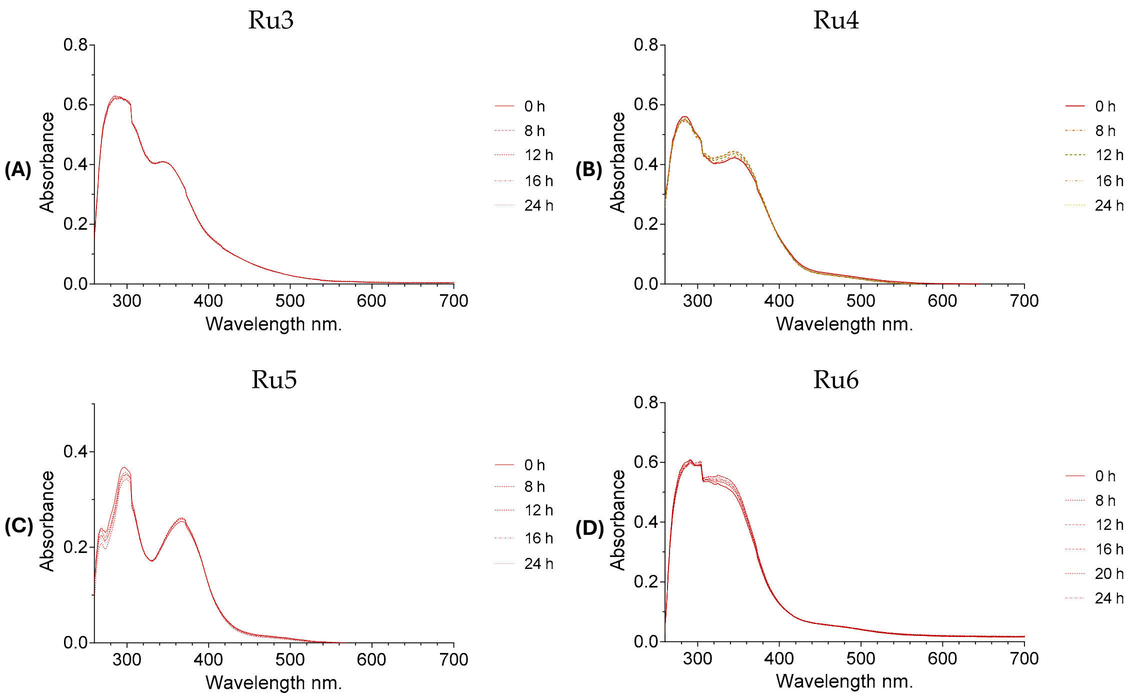

Figure S66. UV–vis spectra of **Ru3** (**A**), **Ru4** (**B**), **Ru5** (**C**) and **Ru6** (**D**) at 10 μM in a PBS/DMSO (0.1%) solution for 24 h at 25 °C.

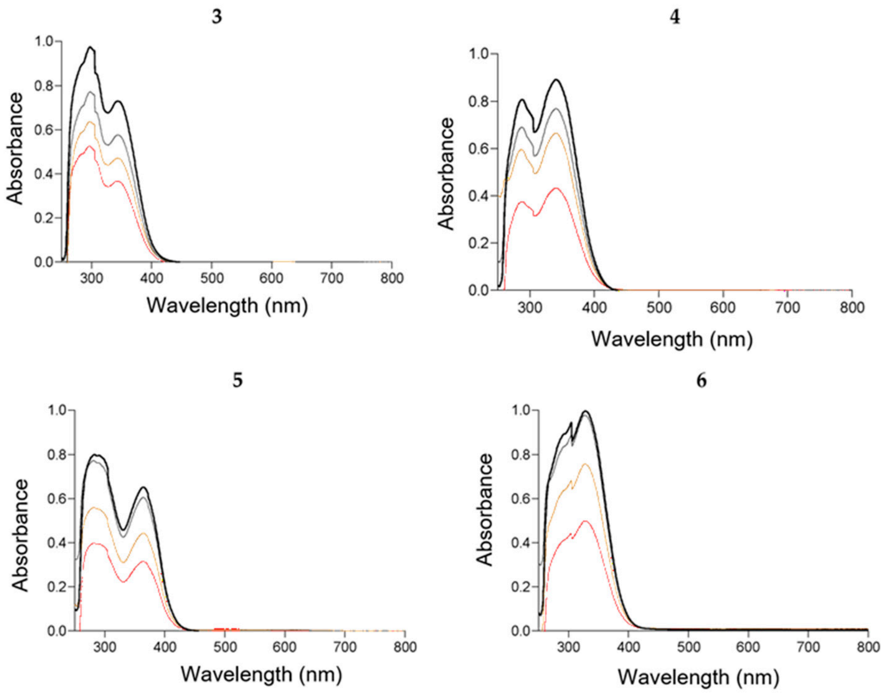

Figure S67. Experimental UV–vis spectra of **3-6.**

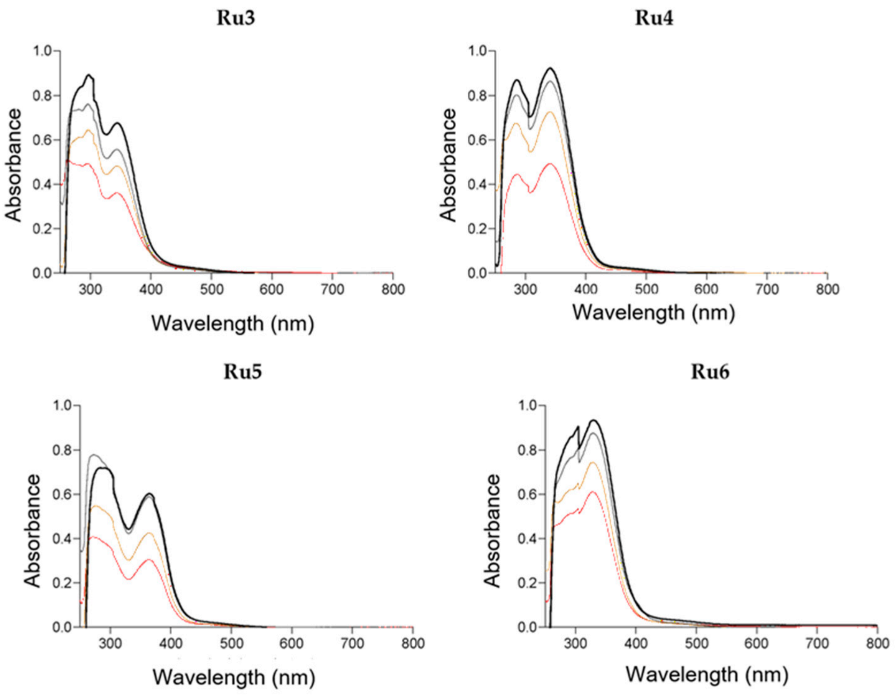

Figure S68. Experimental UV–vis spectra of **Ru3-Ru6.**

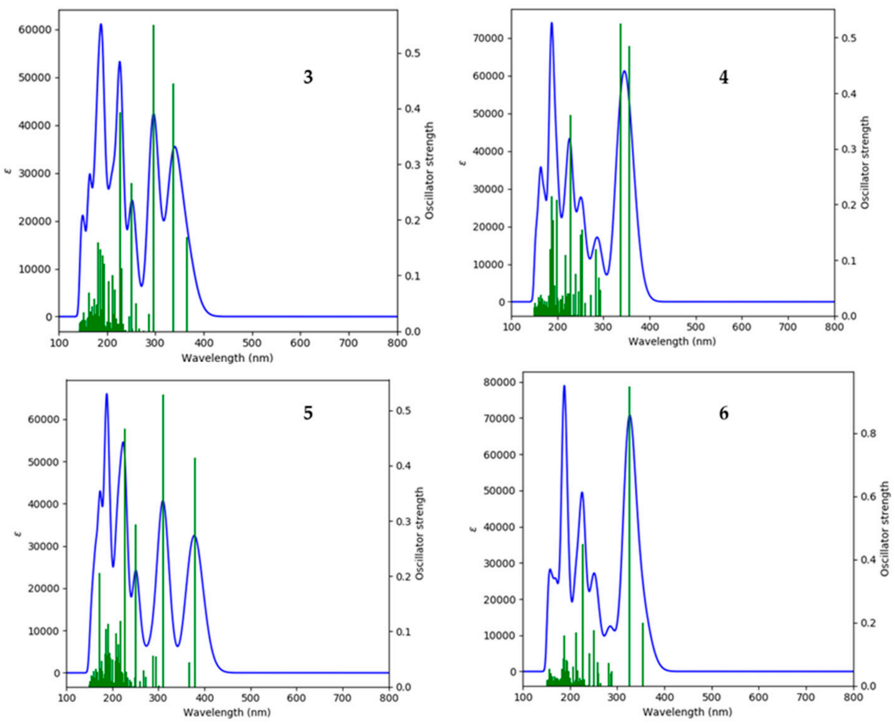

Figure S69. Theoretical UV–vis spectra of **3-6.**

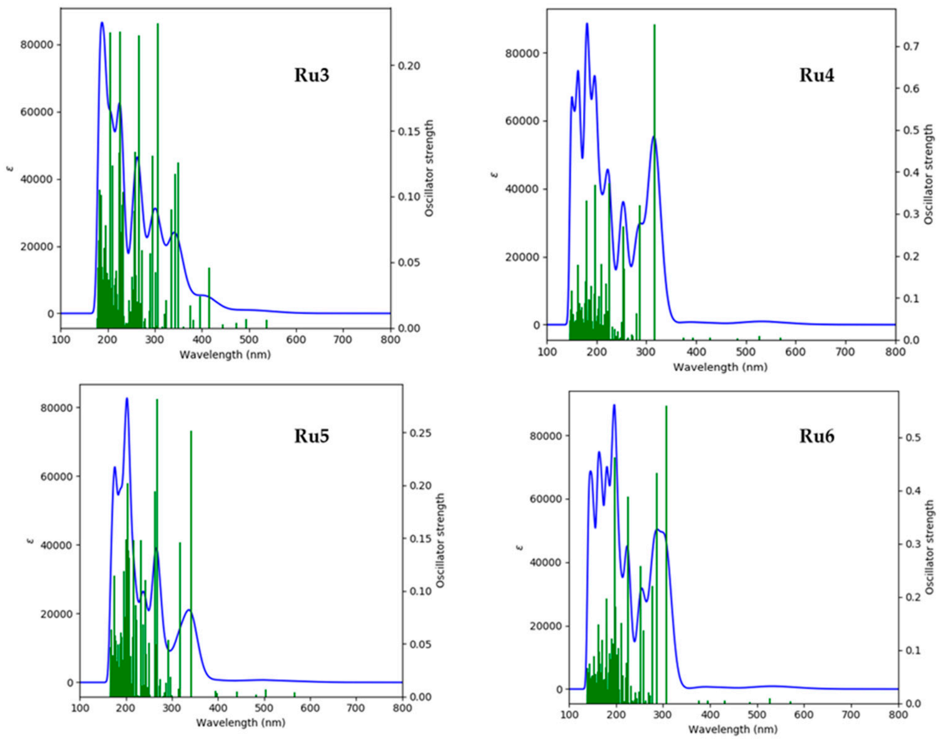

Figure S70. Theoretical UV–vis spectra of **Ru3-Ru6.**

Table S1. Selected bond angles (°) and lengths (Å) of compounds **3** and **4**.

Angles (°)		
**Atoms**	**3**	**4**
**H3–C3=N**	119.37	118.86
**N–N=C15**	104.24	105.18
**N-C5=C22**	110.47	108.79
**N-C19=C21**	107.80	109.01
**C21-C4=C9**	119.25	118.02
**C22-C16=C12**	132.24	129.49
**C16=C12-C15**	105.43	104.64
**C12-C15=N**	111.56	131.26
**C21-C22=C5**	106.58	106.80
**C20-C3=N**	121.26	122.24
**Bond lengths (Å)**		
C3–N	1.276	1.264
N–H1	0.880	0.881
**C5-N**	1.374	1.398
**N-N (py)**	1.349	1.368
**N-C15**	1.398	1.419
**C16-C22**	1.441	1.443
**C22-C21**	1.403	1.442
**C21-C19**	1.414	1.416
**C12-C16**	1.382	1.403

Table S2. Experimental and theoretical vibrational frequencies (cm^{-1}) in the infrared region of ligands and ruthenium complexes.

Vibrational mode	3			Ru3			4			Ru4			5			Ru5			6			Ru6		
	Exp	Teo	%PED	Exp	Teo	%PED	Exp	Teo	%PED	Exp	Teo	%PED	Exp	Teo	%PED	Exp	Teo	%PED	Exp	Teo	%PED	Exp	Teo	%PED
ν-OH	3149	3080	98	3252	3057	100	-	-	-	-	-	-	-	-	-	-	-	-	-	-	-	-	-	-
ν-NH_{indol}	3401	3522	99	3401	3533	99	3364	3524	100	3335	3563	100	3313	3524	100	3259	3563	98	3337	3524	24	3551	3563	99
ν-$NH_{pirazol}$	3387	3510	100	3127	3184	99	3265	3512	100	3152	3472	99	3114	3511	100	3216	3468	98	3214	3511	27	3282	3468	100
ν-C-H_{indol}	3000	3065	99	3028	3081	32	3007	3036	93	3053	3095	94	3001	3064	42	3071	3095	89	3062	3065	17	3058	3095	94
$ν_{as}$-CH_3	-	-	-	2943	2985	36	2944	2947	91	2956	2982	100	2941	2928	50	2961	2974	99	2964	2961	18	2929	2989	99
$ν_s$- CH_3	-	-	-	2833	2910	78	2886	2888	91	2869	2918	90	2832	2881	45	2833	2908	74	2873	2887	11	2837	2919	18
ν-C=N; C=C; C-H; δ -H-C=C; δ -H-N=N; δ-C=N=N	1621	1598	70	1617	1597	49	1614	1607	41	1621	1632	11	1615	1609	59	1616	1640	14	1611	1614	14	1621	1643	12
ν-C=C; ν-N-C; ν-H-N; ν-C-H, ν-C=C	1598	1544	63	1566	1550	29	1600	1540	60	1504	1583	33	1577	1549	10	1511	1591	55	1597	1539	10	1596	1587	39
ν-C=C_{p-cym}; -C-H_{p-cym}	-	-	-	1571	1510	21	-	-	-	1568	1515	32	-	-	-	1362	1380	49	-	-	-	1511	1545	21
$ν_s$- CH_3 $_{metoxi}$; δ -H-C-O; δ -H-N=N; δ -H-N=C; δ -H-C=C;	-	-	-	-	-	-	1432	1430	40	1411	1418	13	1420	1406	14	1429	1445	10	1239	1241	19	1452	1442	26
δ-C-H_{imine}	1446	1406	46	1360	1335	14	1264	1283	60	1386	1367	17	1326	1336	10	1317	1367	63	1313	1332	16	1339	1370	26
δ-N-$H_{pyrazole}$	975	963	59	1274	1240	14	1160	1141	61	1261	1264	44	1160	1145	31	1270	1266	19	1341	1376	14	1256	1242	21
ν-H_3C-O	-	-	-	-	-	-	1023	1022	14	1007	1014	53	1035	1033	37	1033	1030	52	1015	1011	21	1019	1008	47
Υ-N-$H_{pyrazole}$	656	666	55	744	715	10	675	665	40	639	633	32	663	655	70	678	647	24	615	666	10	604	643	36
ν-Ru-N+ ν-Ru-Caromat	-	-	-	473	468	16	-	-	-	427	426	18	-	-	-	416	426	10	-	-	-	395	476	10
$ν_s$-Ru-Cl_2 + ν-Ru-Caromat	-	-	-	292	278	17	-	-	-	323	266	11	-	-	-	289	319	20	-	-	-	318	325	10
$ν_{as}$-Ru-Cl_2 + ν-Ru-C-aromat	-	-	-	242	224	15	-	-	-	292	273	20	-	-	-	235	274	21	-	-	-	285	274	10

Table. S3. Experimental and theoretical vibrational frequencies (cm^{-1}) in the RAMAN of ligands and ruthenium complexes.

**Vibrational mode**	**Compounds**															
	**3**		**Ru3**		**4**		**Ru4**		**5**		**Ru5**		**6**		**Ru6**	
	**Exp**	**Teo**	**Exp**	**Teo**	**Exp**	**Teo**	**Exp**	**Teo**	**Exp**	**Teo**	**Exp**	**Teo**	**Exp**	**Teo**	**Exp**	**Teo**
**ν-C=N**	1613	1598	1611	1597	1604	1607	1617	1632	1608	1609	1609	1640	1617	1614	1601	1643
**ν-C=C$_{indol}$**	1580	1546	1574	1554	1583	1545	1584	1582	1583	1571	-	1587	1592	1545	1582	1617
**ν-C=C$_{p\text{-}cym}$**	-	-	1520	1510	-	-	1597	1544	-	-	1530	1545	-	-	1582	1545
**ν$_s$- CH$_{3\ metoxi}$**	-	-	-	-	1421	1415	1433	1458	1420	1406	1465	1486	1419	1421	1444	1451
**δ-C-H$_{imina}$**	1319	1331	1322	1324	1273	1283	1269	1367	1359	1336	1279	1367	1332	1332	1261	1370
**δ-N-H$_{pirazol}$**	1341	1374	1231	1240	938	962	1269	1230	1164	1145	1279	1250	957	960	1238	1228
**ν-H$_3$C-O**	-	-	-	-	1001	1013	1025	1021	1008	1033	1161	1127	1005	1001	1029	1010
**ϒ-N-H$_{pirazol}$**	640	666	700	715	533	502	648	633	592	544	691	710	587	666	630	643
**ν-Ru-N+ ν-Ru-C**	-	-	465	468	-	-	446	552	-	-	445	447	-	-	396	306
**ν$_{as}$-Ru-Cl$_2$ + ν-Ru-C-aromat**	-	-	359	367	-	-	343	372	-	-	350	339	-	-	310	274
**ν$_s$ -Ru-Cl$_2$ + ν-Ru-C-aromat**	-	-	286	278	-	-	317	319	-	-	312	318	-	-	262	265

Table S4. Experimental electronic absorption wavelengths and important Bases of Schiff and their complexes.

Compounds	Exp		B3LYP/6-311++g(d,p)				
	λ	Log(ε)$_{exp}$	λ	E (cm^{-1})	Log(ε)$_{teo}$	f	Contributions
**3**	297	4.09	296	33651	4.46	0.550	H-3→LUMO (82%); H-2→LUMO (2%)
	343	4.02	337	29580	4.41	0.444	H-1→LUMO (95%); H-3→LUMO (2%)
**Ru3**	297	4.13	298	33420	4.44	0.0004	H-1→L+4 (62%); H-3→L+4 (3%); H-2→L+4 (9%); H-1→L+3 (6%)
	344	4.02	342	29174	4.30	0.117	H-3→LUMO (39%); H-2→L+2 (13%); HOMO→L+2 (29%)
	447	2.41	444	22491	4.28	0.0025	H-5→L+2 (44%); H-1→L+2 (15%); HOMO→L+2 (12%); H-5→LUMO (9%)
**4**	287	4.02	285	34995	4.15	0.0012	H-2→LUMO (94%); HOMO→L+1 (3%)
	340	4.05	337	29648	3.59	0.526	H-1→LUMO (96%)
**Ru4**	286	4.05	286	34914	4.47	0.3201	H-3→L+1 (10%); H-1→LUMO (20%); HOMO→LUMO (32%)
	341	4.27	375	26607	3.55	0.0055	H-5→L+2 (45%); H-2→L+2 (29%); H-15→L+2 (3%); H-14→L+2 (3%); H-12→L+2 (3%)
	447	2.60	482	20701	2.67	0.0027	H-5→LUMO (21%); H-5→L+1 (20%); H-3→L+2 (24%)
**5**	283	4.12	287	34674	4.21	0.056	HOMO→L+1 (83%); H-1→L+1 (8%)
	365	4.04	366	27290	4.29	0.044	H-1→LUMO (93%); HOMO→LUMO (5%)
**Ru5**	283	4.26	287	34754	4.29	0.013	H-1→L+10 (13%); HOMO→L+6 (16%)
	365	3.99	341	29242	4.19	0.2511	H-4→LUMO (13%); HOMO→LUMO (69%)
	447	2.50	441	22646	2.79	0.0046	H-10→L+2 (10%); H-3→L+2 (21%); H-1→L+2 (46%)
**6**	328	4.38	325	30690	4.64	0.948	H-1→LUMO (93%)
**Ru6**	329	4.10	306	32509	4.27	0.5599	H-1→LUMO (28%); H-1→L+1 (14%); HOMO→LUMO (25%); HOMO→L+1 (10%)
	447	2.20	430	17360	3.95	0.0052	H-13→L+2 (11%); H-5→L+2 (27%); H-2→L+2 (50%); H-2→L+5 (3%)

Table S5. HOMO - LUMO energies and calculated global reactivity parameters of Schiff and their complexes calculated via B3LYP/6-311Gþþ (d, p) method.

$\Delta E = E_{LUMO} - E_{HOMO}$; $IP = -E_{HOMO}$; $EA = -E_{LUMO}$; $\chi = \frac{(E_{HOMO}+E_{LUMO})}{2}$; $\eta = \frac{E_{LUMO}-E_{HOMO}}{2}$;
$\sigma = \frac{1}{\eta}$; $\omega = \frac{\mu^2}{2\eta}$; $\eta = \frac{-(E_{HOMO}+E_{LUMO})}{2}$

**Parameters**	**3**	**Ru3**	**4**	**Ru4**	**5**	**Ru5**	**6**	**Ru6**
$E(Homo)$	-5,861	-5,846	-5,778	-6,883	-5,791	-6,773	-5,815	-7,130
E **(Lumo)**	-2,028	-2,323	-1,882	-0,766	-2,078	-1,037	-1,892	-1,070
ΔE **(Homo)-**E **(Lumo)**	3,833	3,523	3,896	6,116	3,713	5,735	3,923	6,060
**Electronegativity(**X**)**	3,944	4,084	3,830	3,824	3,934	3,905	3,853	4,100
**Chemical hardness**	1,916	1,761	1,948	3,058	1,857	2,868	1,962	3,030
**Chemical softness**	0,522	0,568	0,513	0,327	0,539	0,349	0,510	0,330
**IP**	5,861	5,846	5,778	6,883	5,791	6,773	5,815	7,130
**EA**	2,028	2,323	1,882	0,766	2,078	1,037	1,892	1,070
**Chemical potencial(μ)**	-3,944	-4,084	-3,830	-3,824	-3,934	-3,905	-3,853	-4,100
**Electrophilicity index(w-)**	-6,271	-6,997	-5,924	-4,686	-6,368	-4,970	-5,957	-5,203
**Electrophilicity index (w+)**	17,473	3,714	-27,904	-0,798	8,756	-1,041	-26,541	-1,072

Table S6. ^{1}H-NMR and ^{13}C-NMR experimental shifts for the Schiff bases and their complexes

ID atom	3		Ru3		4		Ru4		5		Ru5		6		Ru6	
	^{1}H	^{13}C	^{1}H	^{13}C	^{1}H	^{13}C	^{1}H	^{13}C	^{1}H	^{13}C	^{1}H	^{13}C	^{1}H	^{13}C	^{1}H	^{13}C
**OH**	13.313	-	-	-	-	-	-	-	-	-	-	-	-	-	-	-
**H-1**	12.998	-	12.740	-	12.852	-	12.803	-	12.803	-	12.735	-	12.852	-	12.847	-
**H-2**	11.496	-	11.449	-	11.462	-	11.453	-	11.453	-	11.415	-	11.462	-	11.457	-
**H-3; C-3**	9.233	162.852	9.152	154.782	9.226	154.349	8.914	159.573	8.914	159.573	9.115	154.444	9.226	154.349	9.220	154.456
**H-4; C-4**	7.979	119.837	8.020	128.405	7.966	119.980	8.170	119.877	8.170	119.877	8.010	128.398	7.966	119.980	7.950	121.983
**H-5; C-5**	7.849	123.862	7.943	120.305	7.820	123.803	7.814	123.580	7.814	123.580	7.969	120.220	7.820	123.803	7.817	123.801
**H-6; C-6**	7.659	132.719	7.805	123.720	7.585	110.074	7.589	109.466	7.589	109.466	7.792	123.723	7.585	110.074	7.580	110.068
**H-7; C-7**	7.488	112.461	7.455	119.911	7.100	114.104	7.467	124.119	7.467	124.119	7.450	117.718	7.100	114.104	7.121	114.119
**H-8; C-8**	7.421	133.423	7.160	112.348	7.478	112.343	7.467	111.862	7.467	111.862	7.146	112.265	7.478	112.343	7.460	112.303
**H-9; C-9**	7.209	122.377	7.160	122.254	7.790	122.274	7.198	122.314	7.198	122.314	7.146	122.153	7.790	122.274	7.186	122.229
**H-10; C-10**	7.162	120.346	6.679	107.018	7.160	119.919	7.129	120.305	7.129	120.305	6.695	119.750	7.160	119.919	7.152	119.916
**H-11; C-11**	7.008	119.690	6.679	98.666	7.140	120.298	7.114	112.429	7.114	112.429	6.672	98.622	7.140	120.298	7.121	120.214
**H-12; C-12**	6.959	92.221	6.679	93.030	6.754	93.214	6.801	92.257	6.801	92.257	6.657	92.980	6.754	93.214	6.749	92.916
**H-13; C-13**	-	-	3.928	56.012	3.885	56.755	3.886	56.112	3.886	56.112	3.929	56.033	3.885	56.755	3.886	56.768
**H-14; C-14**	-	-	3.865	55.316	3.798	55.941	3.852	55.879	3.852	55.879	3.867	56.334	3.798	55.941	3.797	55.950
**H-23; C-23**	-	-	-	-	-	-	-	-	-	-	5.819	85.980	-	-	5.817	86.832
**H-24; C-24**	-	-	-	-	-	-	-	-	-	-	5.773	86.835	-	-	5.771	85.975
**H-25; C-25**	-	-	-	-	-	-	-	-	-	-	2.813	30.446	-	-	2.834	30.448
**H-26; C-26**	-	-	-	-	-	-	-	-	-	-	2.090	18.338	-	-	2.089	18.338
**H-27; C-27**	-	-	-	-	-	-	-	-	-	-	1.194	21.965	-	-	1.193	21.953
**H-30, C-30**	6.988	117.086	-	-	-	-	-	-	-	-	-	-	-	-	-	-
**C-15**	-	160.731	-	163.975	-	160.015	-	160.140	-	160.140	-	164.083	-	160.015	-	154.653
**C-16**	-	157.355	-	161.178	-	154.266	-	152.09	-	152.09	-	161.229	-	154.266	-	154.281
**C-17**	-	-	-	160.469	-	153.710	-	149.541	-	149.541	-	154.296	-	153.710	-	153.709
**C-18**	-	139.653	-	154.831	-	139.282	-	139.087	-	139.087	-	154.197	-	139.282	-	139.785
**C-19**	-	136.839	-	136.825	-	136.821	-	136.841	-	136.841	-	136.821	-	136.821	-	136.819
**C-20**	-	124.889	-	117.804	-	124.868	-	129.850	-	129.850	-	117.795	-	124.868	-	124.944
**C-21**	-	124.846	-	124.845	-	125.060	-	124.949	-	124.949	-	120.101	-	125.060	-	125.052
**C-22**	-	105.451	-	107.018	-	105.734	-	105.801	-	105.801	-	107.067	-	105.734	-	106.859
**C-28**	-	-	-	-	-	-	-	-	-	-	-	106.852	-	-	-	111.943
**C-29**	-	-	-	-	-	-	-	-	-	-	-	100.583	-	-	-	100.567
